# Enrichment of prevotella melaninogenica in the lower respiratory tract links to checkpoint inhibitor pneumonitis and radiation pneumonitis

**DOI:** 10.3389/fcimb.2025.1594460

**Published:** 2025-10-03

**Authors:** Jiajun Chen, Qiong Xu, Liyan Zhang, Donglei Zhang, Xueling Wu

**Affiliations:** ^1^ Department of Respiratory and Critical Care Medicine, Ren Ji Hospital, Shanghai Jiao Tong University School of Medicine, Shanghai, China; ^2^ Department of Thoracic Surgery, Ren Ji Hospital, Shanghai Jiao Tong University School of Medicine, Shanghai, China

**Keywords:** lower respiratory tract, microbiome, checkpoint inhibitor pneumonitis, radiation pneumonitis, metagenomic next-generation sequencing

## Abstract

**Background:**

Checkpoint inhibitor pneumonitis (CIP) and radiation pneumonitis (RP) lead to anti-cancer therapy discontinuation and poor diagnosis. The human microbiome is related to various respiratory diseases. However, the role of the lung microbiome in CIP and RP remains unknown. Our study aimed to explore the lower respiratory tract (LRT) microbiome in CIP/RP patients.

**Methods:**

The study enrolled 61 patients with pneumonitis or pneumonia, including 23 with CIP/RP, and 38 with lung cancer with pneumonia (LC-P). Metagenomic next-generation sequencing (mNGS) was performed to identify the microbiota in bronchoalveolar lavage fluid (BALF), and bioinformatics methods were used to compare the microbial differences between CIP/RP and LC-P groups. Correlation analysis was conducted to explore the relationship between LRT microbiota and clinical features.

**Results:**

The *Prevotella* was the dominant genus in both groups. The *Prevotella melaninogenica*, which belongs to the *Prevotella* genus, was the dominant species in the CIP/RP group and the second most abundant species in the LC-P group. Compared to the LC-P group, the CIP/RP group had significantly high levels of *Prevotella melaninogenica* species and lymphocyte percentage in BALF but significantly low levels of lymphocytes, eosinophils and albumin in peripheral blood. In addition, the *Prevotella melaninogenica* species had a negative correlation with peripheral blood lymphocytes.

**Conclusion:**

The enrichment of *Prevotella melaninogenica* species in LRT and a decreased level of peripheral blood lymphocytes are associated with CIP/RP.

## Introduction

Immune checkpoint inhibitors targeting PD-1/PD-L1 have changed the treatment landscape for oncology, but their clinical benefits are tempered by immune-related adverse events (Suresh et al., 2018a; Shankar et al., 2020; Sun et al., 2023). Among these, checkpoint inhibitor pneumonitis (CIP) affects 2.49–19% of patients, often leading to therapy discontinuation and poor prognosis (Naidoo et al., 2017; Suresh et al., 2018b; Suresh et al., 2019; Tiu et al., 2022; Yin et al., 2022). Similarly, radiation pneumonitis (RP) occurs in 1–25% of thoracic radiotherapy recipients, with shared features of dysregulated inflammation and fibrosis complicating differential diagnosis (Hanania et al., 2019). In addition, CIP is more common in patients receiving curative-intent radiotherapy followed by anti-PD-1/PD-L1 agents (Shaverdian et al., 2017; Voong et al., 2019). However, it turns out to be difficult to distinguish CIP from RP (Rahi et al., 2021).

The human microbiome is related to various respiratory diseases (Frayman et al., 2024; Özçam and Lynch, 2024; Song et al., 2024). For instance, the gut microbiota influences chronic obstructive pulmonary disease (COPD) development and fecal microbiota transplantation restores the pathogenesis of COPD (Lai et al., 2022). The gut protist *Tritrichomonas musculis* induces the migration of gut-derived lymphoid cells to the lung and further promotes steady state eosinophilia, which exacerbates asthma and hinders the systemic dissemination of pulmonary Mycobacterium tuberculosis (Burrows et al., 2025). The gut microbiome shapes the immune system and may play a protective role in respiratory diseases, suggesting that managing the gut microbiome represents a powerful way to prevent and treat respiratory diseases (Perdijk et al., 2024). However, the role of the lung microbiome in cancer treatment-related pneumonitis, particularly CIP and RP, remains unknown. Exploring lung microbial dysbiosis is crucial to understanding the occurrence of CIP/RP on microbial terms and managing the microbial imbalance may be a potential therapy for CIP/RP.

Our study aimed to explore the LRT microbiome in CIP/RP patients, analyze the microbial composition and diversity, compare the microbial differences, and further explore the relationship between LRT microbiome and clinical features.

## Methods

### Recruitment of patients

This retrospective study was conducted at Renji Hospital, Shanghai Jiao Tong University School of Medicine. A total of 61 patients were enrolled from 20 June 2021 to 20 October 2024. Among them, 23 were classified as CIP/RP group (of whom, 16 had CIP and 7 had RP), and 38 patients were diagnosed with LC-P. Inclusion criteria for CIP or RP included: (1) the cancer patients had received immunotherapy or radiotherapy; (2) imaging studies showed new pulmonary infiltrates (radiologic patterns of CIP include cryptogenic organizing pneumonia, ground glass opacities and interstitial pneumonia, while radiologic features of RP are ground glass opacities or consolidation conforming precisely to the shape of the radiation field.); (3) LRT infection or lung tumor progression was excluded; and (4) the patients were not treated with antibiotics or steroid within 2 weeks.

### BALF collection

BALF samples were collected from all 61 patients according to the standard procedures. The lung was lavaged with 100mL of sterile saline solution and the BALF recovery rate was more than 35%. BALF samples for further mNGS were then transported to the hospital laboratory under cold-chain conditions. Clinical information and laboratory results were also collected when the patients were sampled.

### Nucleic acid extraction, library preparation, sequencing, and bioinformatics analysis

The TIANamp Magnetic DNA Kit (Tiangen) was used to extract DNA. Quantity and quality of DNA were assessed using the Qubit and NanoDrop (Thermo Fisher Scientific), respectively. DNA libraries were prepared using the Hieff NGS C130P2 OnePot II DNA Library Prep Kit for MGI (Yeasen Biotechnology) according to the manufacturer’s protocols. Agilent 2,100 was used for quality control and DNA libraries were 50 bp single-end sequenced on MGISEQ-200. Raw sequencing data were split by bc12fastq2 (version 2.20), and high-quality sequencing data were generated using Trimmomatic (version 0.36) by removing low-quality reads, adapter contamination, duplicated and shot (length, 36bp) reads. Human host sequences were subtracted by mapping to human reference genome (hs37d5) using bowtie2 (version 2.2.6). Reads that could not be mapped to the human genome were retained and aligned with the microorganism genome database for microbial identification by Kraken (version 2.0.7), and species abundance estimating by Bracken (version 2.5.0). The microorganism genome database contained genomes or scaffolds of bacteria, fungi, viruses, and parasites (download from GenBank release 238, ftp://ftp.ncbi.nlm.nih.gov/genomes/genbank/).

### Statistical analysis

We performed the microbial diversity analysis using R software (version 4.0.1). The alpha-diversity was assessed by taxonomic profiles, and the beta-diversity was estimated by Bray- Curtis distance. PERMANOVA (vegan) was used to analyze beta-diversity differences. Differences of the relative genus abundances were tested by the Kruskal-Wallis test (Kruskal.test package). Only genera with greater than 1% mean abundance and 40% prevalence were compared. Linear discriminant analysis (LDA) effect size (LEfSe) was performed to assess the statistical differences of the relative abundance of microorganisms between CIP/RP and LC-P patients. Spearman’s correlations between clinical indicators and relative genus abundances were determined by R package cor. test and adjusted by false discovery rate. A random forest binary classification model integrating key microbes and significantly clinical indicators was assessed by Receiver Operating Characteristic Curve.

Student’s *t*-test or Mann-Whitney *U* test was used to compare the continuous variables. For categorical variables, Chi-square test or Fisher’s exact test was used to explore the association. All significance tests were two-tailed and a P value < 0.05 was considered statistically significant.

### Ethical approval

This study was conducted in accordance with the Declaration of Helsinki and was approved by the Ethics Committee of Renji Hospital, Shanghai Jiao Tong University School of Medicine, Shanghai, China (KY2021-102-B). Informed consent was obtained from all patients.

## Results

### Demographic information of participants

The study included 61 patients. Among these patients, 23 were classified as grade1–2 CIP/RP (of whom, 16 had CIP and 7 had RP), while 38 were confirmed to have LC-P. The demographic and clinical characteristics of the patients are detailed in **Table 1**. The patients in the CIP/RP group were younger than those in the LC-P group. Comorbidities comprised chronic obstructive pulmonary disease (COPD, 17.4%), hypertension (13%) and diabetes (13%) in the CIP/RP group. The CIP/RP group had lower levels of lymphocytes (P = 0.001), EOS (P = 0.008), and ALB (P = 0.006) in peripheral blood than those in the LC-P group. In BALF, the percentage of lymphocyte was significantly high in the CIP/RP group (P = 0.010). The CIP/RP group also had higher levels of C-reactive protein (CRP), erythrocyte sedimentation rate (ESR), Krebs Von den Lungen-6 (KL-6), and D-dimer, but no significant differences were observed in these clinical indicators.

**Table 1:** Characteristics of the patients.

Items	CIP/RP (n=23)	LC-P (n=38)	*P* value
Female, n (%)	3 (13%)	7 (18.4%)	0.847
Age (years)	64.91 (7.09)	68.30 (5.67)	**0.046**
Comorbidities			
COPD	4 (17.4)	11 (28.9)	0.310
Hypertension (%)	3 (13)	16 (42.1)	**0.018**
Diabetes (%)	3 (13)	3 (7.9)	0.513
Laboratory findings			
WBC (10^9^/L)	5.92 (4.65-8.52)	6.53 (5.28-8.10)	0.400
Neutrophils (10^9^/L)	4.06 (3.47-6.40)	4.53 (3.53-5.69)	0.732
Lymphocytes (10^9^/L)	0.67 (0.52-1.16)	1.26 (0.95-1.66)	**0.001**
NLR	5.58 (4.39-8.27)	3.61 (2.92-4.19)	**0.001**
EOS (10^9^/L)	0.04 (0.01-0.11)	0.13 (0.08-0.18)	**0.008**
BALF-N (%)	67.50 (30.00-80.75)	44.00 (14.75-72.00)	0.206
BALF-L (%)	6.00 (3.00-15.75)	3.50 (0.75-5.25)	**0.010**
CRP (mg/L)	23.68 (2.20-48.93)	4.78 (0.66-26.05)	0.107
PCT (ng/mL)	0.07 (0.05-0.22)	0.05 (0.02-0.08)	0.094
ESR (mm/h)	67.00 (10.00-76.00)	30.00 (9.00-65.00)	0.440
KL-6 (U/mL)	453.00 (251.25-866.50)	274.00 (188.25-516.25)	0.096
B lymphocytes (cells/uL)	50.10 (28.20-86.18)	143.50 (86.60-226.21)	**0.008**
T lymphocytes (cells/uL)	504.70 (410.50-659.93)	753.30 (588.51-1080.40)	**0.004**
Th (cells/uL)	218.00 (151.60-308.48)	461.50 (299.44-638.59)	**0.001**
Ts (cells/uL)	243.50 (147.95-342.98)	275.10 (174.86-416.95)	0.384
NK (cells/uL)	106.40 (49.15-277.70)	252.80 (136.29-351.77)	**0.001**
D-dimer (mg/L)	0.39 (0.21-1.07)	0.22 (0.16-0.51)	0.082
FDP (mg/L)	4.47 (3.10-6.10)	4.17 (2.91-5.50)	0.524
SCR (umol/L)	72.00 (20.11)	73.43 (16.88)	0.768
GLU (mmol/L)	6.50 (5.70-9.30)	6.60 (5.55-8.75)	0.994
ALB (g/L)	35.01 (5.47)	39.04 (3.86)	**0.006**
CAR	0.87 (0.06-1.82)	0.09 (0.02-0.54)	0.062
LDH (U/L)	208.00 (170.00-271.80)	207.00 (178.00-235.50)	0.520
ALT (U/L)	14.00 (11.00-41.00)	18.00 (13.75-33.50)	0.367
AST (U/L)	26.00 (20.00-33.00)	22.50 (20.00-28.00)	0.267

Statistically significant *P* < 0.05 values are in bold. COPD, chronic obstructive pulmonary disease; WBC, white blood cells; NLR, neutrophils to lymphocytes ratio; EOS, eosinophils; BALF, bronchoalveolar lavage fluid; BALF-N, neutrophile percentage in BALF; BALF-L, lymphocyte percentage in BALF; CRP, C-reactive protein; ESR, erythrocyte sedimentation rate; PCT, procalcitonin; KL-6, Krebs Von den Lungen-6; FDP, fibrin degradation products; SCR, serum creatine; GLU, glucose; ALB, albumin; CAR CRP to ALB ratio; LDH, lactate dehydrogenase; ALT, alanine aminotransferase; AST, aspartate aminotransferase.

### Lung microbial diversity and composition

**Figure 1:**
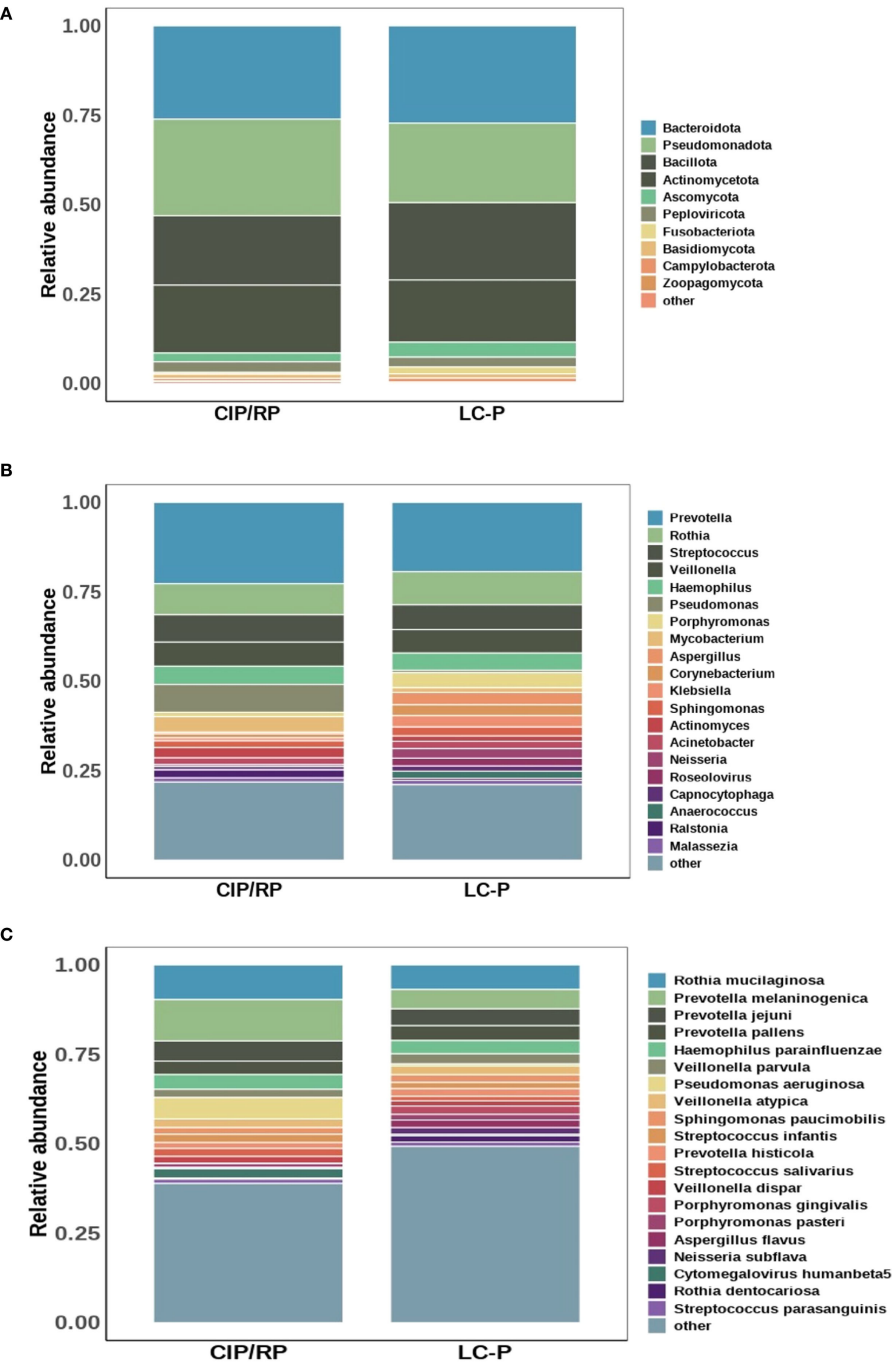
Comparison of the lung microbial profile between CIP/RP and LC-P groups. **(A)** Dominant phyla. **(B)** Dominant genera. **(C)** Dominant species.

At species level, alpha-diversity was based on ACE, Chao1, Shannon, and Simpson indexes (**Supplementary Figure 1A**). However, there were no significant differences in ACE (P = 0.63), Chao1 (P = 0.63), Shannon (P = 0.8), and Simpson (P = 0.92) indexes. Additionally, PCoA and PC analysis based on the Bray-Curtis distances also showed that no difference was observed in beta-diversity (P = 0.481 and P = 0.477, respectively) (**Supplementary Figure 1B**). We then analyzed the lung microbial composition in CIP/RP and LC-P groups. In the CIP/RP group, the top five phyla were *Pseudomonadota* (27.0%), *Bacteroidota* (26.1%), *Bacillota* (19.4%), *Actinomycetota*(19.0%) and *Peploviricota* (3.0%) (**Figure 1A**). The top five genera included *Prevotella* (22.7%), *Rothia* (8.7%), *Pseudomonas* (7.8%), *Streptococcus* (7.7%), and *Veillonella* (6.7%) (**Figure 1B**). The top five species were *Prevotella melaninogenica* (11.6%), *Rothia mucilaginosa* (9.7%), *Pseudomonas aeruginosa* (6.0%), *Prevotella jejuni* (5.6%) and *Haemophilus parainfluenzae* (4.1%) (**Figure 1C**). In the LC-P group, the top five phyla included *Bacteroidota* (27.2%), *Pseudomonadota* (22.2%), *Bacillota* (21.6%), *Actinomycetota* (17.4%) and *Ascomycota* (4.2%) (**Figure 1A**). The top five genera were *Prevotella* (19.4%), *Rothia* (9.2%), *Streptococcus* (6.9%), *Veillonella* (6.6%) and *Haemophilus* (4.8%) (**Figure 1B**). The top five species included *Rothia mucilaginosa* (6.9%), *Prevotella melaninogenica* (5.4%), *Prevotella jejuni* (4.7%), *Prevotella pallens* (4.1%) and *Haemophilus parainfluenzae* (3.7%) (**Figure 1C**).

### Differential microbiota analysis

**Figure 2:**
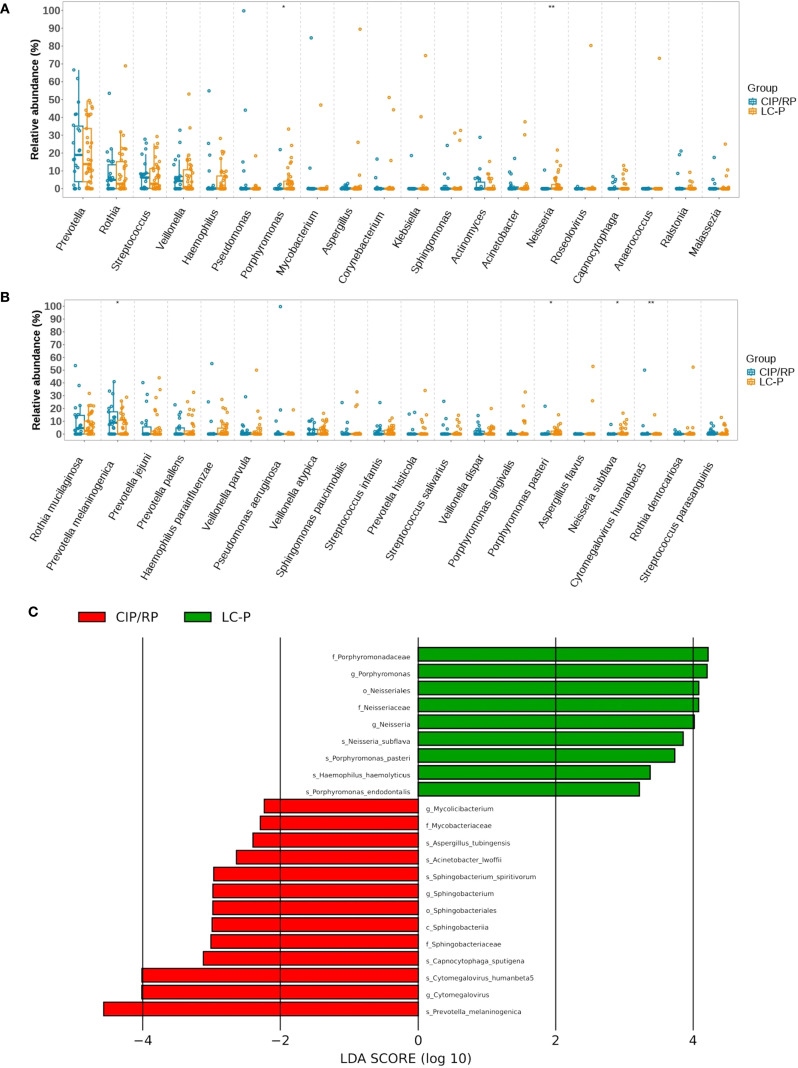
Differential relative abundances between CIP/RP and LC-P groups. **(A)** Differential relative abundance (log_10_) of top 20 genera. **(B)** Differential relative abundance (log_10_) of top 20 species. **(C)**. Linear discriminant analysis (LDA) effect size (LEfSe) analysis of the differential microbiota between CIP/RP and LC-P groups.

We further analyzed the differential relative abundance of top 10 phyla, top 20 genera, and top 20 species between CIP/RP and LC-P groups. At the phyla level, no significant differences were observed in the relative abundance of the top 10 phyla. At the genera level (**Figure 2A**), the relative abundance of *Porphyromonas* (P = 0.028) and *Neisseria* (P = 0.010) were significantly lower in the CIP/RP group than those in the LC-P group. No significant differences were observed in the relative abundance of the other genera between the two groups. At the species level (**Figure 2B**), the relative abundance of *Prevotella melaninogenica* (P = 0.018) and *Cytomegalovirus humanbeta5* (P = 0.010) were significantly higher in the CIP/RP group than those in the LC-P group. However, the relative abundance of *Neisseria subflava* (P = 0.024) and *Porphyromonas pasteri* (P = 0.045) were significantly lower in the CIP/RP group than those in the LC-P group. No significant differences were observed in the relative abundance of the other species between the two groups. 22 discriminative features were identified by LEfSe. Among them, 13 taxa were discriminative for the CIP/RP group and 9 taxa were discriminative for the LC-P group (**Figure 2C**). At the genera level, the *Cytomegalovirus* (LDA scores >4, P = 0.010) was significantly higher in the CIP/RP group while *Porphyromonas* (LDA scores >4, P = 0.028) and *Neisseria* (LDA scores >4, P = 0.010) were abundant in the LC-P group. At the species level, *Prevotella melaninogenica* (LDA scores >4, P = 0.018) and *Cytomegalovirus_humanbeta5* (LDA scores >4, P = 0.010) were significantly higher in the CIP/RP group while *Neisseria_subflava* (LDA scores >2, P = 0.024) and *Porphyromonas pasteri* (LDA scores >2, P = 0.045) were significantly more abundant in the LC-P group.

### Correlation between differential microbial taxa and clinical indicators

**Figure 3:**
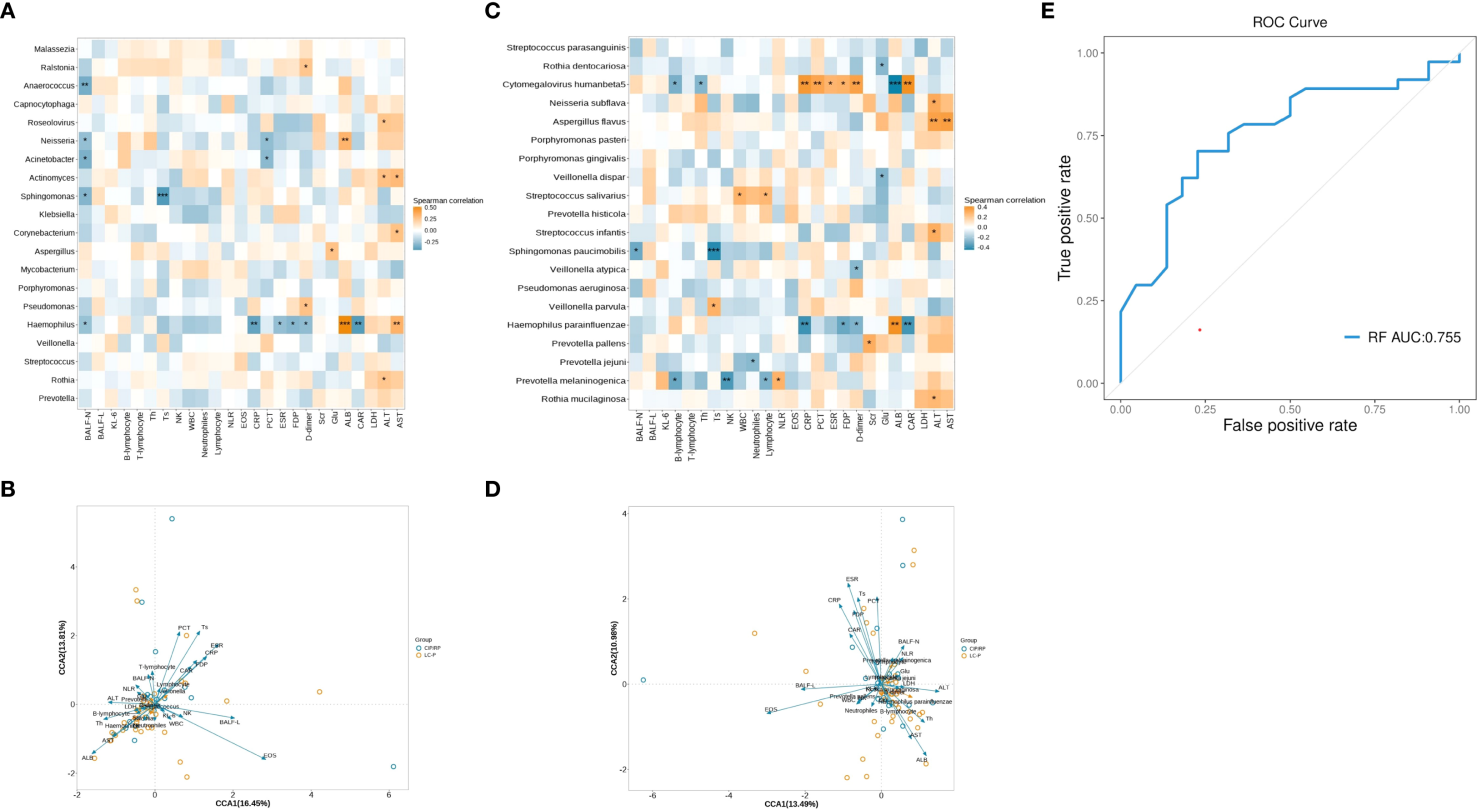
Correlation analysis between microbial taxa and clinical indicators. **(A)** Heatmap showing the correlation between top 20 genera and clinical indicators. **(B)** CCA analysis showing the relationship between genera and clinical indicators. The acute angle shows a positive correlation, and an obtuse angle shows a negative correlation. The longer arrow means a greater influence of the indicator.**(C)** Heatmap showing the correlation between top 20 species and clinical indicators. **(D)** CCA analysis showing the relationship between species and clinical indicators. **(E)** Receiver Operating Characteristic Curve for random forest binary classification model. *, *p* < 0.05; **, *p* < 0.01; ***, *p* < 0.001. WBC, white blood cells; NLR, neutrophils to lymphocytes ratio; EOS, eosinophils; BALF, bronchoalveolar lavage fluid; BALF-N, neutrophil percentage in BALF; BALF-L, lymphocyte percentage in BALF; CRP, C-reactive protein; ESR, erythrocyte sedimentation rate; PCT, procalcitonin; KL-6, Krebs Von den Lungen-6; FDP, fibrin degradation products; SCR, serum creatine; GLU, glucose; ALB, albumin; CAR CRP to ALB ratio; LDH, lactate dehydrogenase; ALT, alanine aminotransferase; AST, aspartate aminotransferase.

A Spearman correlation analysis was used to further explore the relationship between top 20 genera (or species) and clinical indicators. A two-dimensional heatmap showed the results (**Figures 3A, C**). We mainly focused on differential microbiota and clinical indicators with significant difference. At the genera level, the *Porphyromonas* had no correlation with all clinical indicators. The *Neisseria* showed a positive correlation with ALB. At the species level, the *Prevotella melaninogenica* had a negative correlation with B lymphocytes, NK, and lymphocytes in peripheral blood. The *Cytomegalovirus humanbeta5* showed a negative correlation with B lymphocytes and ALB in peripheral blood. The *Neisseria subflava* and *Porphyromonas pasteri* had no correlation with significantly different clinical indicators. Furthermore, a CCA analysis showed that the levels of peripheral blood EOS, ALB, and BALF-L were found to have a strong relation with the top 20 genera or species (**Figures 3B, D**). In addition, a random forest binary classification model was constructed. The model integrated *Prevotella melaninogenica* and significantly clinical indicators including peripheral blood lymphocyte, EOS, ALB and BALF-L, yielding an AUC of 0.755 (**Figure 3E**).

## Discussion

In this study, we explored the LRT microbiome in the CIP/RP patients, analyzed microbial composition and diversity, compared the differences between CIP/RP and LC-P groups, and further explored the relationship between LRT microbiome and clinical features. We found that the CIP/RP group had higher levels of *Prevotella melaninogenica* species and BALF-L but lower levels of lymphocytes, EOS, and ALB in peripheral blood. In addition, the *Prevotella melaninogenica* species had a negative correlation with the peripheral blood lymphocytes.

The *prevotella* is one of the most common genera among the bacteriome of healthy lung microbiome (Li et al., 2024). As the commensal bacterial microbiota colonized in healthy human airway, the gram-negative *prevotella* spp. are found to have weak inflammatory properties and be intrinsically tolerated by the respiratory immune system (Larsen et al., 2015). However, the alteration of the *prevotella* is related to occurrence and development of various diseases (Rofael et al., 2019; De Martin et al., 2021; Wang et al., 2022). De Martin et al. found that the relative abundance of *Prevotella melaninogenica* was increased in tonsil cancer (De Martin et al., 2021). Sylvia A.D. Rofael et al. used 16S rRNA gene sequencing to detect the respiratory pathogen in induced sputum collected from young adults born extremely preterm and found that the relative abundance of *prevotella*, particularly *prevotella melaninogenica* was significantly decreased (Rofael et al., 2019). Once colonizing the stomach, the *Prevotella melaninogenica* was associated with gastric inflammation or carcinogenesis (Xia et al., 2025). The relative abundance of *Prevotella melaninogenica* showed a significantly high level in the gastric juice of patients with gastric cancer and bile reflux gastritis and the *Prevotella melaninogenica* was found to induce gastric inflammation in mice, suggesting that the *Prevotella melaninogenica* may be associated with the gastric carcinogenesis (Wang et al., 2022).

The role of *Prevotella* in respiratory diseases is intricate (Larsen et al., 2015; Horn et al., 2022; Lu et al., 2024). Lung dysbiosis with decreased *prevotella* spp. and increased pathogenic *proteobacteria* in chronic airway diseases suggests that *prevotella* spp. play a protective role in chronic airway diseases (Larsen et al., 2015). Kadi J. Horn et al. showed that the *Prevotella melaninogenica* induced an innate immune response and reinforced protection against bacterial pathogen *Streptococcus pneumoniae* in a mouse lung co-infection model, highlighting airway *Prevotella* as a protective role in respiratory tract health (Horn et al., 2022). However, Fan Lu et al. reported that the *Prevotella melaninogenica* as an opportunistic pathogen may lead to immune dysregulation in immunocompromised patients with sepsis-induced acute lung injury (Lu et al., 2024). In this study, the *Prevotella* was the dominant genus in both groups, but no significant difference was observed between the two groups. The *Prevotella melaninogenica*, which belongs to the *Prevotella* genus, was the dominant species in the CIP/RP group and the second most abundant species in the LC-P group. Compared to the LC-P group, the CIP/RP group had significantly high levels of the *Prevotella melaninogenica* species. The enrichment of *Prevotella melaninogenica* may represent its pathogenicity in CIP/RP.

The CIP/RP patients have unique clinical features that consist of an increased lymphocyte percentage in BALF and a decreased level of lymphocytes in peripheral blood (Roberts et al., 1993; Zhou et al., 2020; Lin et al., 2021; Chen et al., 2024). In addition, the reduction of EOS and ALB in peripheral blood is related to the occurrence of CIP (Lin et al., 2021; Li et al., 2022). In this study, the CIP/RP group had a significantly high level of BALF-L but low levels of lymphocytes, EOS, and ALB in peripheral blood, which is consistent with previous studies. We also found that the *Prevotella melaninogenica* species had a negative correlation with peripheral blood lymphocytes, suggesting the interplay between LRT microbiota and clinical features.

This study has several limitations. First, the number of CIP/RP was small. A future study with large samples would be valuable to validate these findings. Second, the role of the other species, which were not abundant but significantly different, was not well understood. Finally, this study didn’t collect BALF and clinical features from healthy group and patients after the recovery of CIP/RP. Future research is needed to elucidate the interplay between LRT microbiota and clinical features in CIP/RP.

## Conclusion

The CIP/RP patients had an increased relative abundance of *Prevotella melaninogenica* species that showed a negative correlation with peripheral blood lymphocytes, suggesting that the enrichment of *Prevotella melaninogenica* species in LRT associated with a decreased level of peripheral blood lymphocytes may be a potential biomarker of diagnosis and treatment for CIP/RP.

## Data Availability

The original contributions presented in the study are included in the article/**Supplementary Material**, further inquiries can be directed to the corresponding author/s.

## Supplementary material

Supplementary Figure 1 Diversity of LRT microbial flora between CIP/RP and LC-P groups. **(A)** Alpha-diversity based on ACE, Chao1, Shannon, and Simpson indexes. **(B)** Beta-diversity based on PCoA and PC algorithm.
