# Supplementary figures and images for: Enrichment of prevotella melaninogenica in the lower respiratory tract links to checkpoint inhibitor pneumonitis and radiation pneumonitis (part 1 of 2)
Source: Front Cell Infect Microbiol. 2025 Oct 3;15:1594460. doi: 10.3389/fcimb.2025.1594460 (PMC12531214; doi:10.3389/fcimb.2025.1594460)

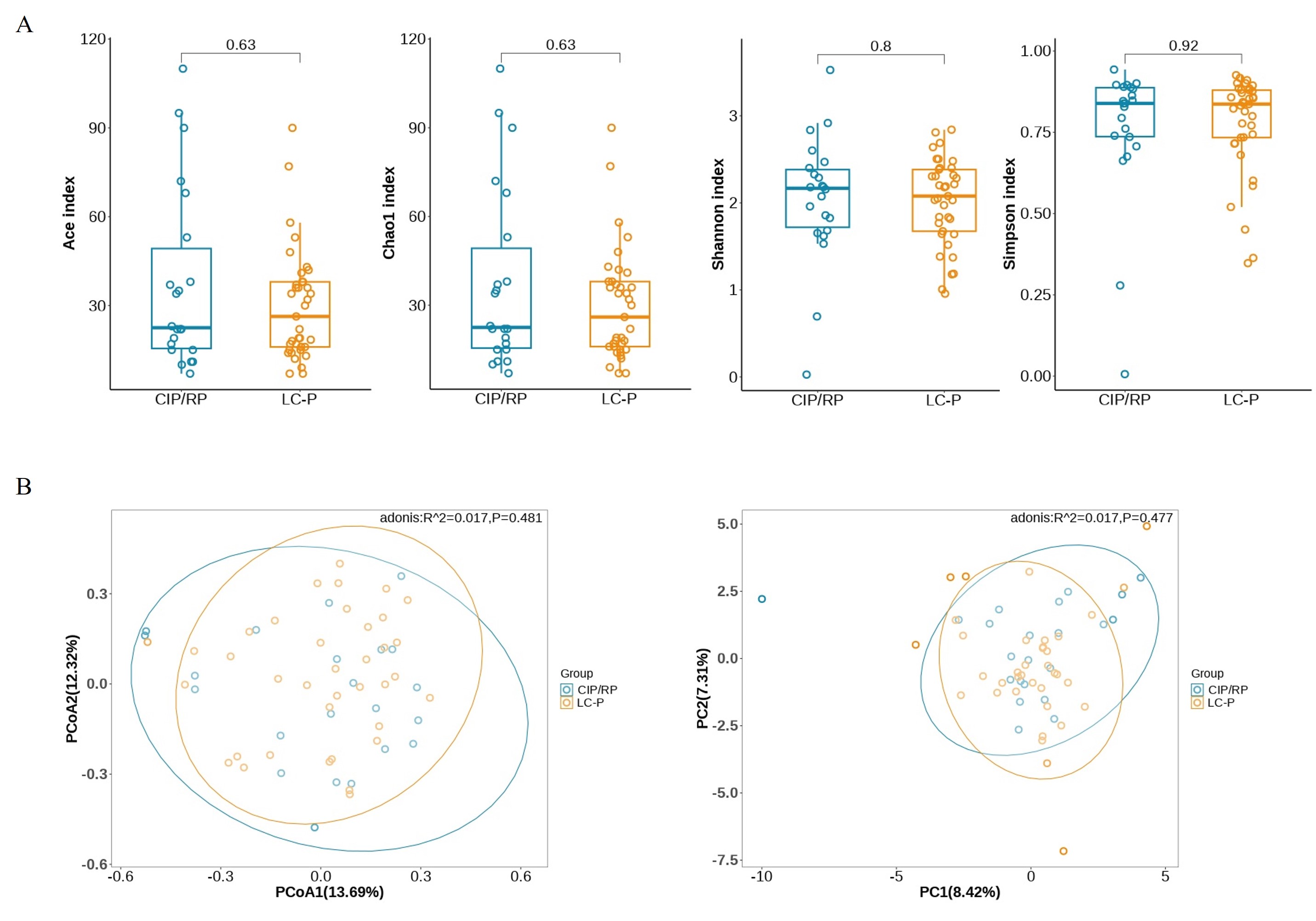

Supplement: Supplementary Figure 1 — Diversity of LRT microbial flora between CIP/RP and LC-P groups. (A) Alpha-diversity based on ACE, Chao1, Shannon, and Simpson indexes. (B) Beta-diversity based on PCoA and PC algorithm. [file Image1.jpg]

ROC Curve

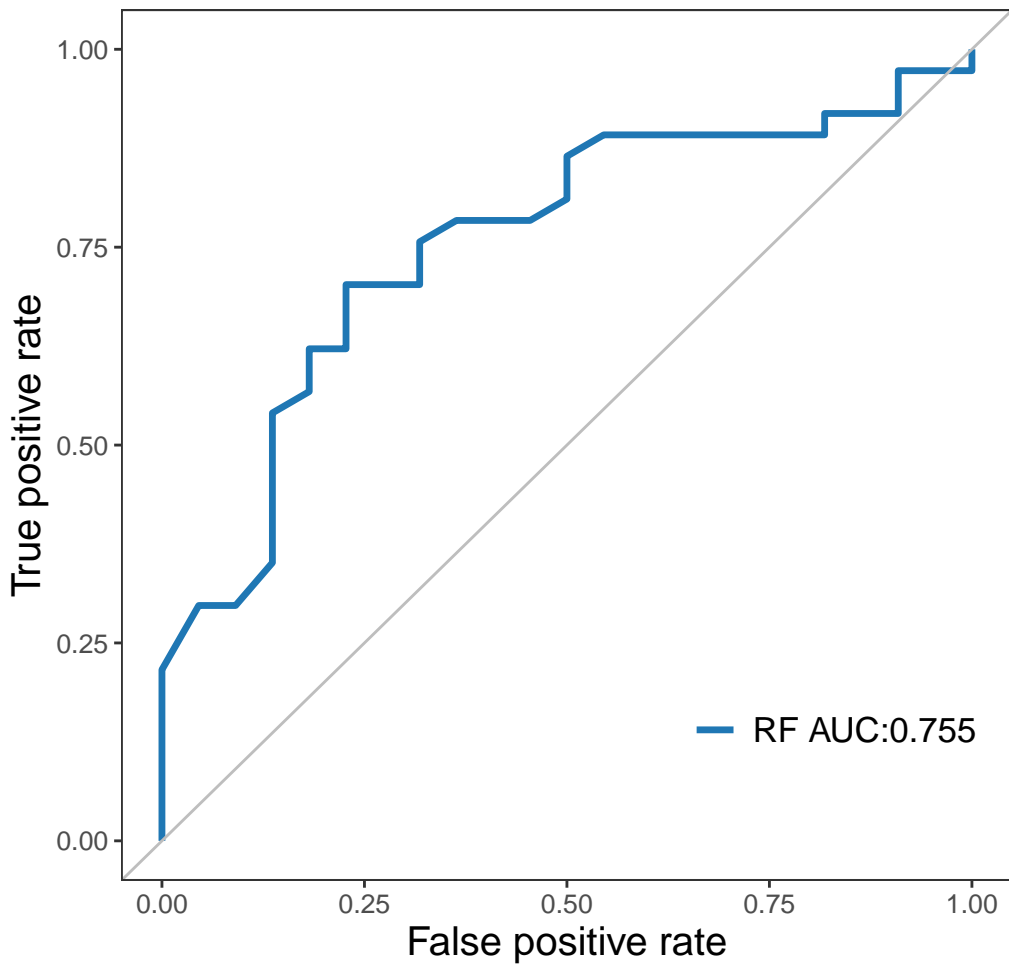

Supplement: Supplementary file 2 [file DataSheet1.zip › Data-all result/2025.8.23-审稿人意见随机森林结果/2025.8.23-审稿人意见随机森林/group1.pdf]

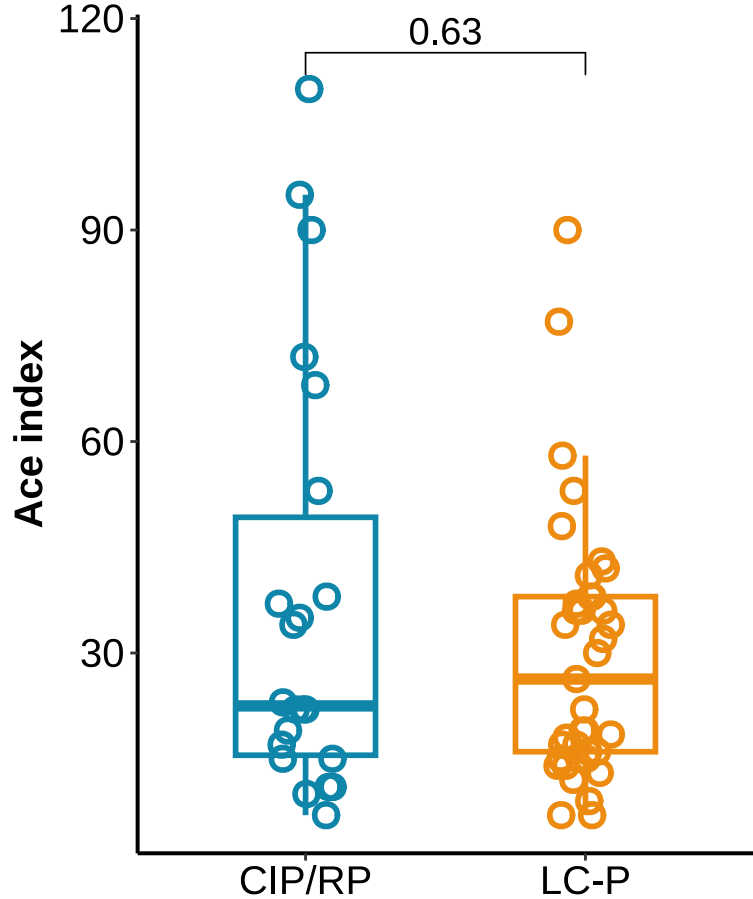

Supplement: Supplementary file 2 [file DataSheet1.zip › Data-all result/alpha_index/Group_ace_index_boxplot.pdf]

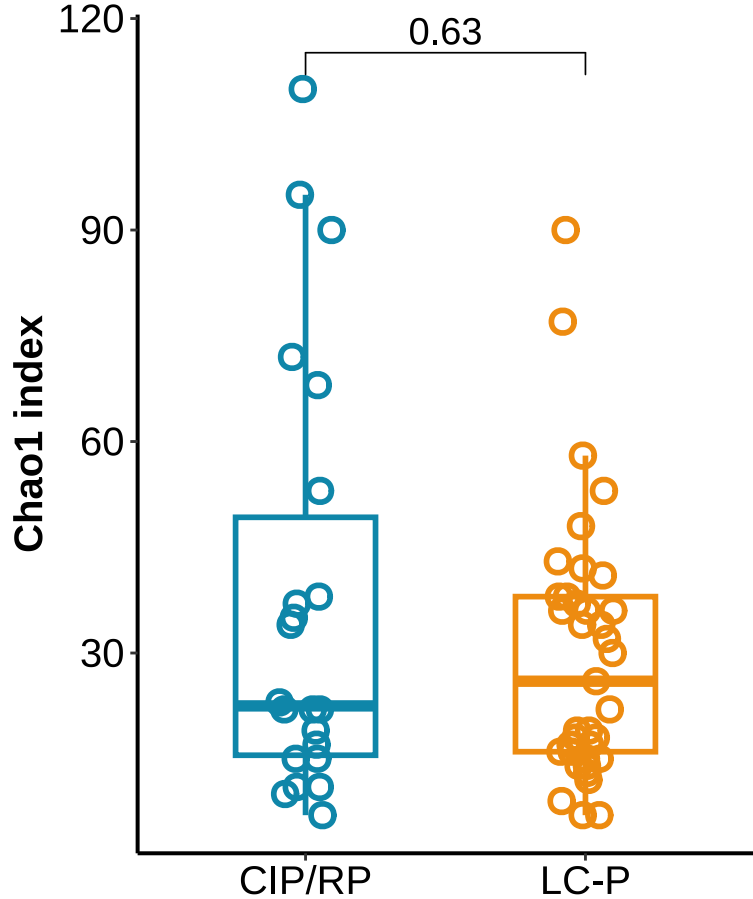

Supplement: Supplementary file 2 [file DataSheet1.zip › Data-all result/alpha_index/Group_chao1_index_boxplot.pdf]

Shannon index

0.8

3

2

1

0

CIP/RP

LC-P

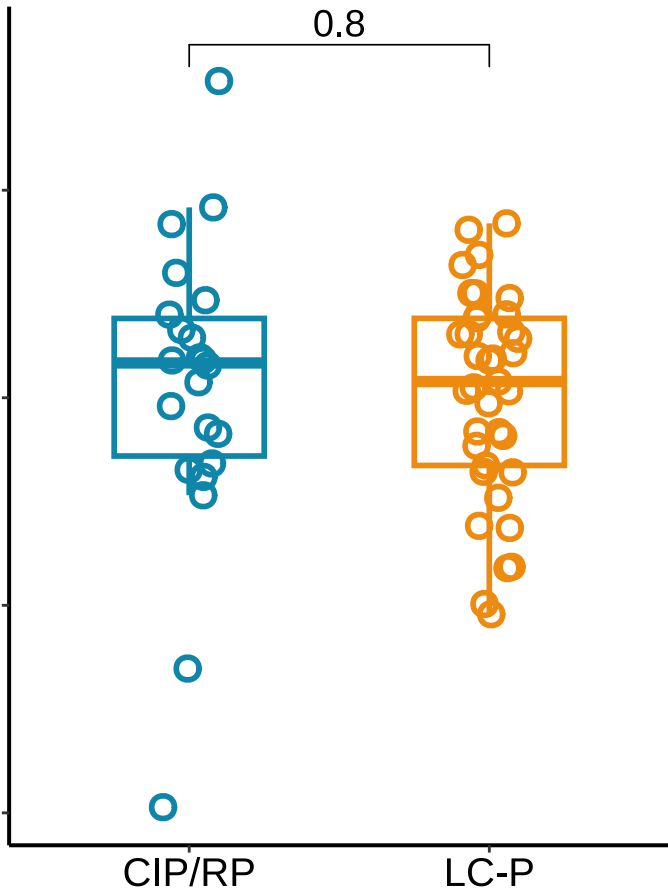

Supplement: Supplementary file 2 [file DataSheet1.zip › Data-all result/alpha_index/Group_shannon_index_boxplot.pdf]

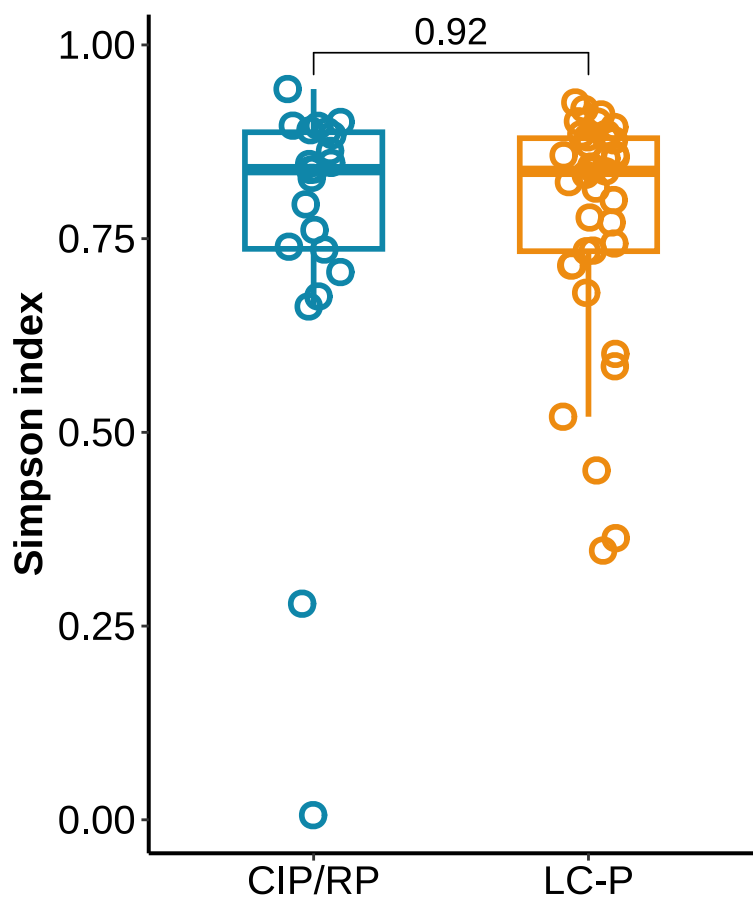

Supplement: Supplementary file 2 [file DataSheet1.zip › Data-all result/alpha_index/Group_simpson_index_boxplot.pdf]

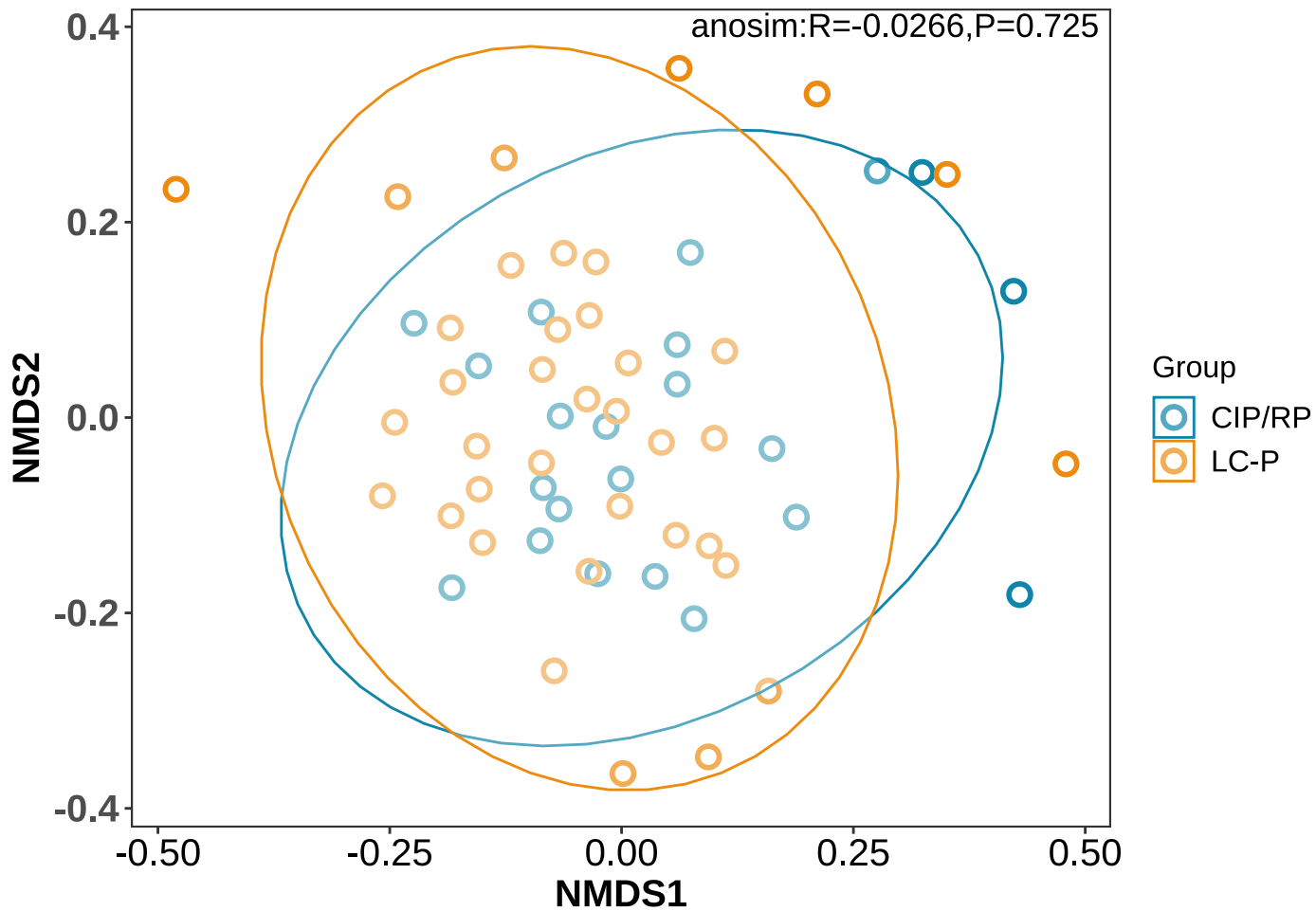

Supplement: Supplementary file 2 [file DataSheet1.zip › Data-all result/beta_div/Group_bray_nmds_beta_point.pdf]

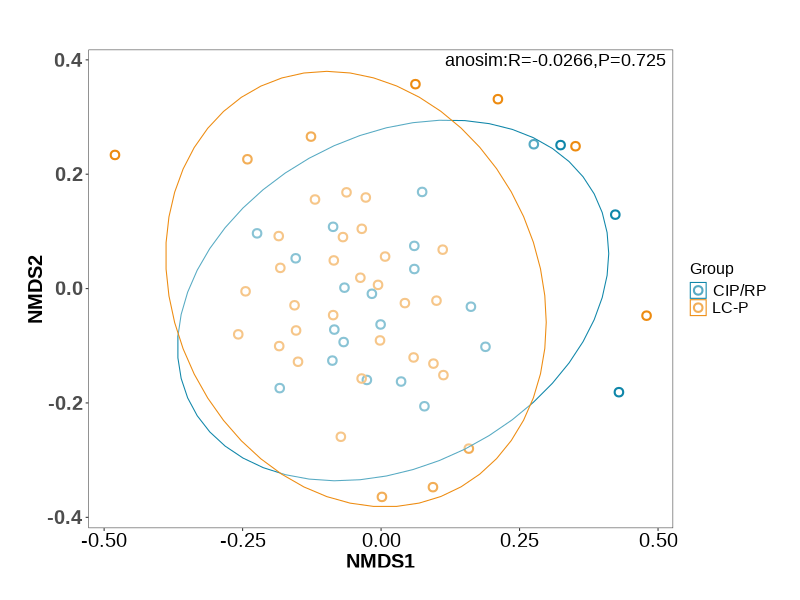

Supplement: Supplementary file 2 [file DataSheet1.zip › Data-all result/beta_div/Group_bray_nmds_beta_point.png]

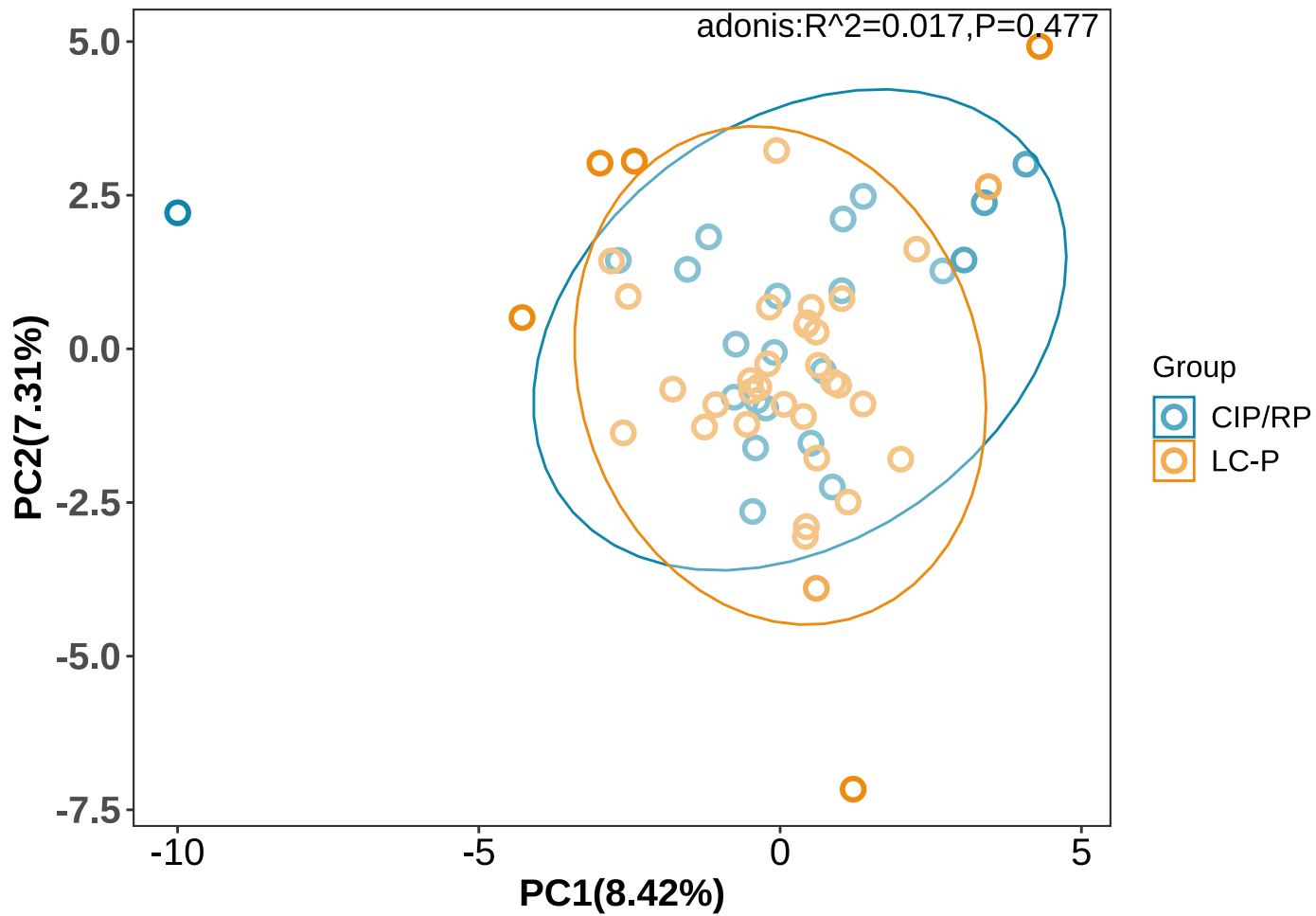

Supplement: Supplementary file 2 [file DataSheet1.zip › Data-all result/beta_div/Group_bray_pca_beta_point.pdf]

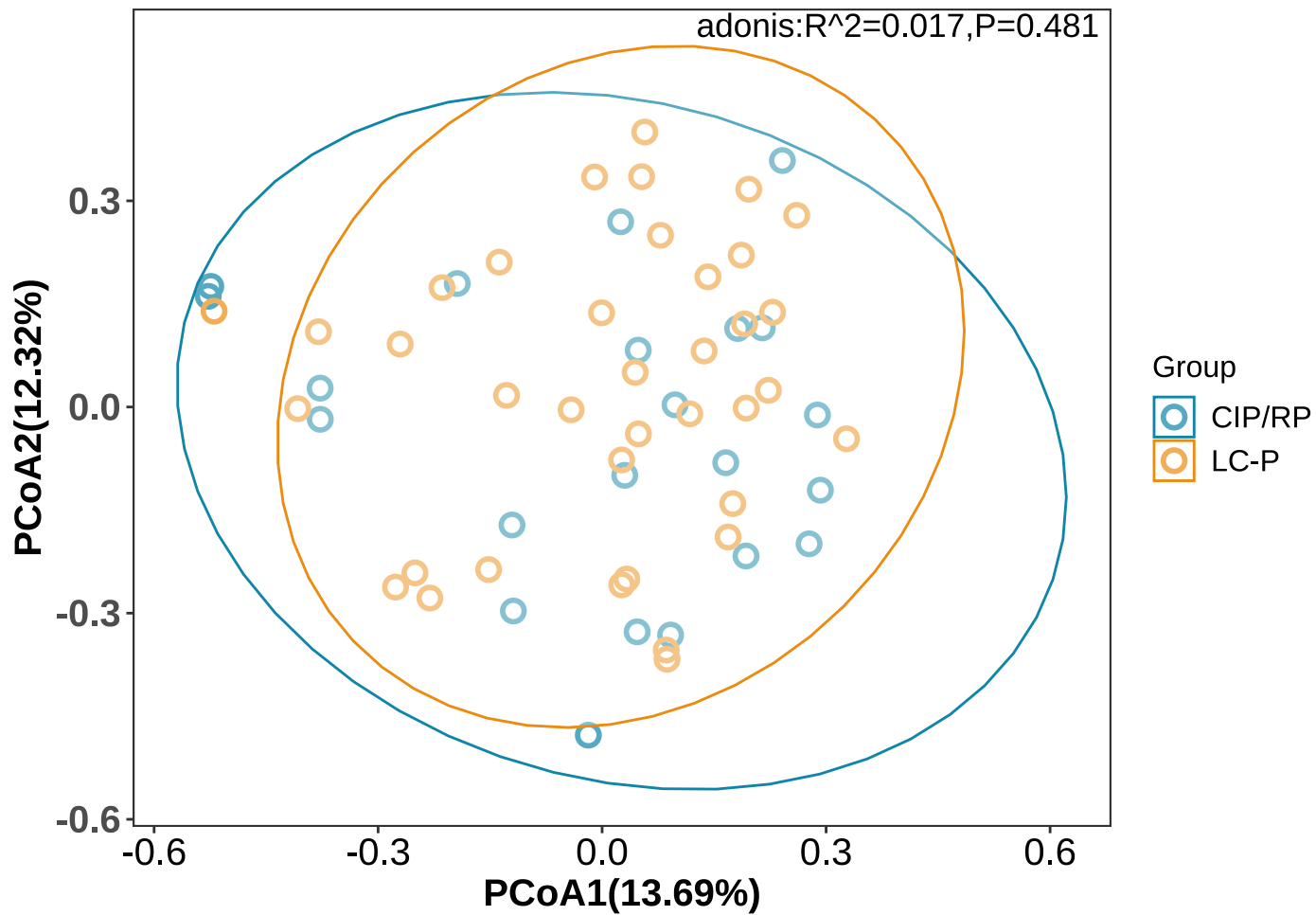

Supplement: Supplementary file 2 [file DataSheet1.zip › Data-all result/beta_div/Group_bray_pcoa_beta_point.pdf]

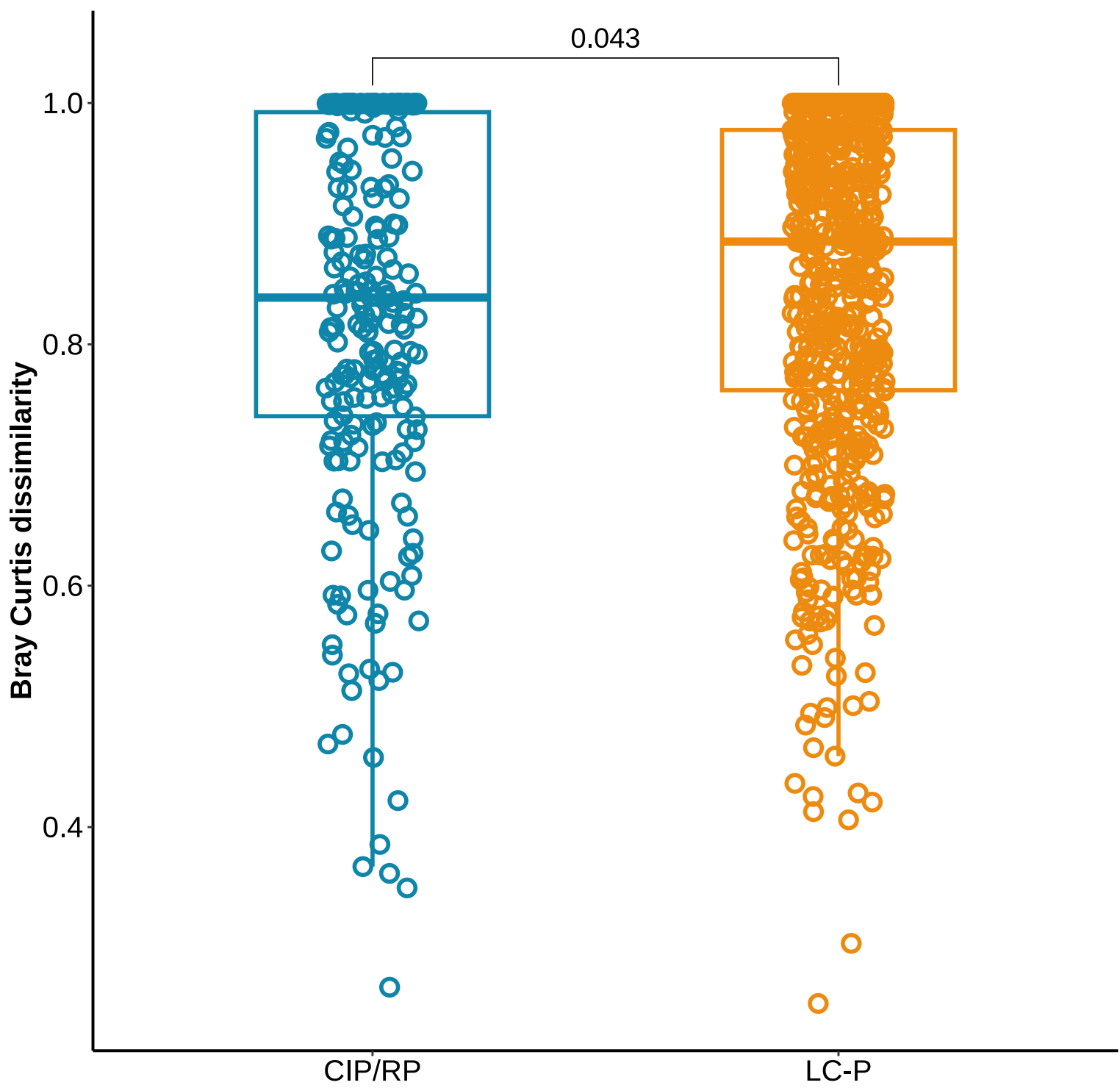

Supplement: Supplementary file 2 [file DataSheet1.zip › Data-all result/beta_div/Group_species_bray_distance.pdf]

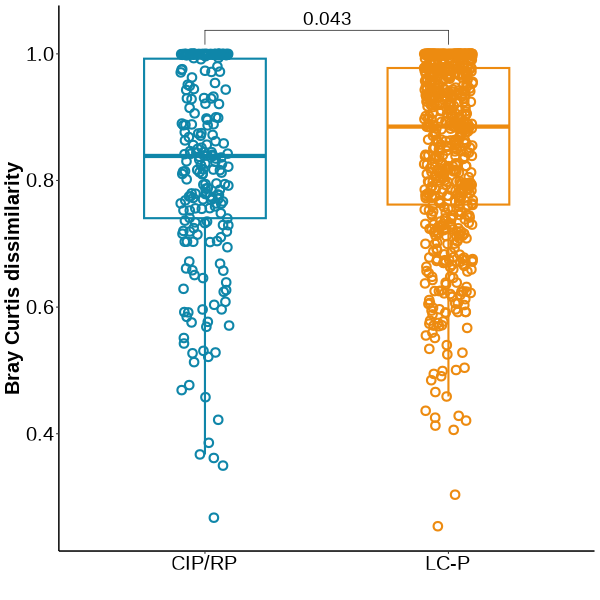

Supplement: Supplementary file 2 [file DataSheet1.zip › Data-all result/beta_div/Group_species_bray_distance.png]

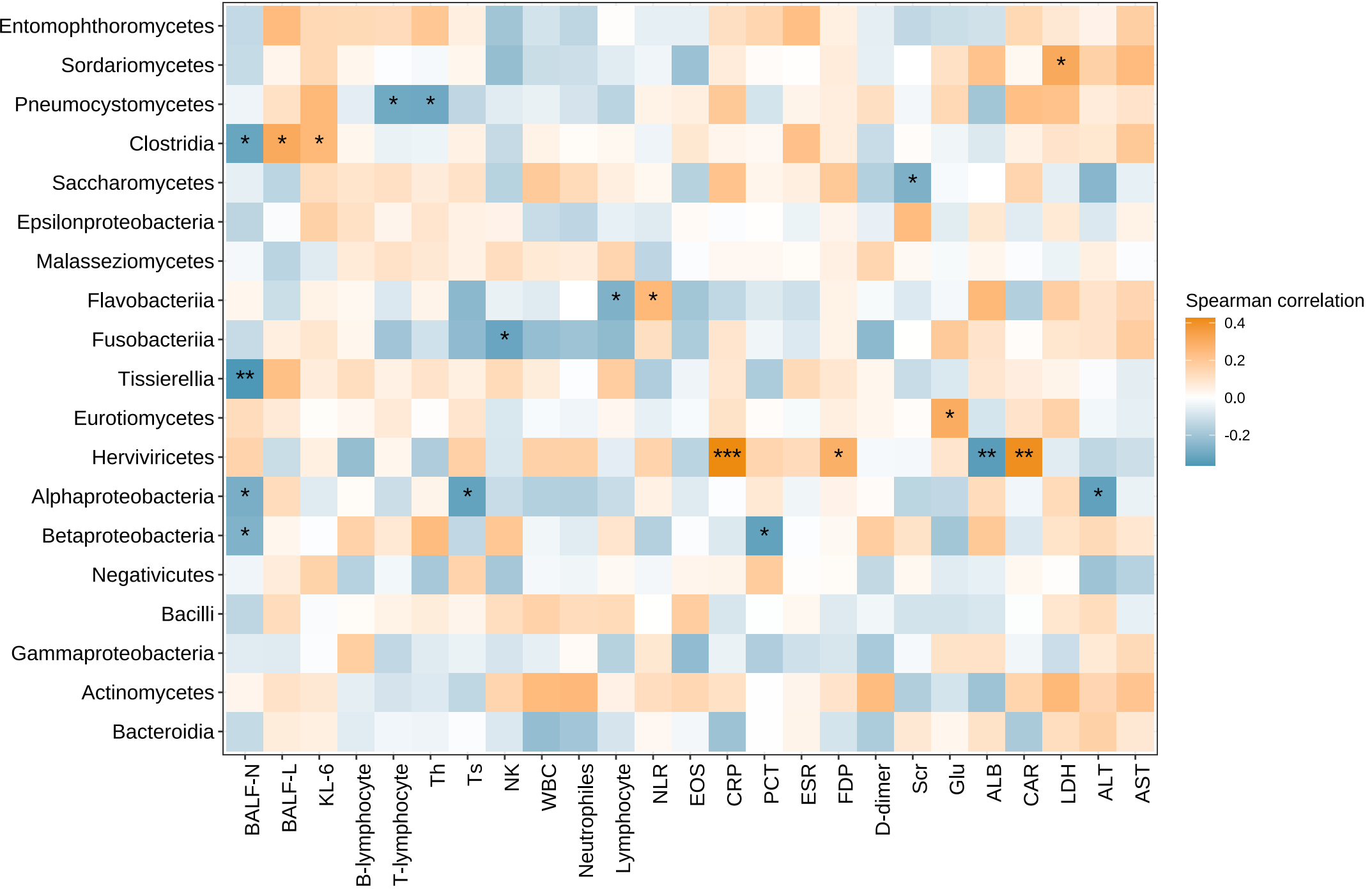

Supplement: Supplementary file 2 [file DataSheet1.zip › Data-all result/corr/Group_class_corr_heatmap.pdf]

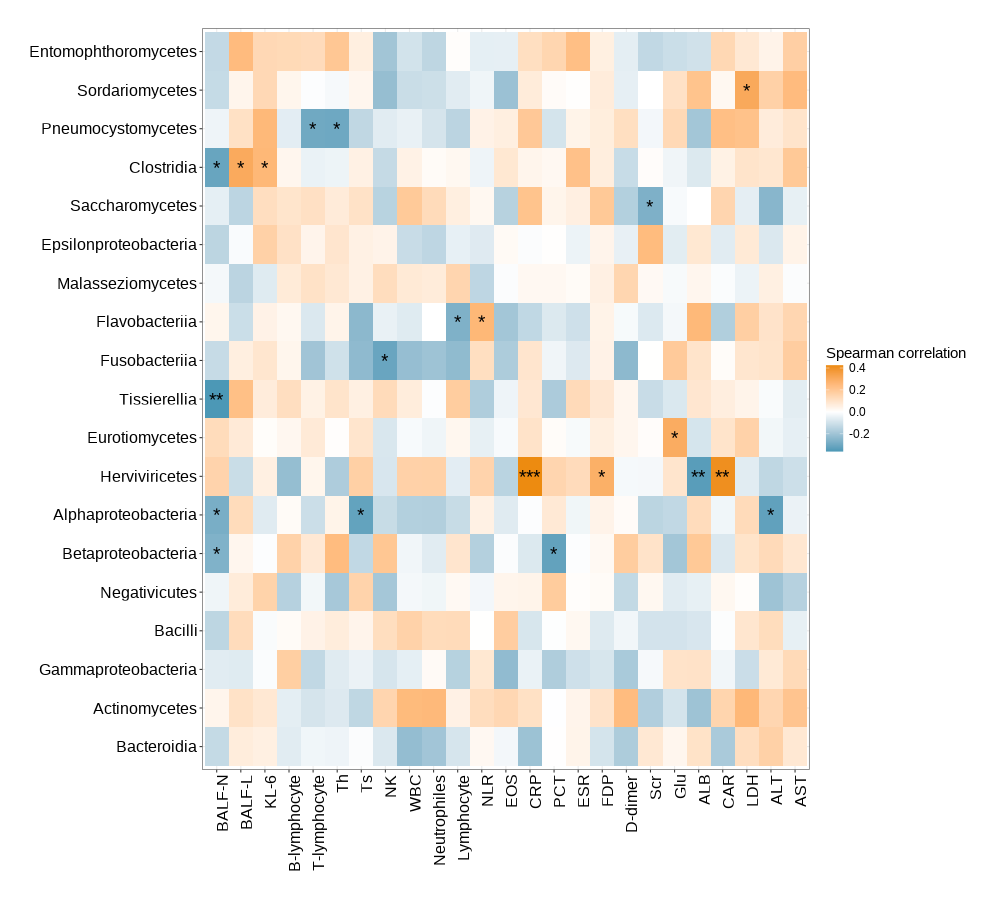

Supplement: Supplementary file 2 [file DataSheet1.zip › Data-all result/corr/Group_class_corr_heatmap.png]

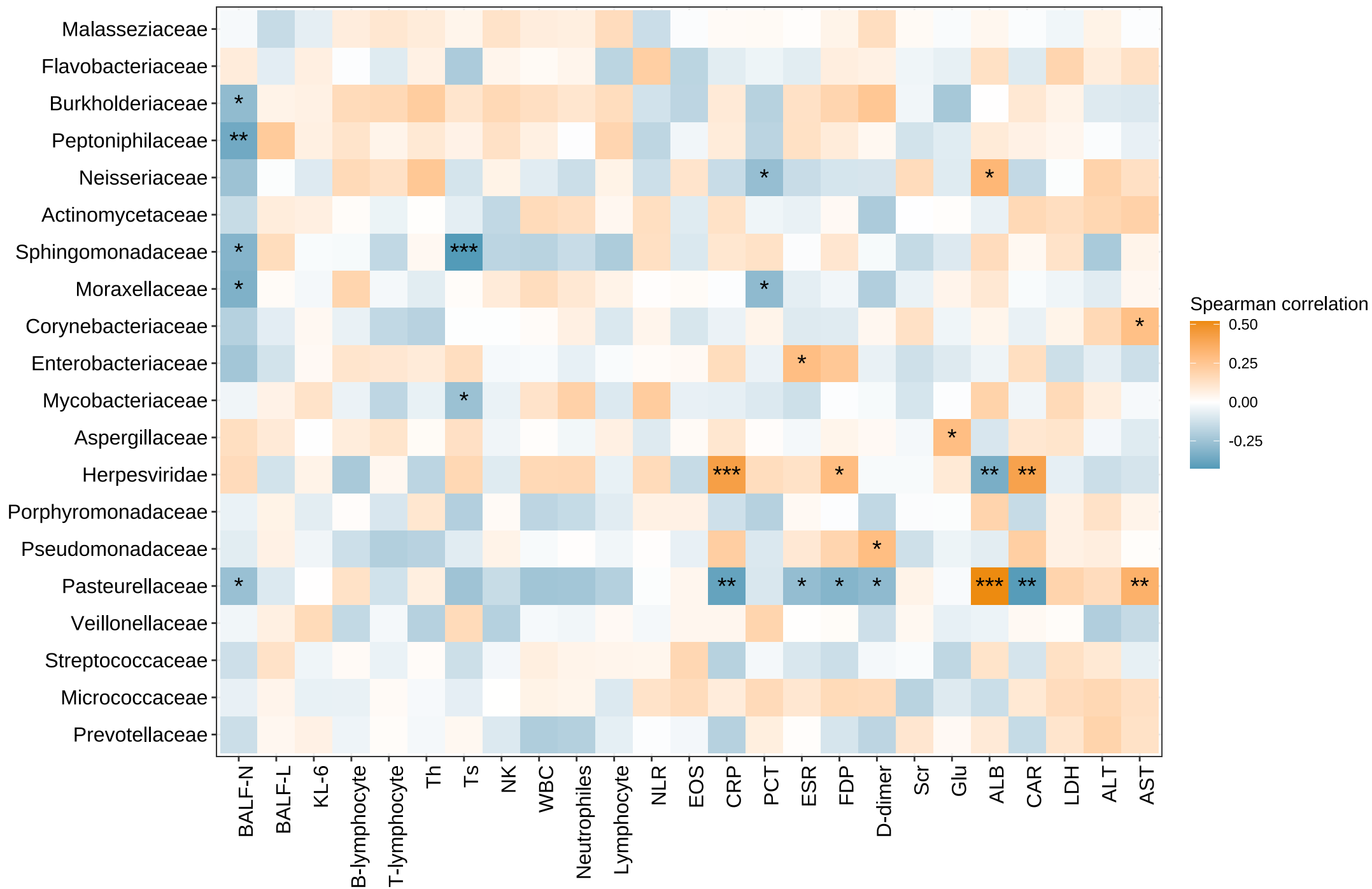

Supplement: Supplementary file 2 [file DataSheet1.zip › Data-all result/corr/Group_family_corr_heatmap.pdf]

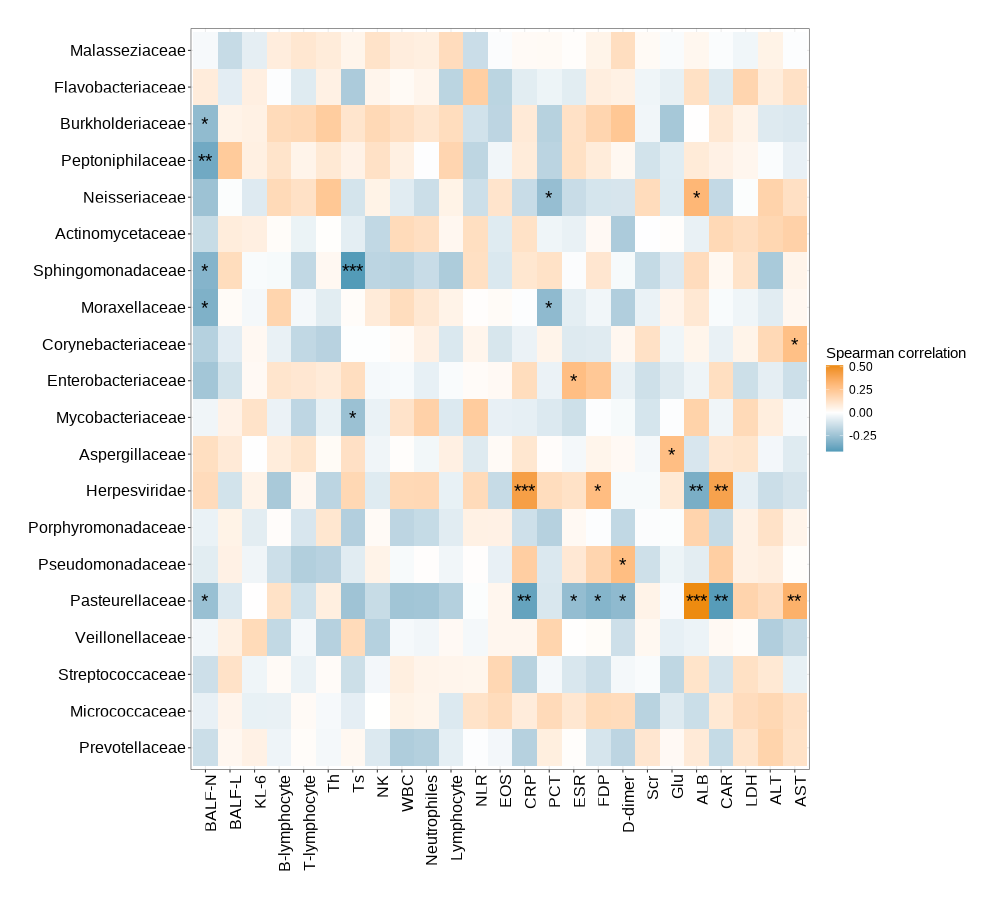

Supplement: Supplementary file 2 [file DataSheet1.zip › Data-all result/corr/Group_family_corr_heatmap.png]

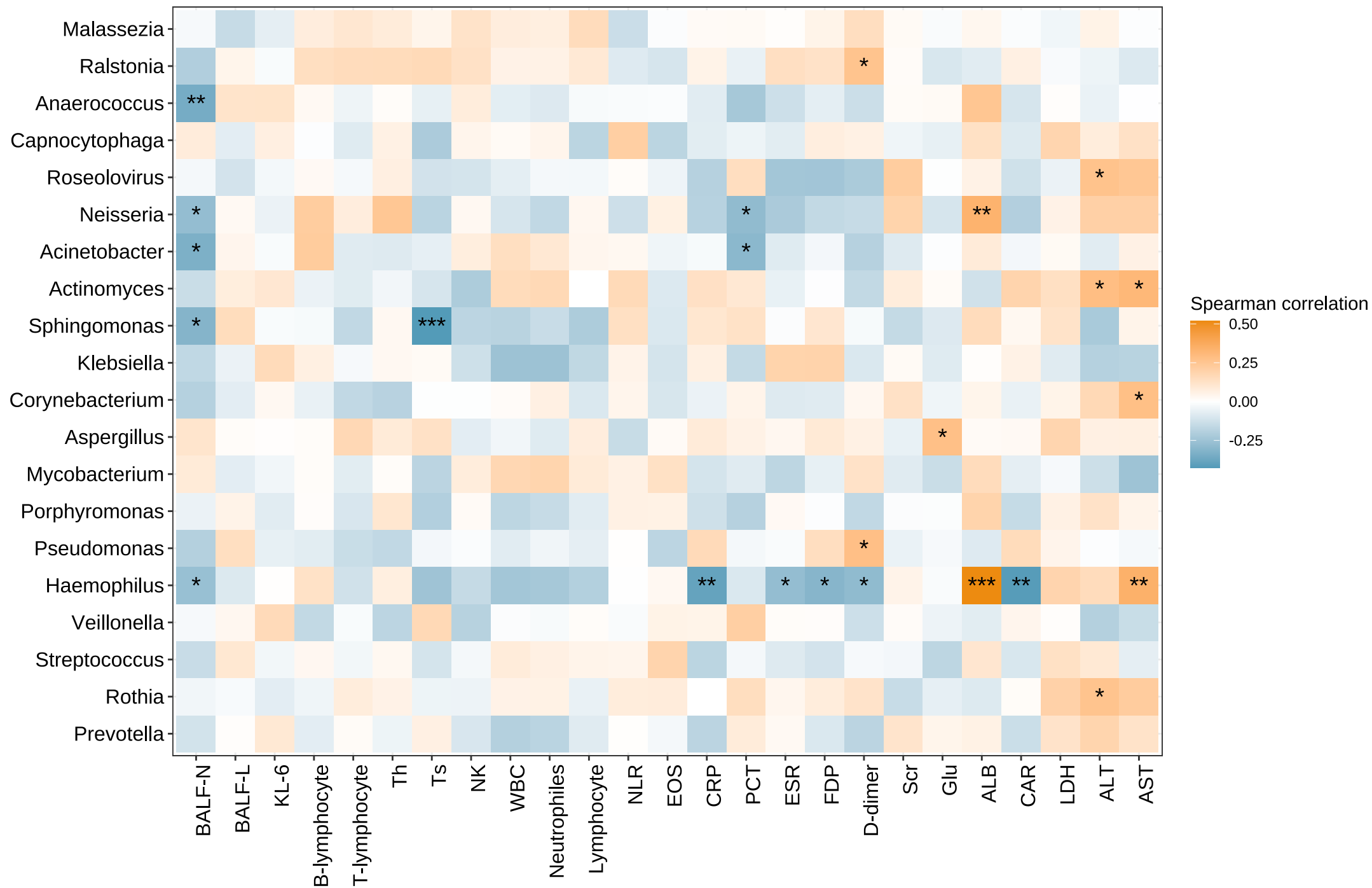

Supplement: Supplementary file 2 [file DataSheet1.zip › Data-all result/corr/Group_genus_corr_heatmap.pdf]

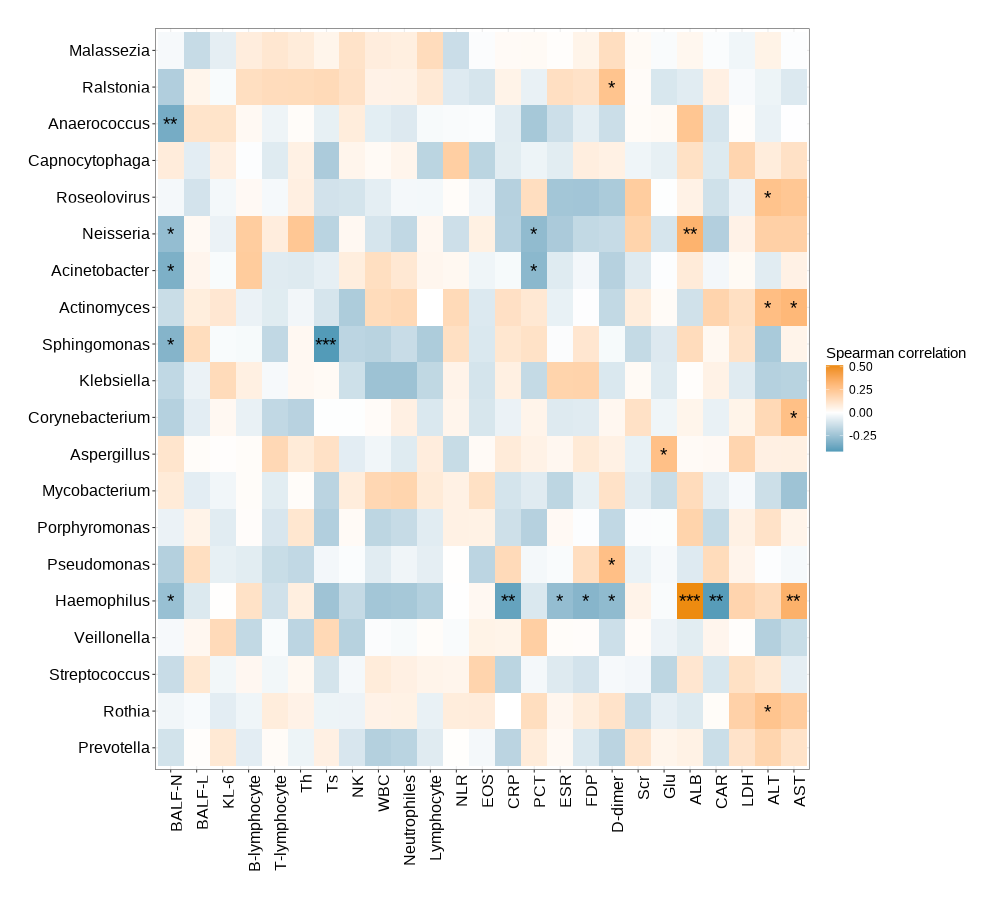

Supplement: Supplementary file 2 [file DataSheet1.zip › Data-all result/corr/Group_genus_corr_heatmap.png]

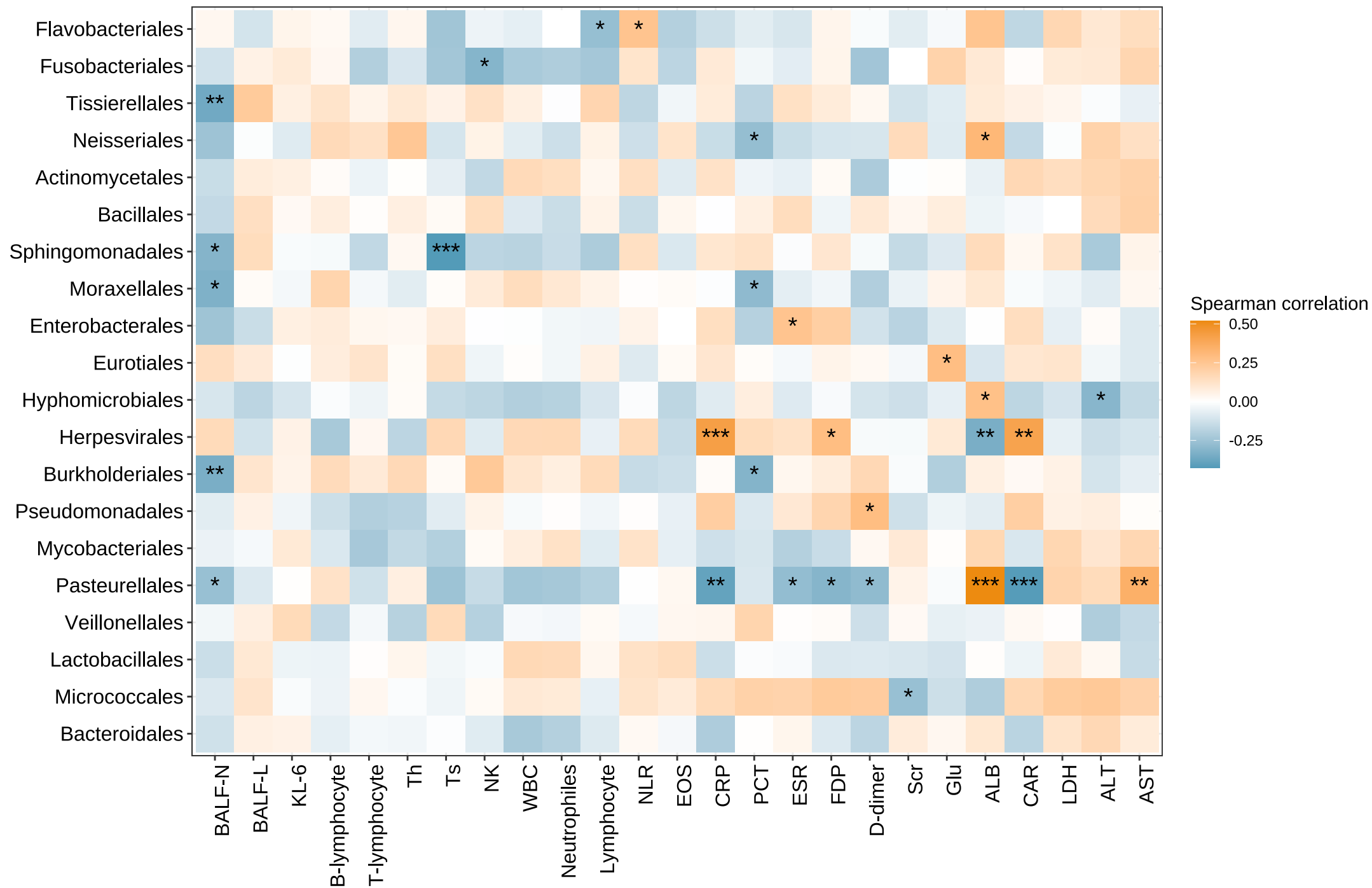

Supplement: Supplementary file 2 [file DataSheet1.zip › Data-all result/corr/Group_order_corr_heatmap.pdf]

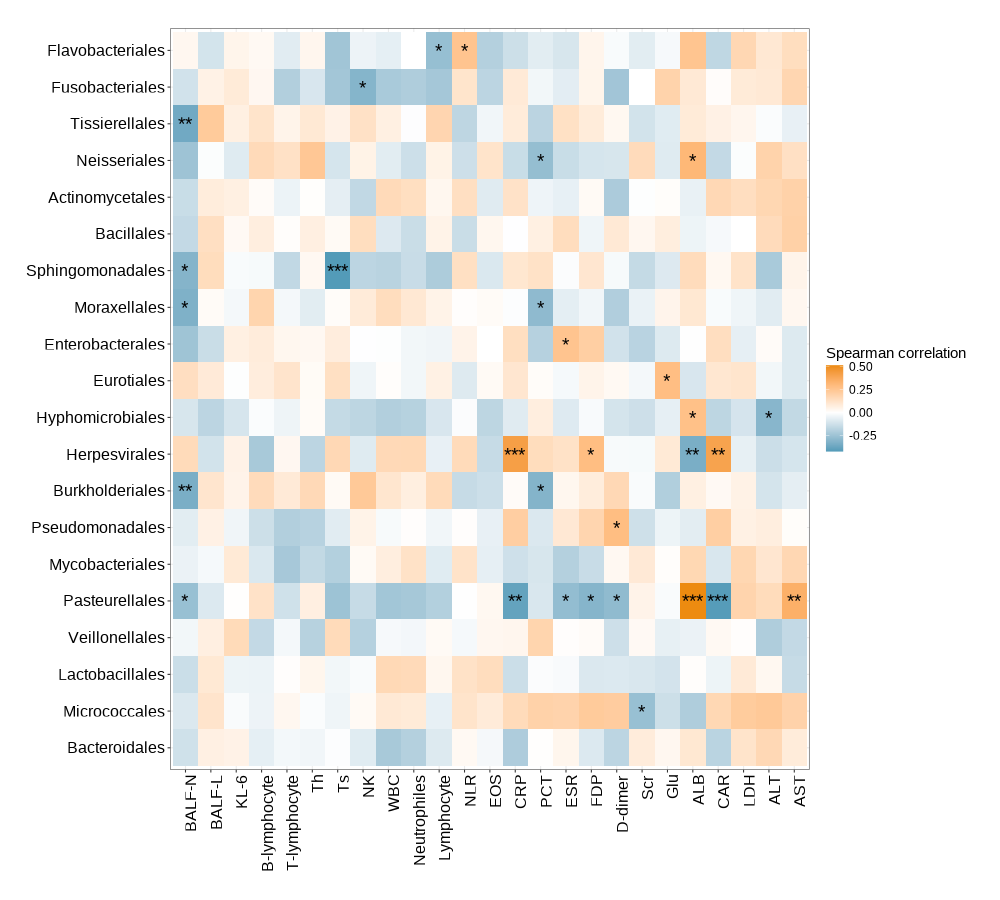

Supplement: Supplementary file 2 [file DataSheet1.zip › Data-all result/corr/Group_order_corr_heatmap.png]

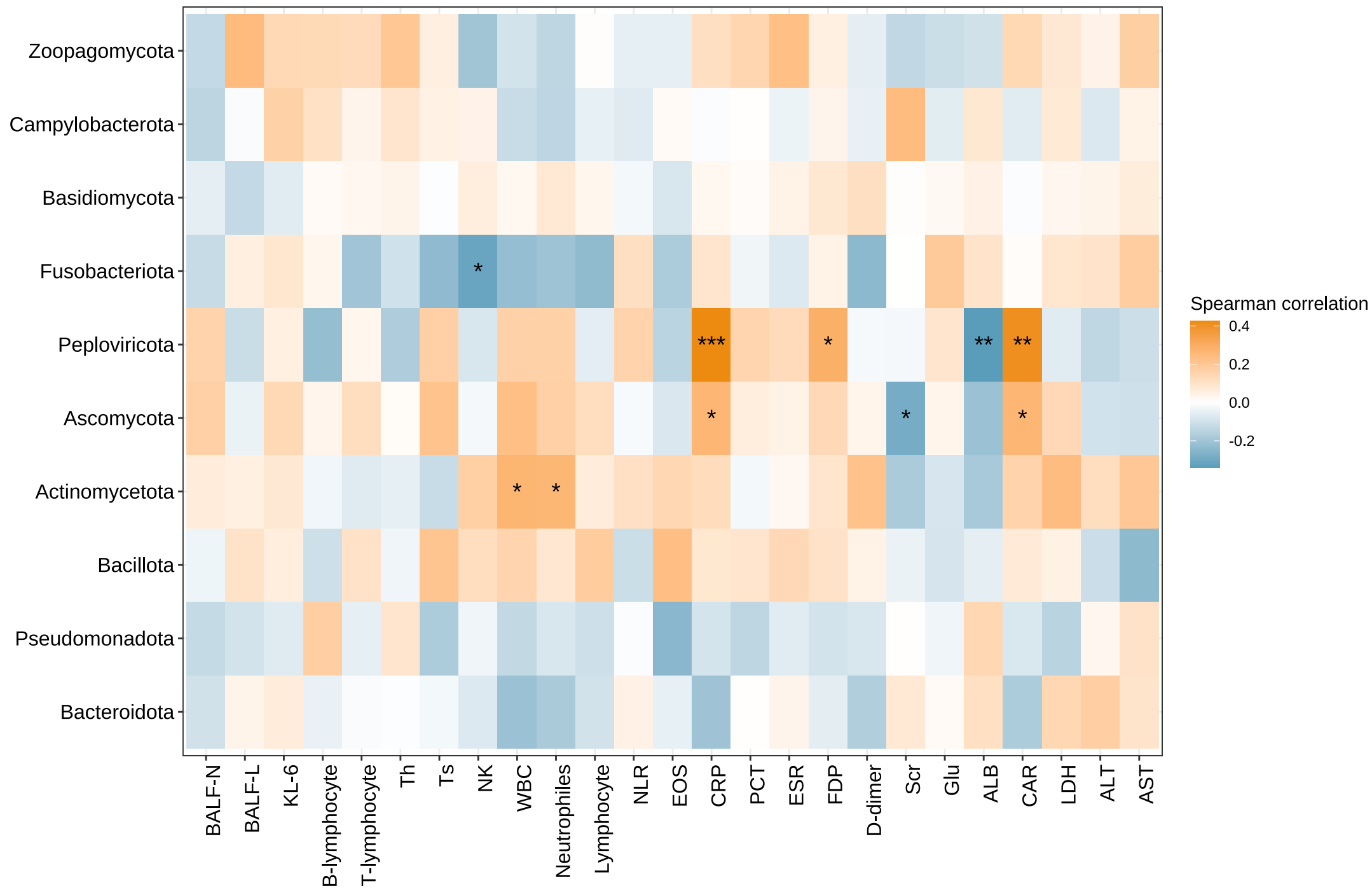

Supplement: Supplementary file 2 [file DataSheet1.zip › Data-all result/corr/Group_phylum_corr_heatmap.pdf]

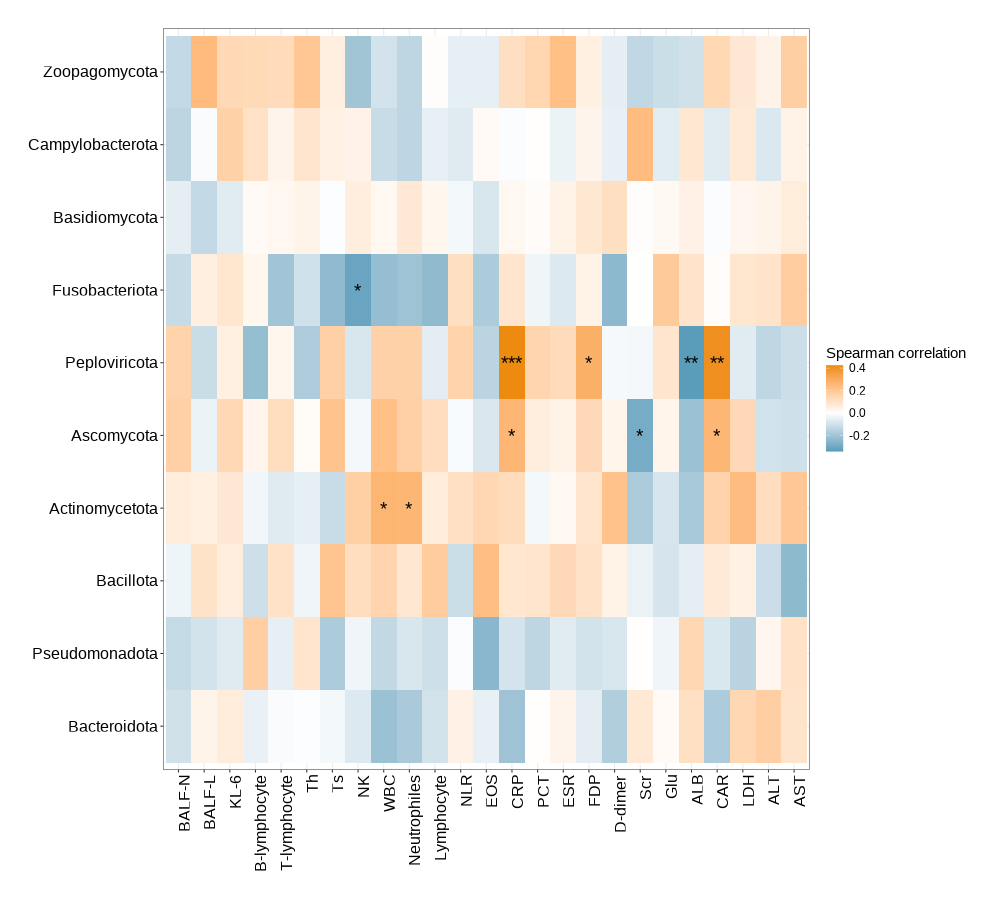

Supplement: Supplementary file 2 [file DataSheet1.zip › Data-all result/corr/Group_phylum_corr_heatmap.png]

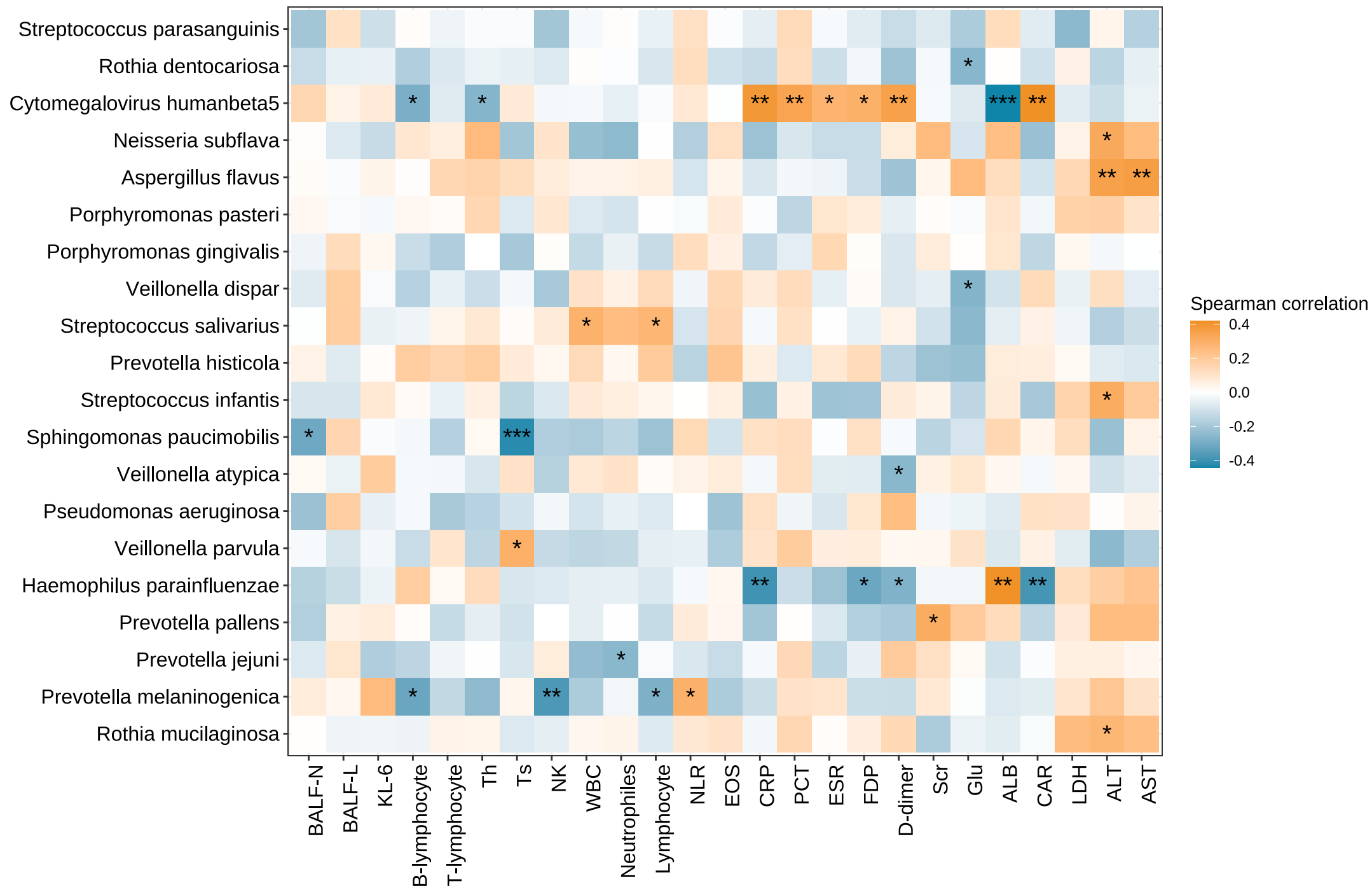

Supplement: Supplementary file 2 [file DataSheet1.zip › Data-all result/corr/Group_species_corr_heatmap.pdf]

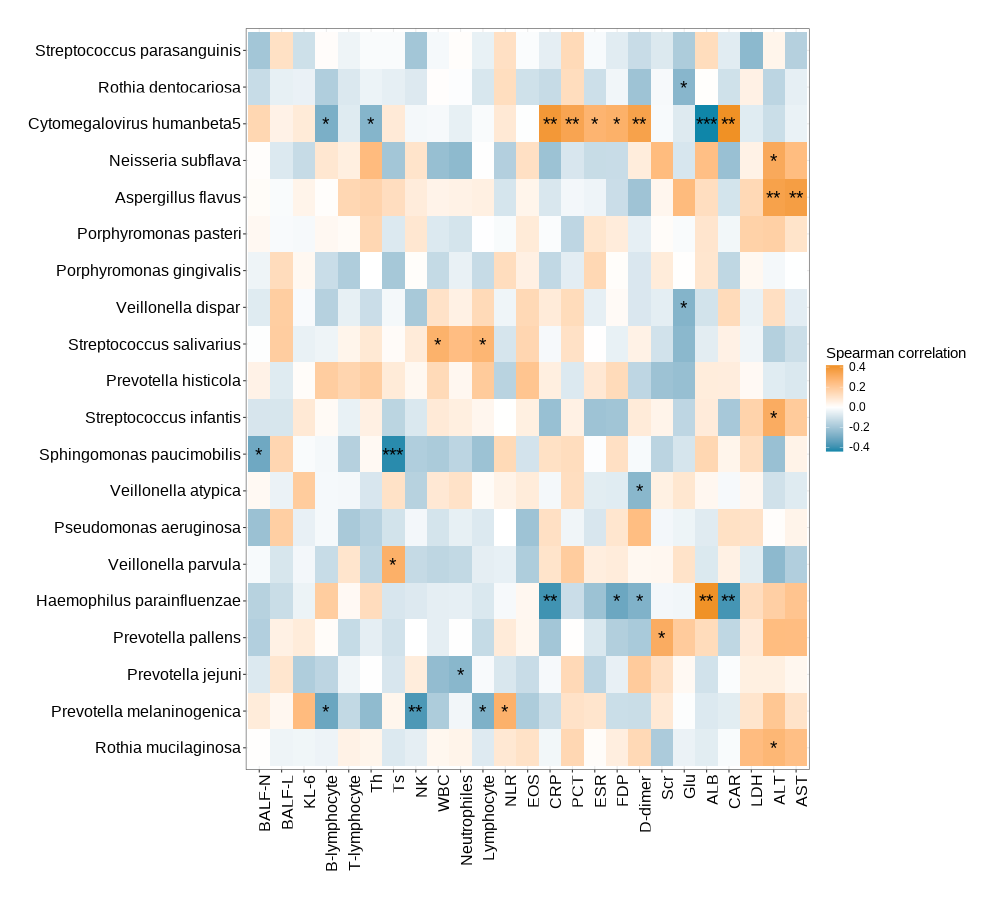

Supplement: Supplementary file 2 [file DataSheet1.zip › Data-all result/corr/Group_species_corr_heatmap.png]

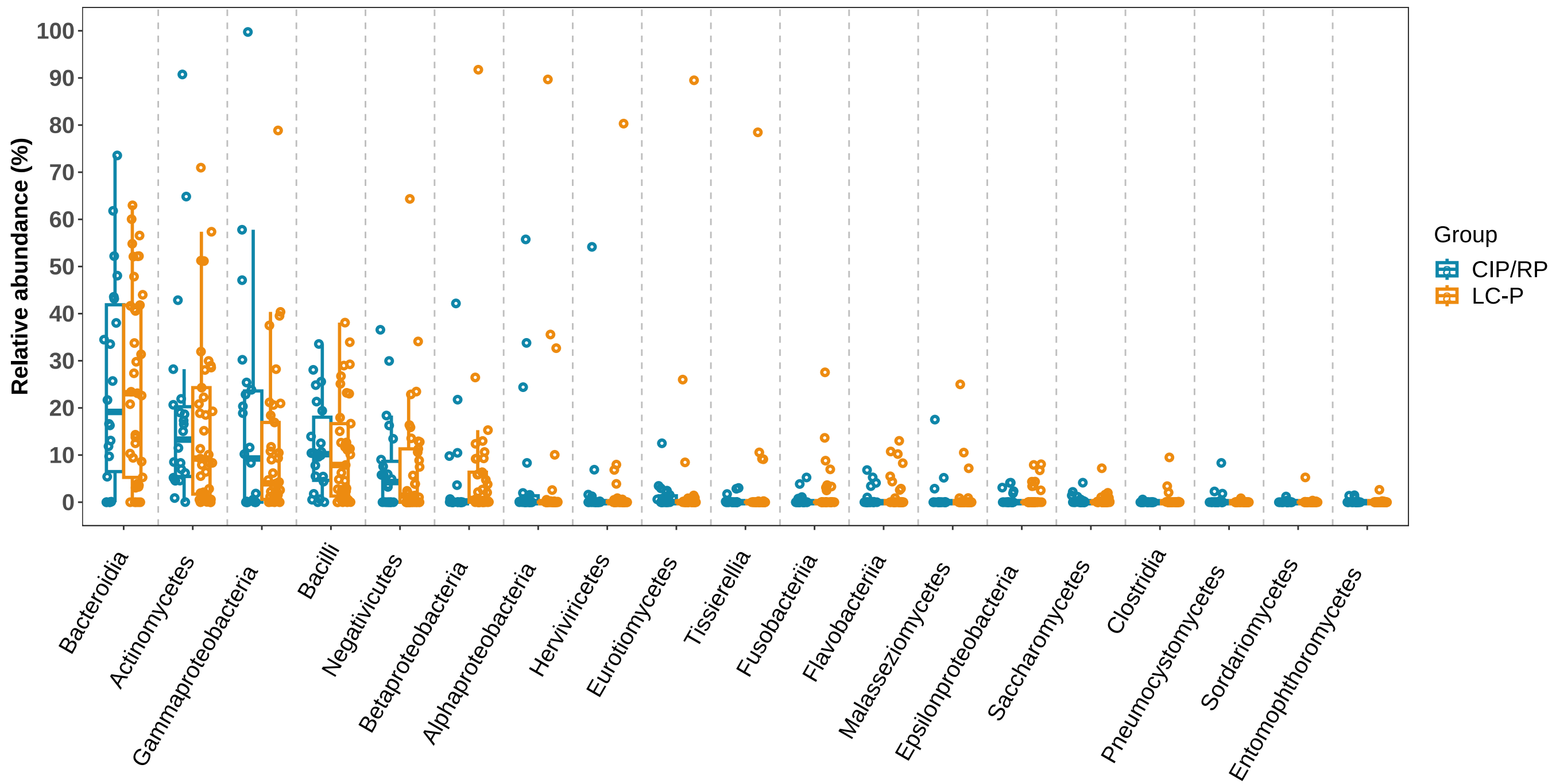

Supplement: Supplementary file 2 [file DataSheet1.zip › Data-all result/diff/Group_class_diff_boxplot.pdf]

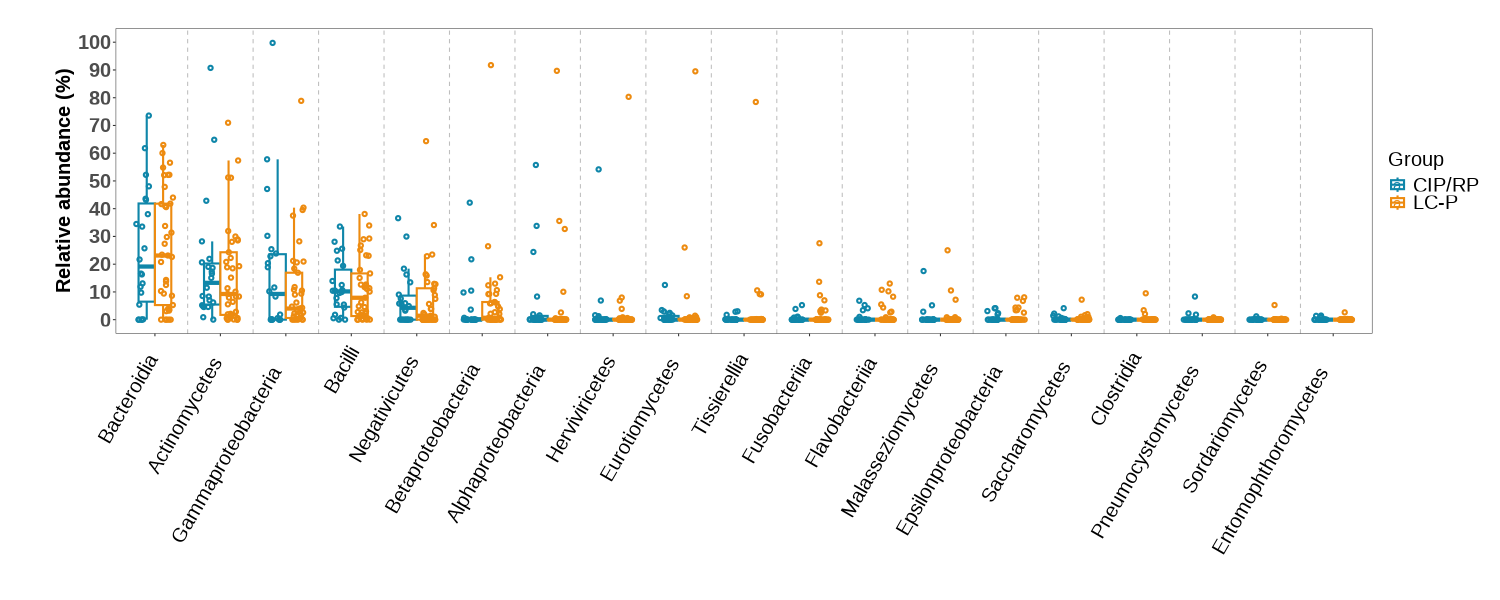

Supplement: Supplementary file 2 [file DataSheet1.zip › Data-all result/diff/Group_class_diff_boxplot.png]

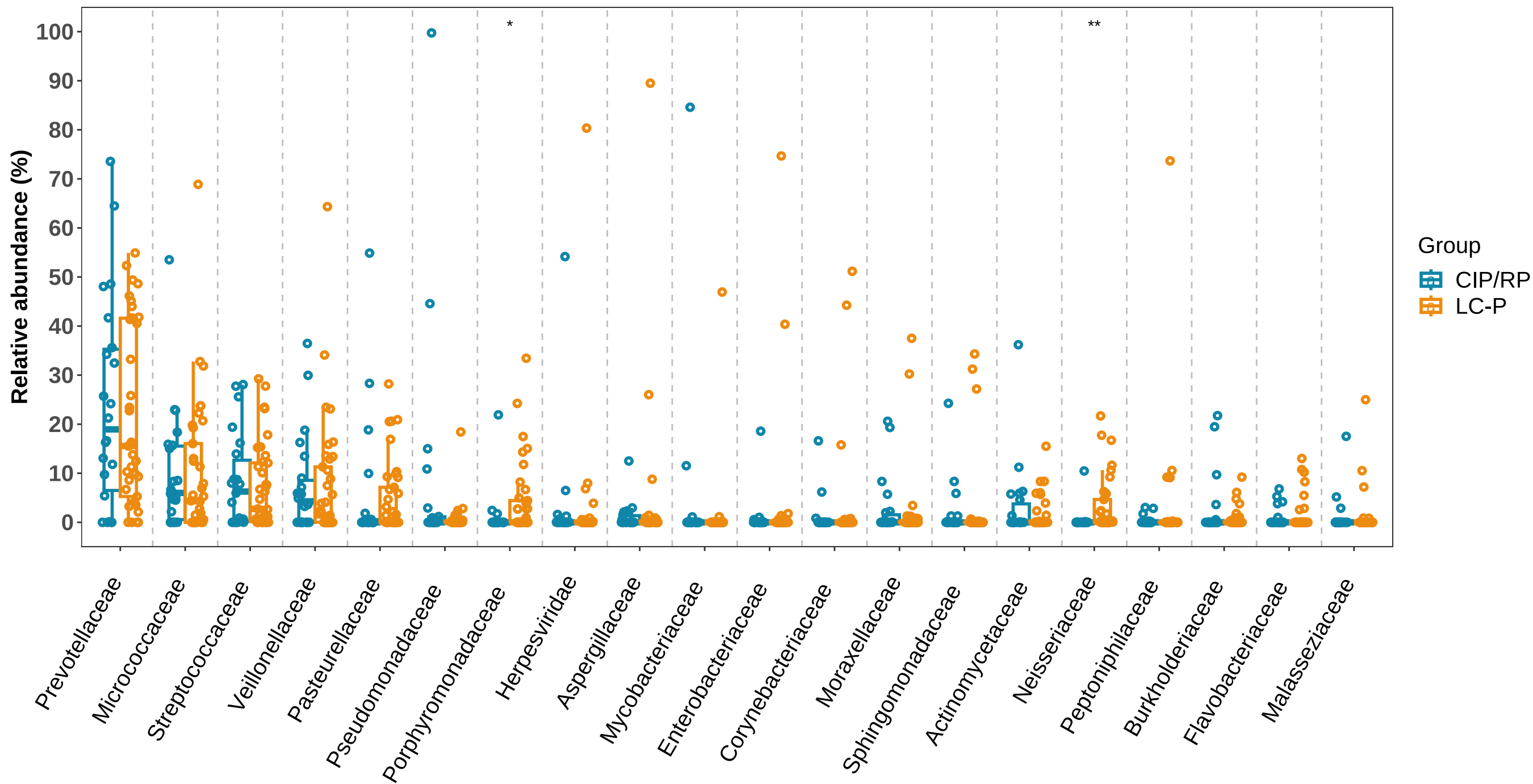

Supplement: Supplementary file 2 [file DataSheet1.zip › Data-all result/diff/Group_family_diff_boxplot.pdf]

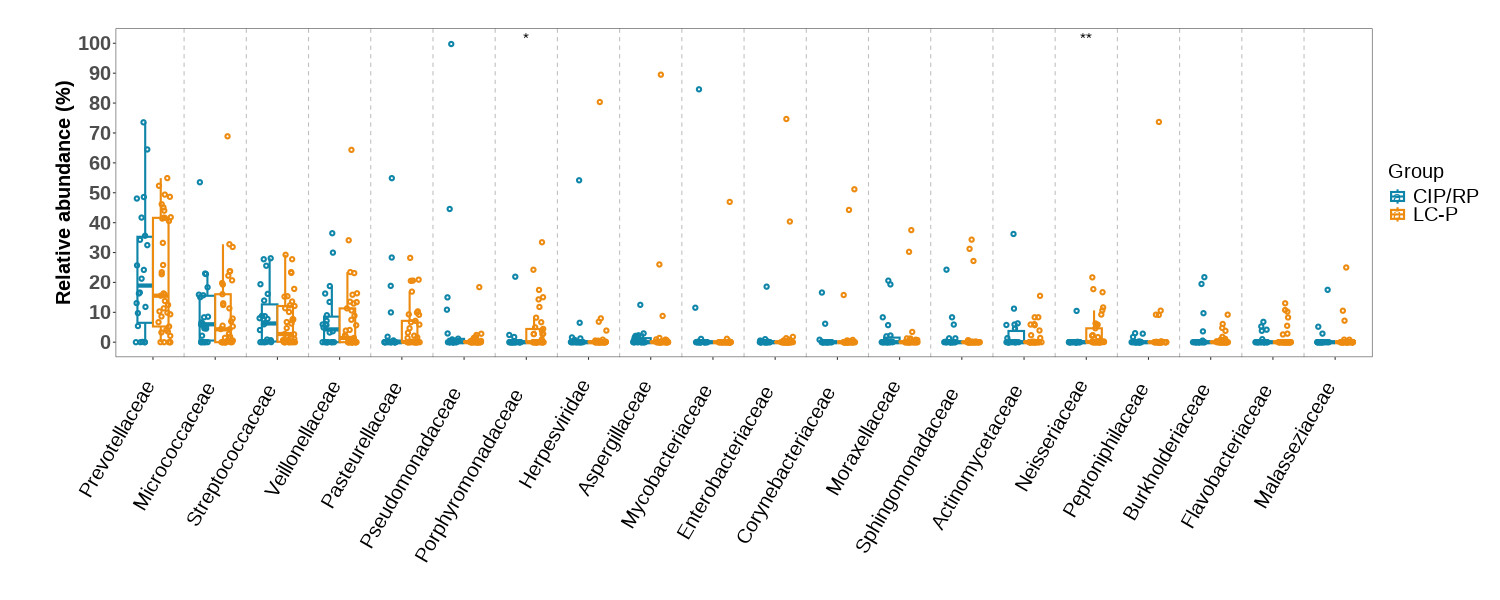

Supplement: Supplementary file 2 [file DataSheet1.zip › Data-all result/diff/Group_family_diff_boxplot.png]

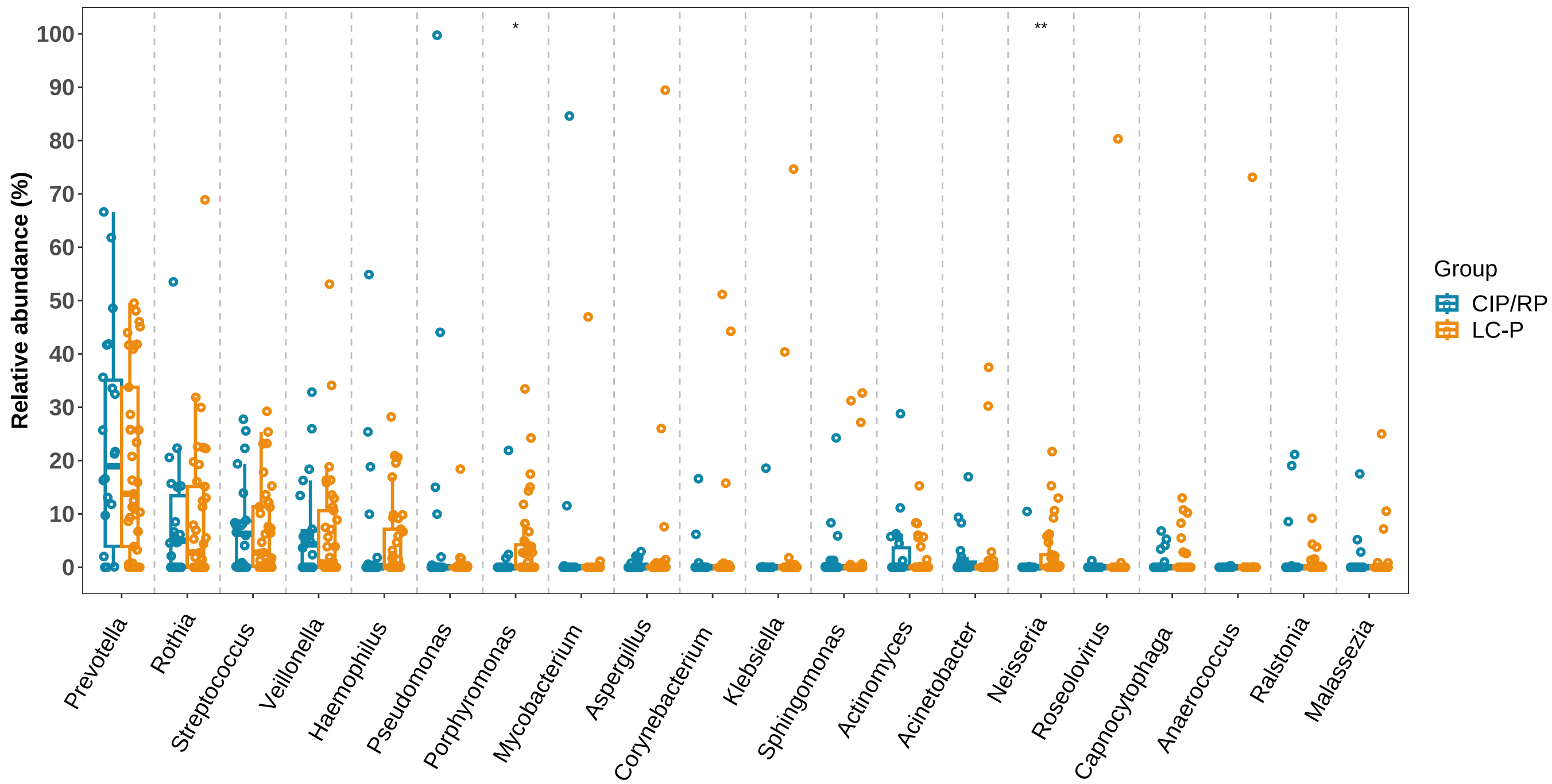

Supplement: Supplementary file 2 [file DataSheet1.zip › Data-all result/diff/Group_genus_diff_boxplot.pdf]

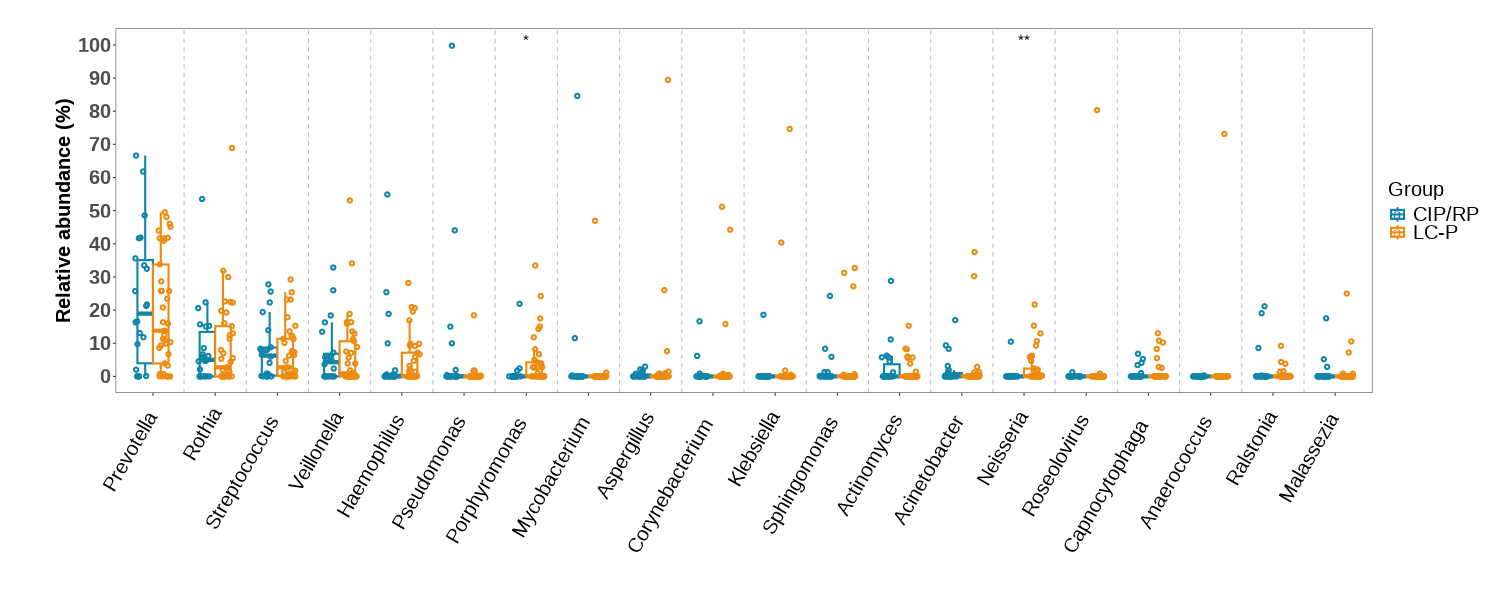

Supplement: Supplementary file 2 [file DataSheet1.zip › Data-all result/diff/Group_genus_diff_boxplot.png]

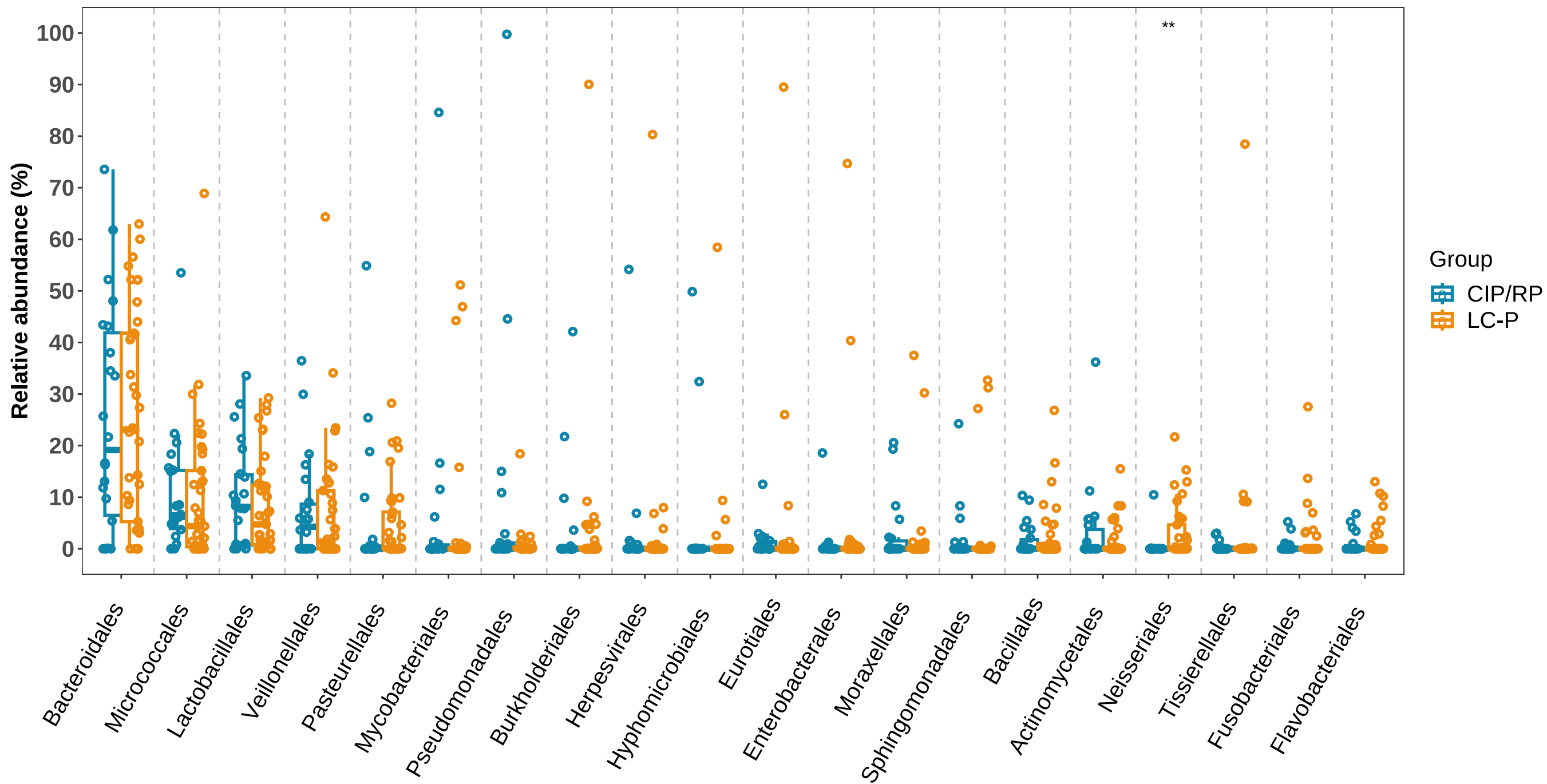

Supplement: Supplementary file 2 [file DataSheet1.zip › Data-all result/diff/Group_order_diff_boxplot.pdf]

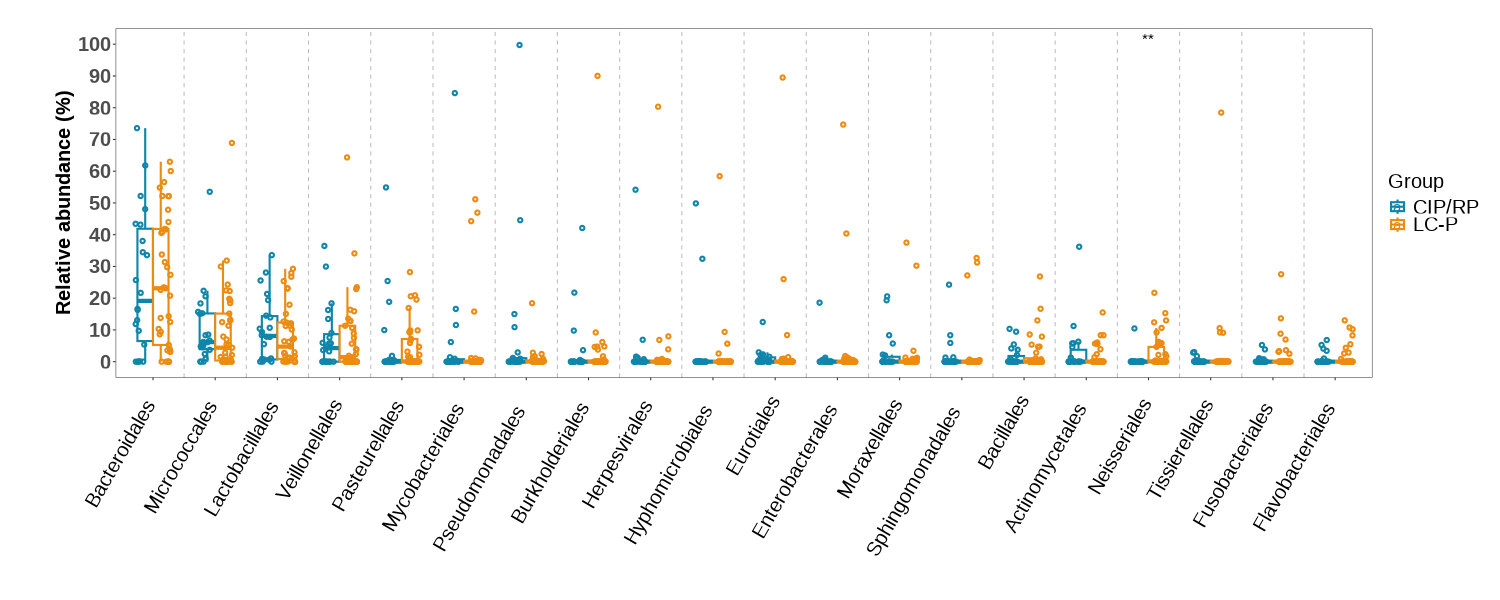

Supplement: Supplementary file 2 [file DataSheet1.zip › Data-all result/diff/Group_order_diff_boxplot.png]

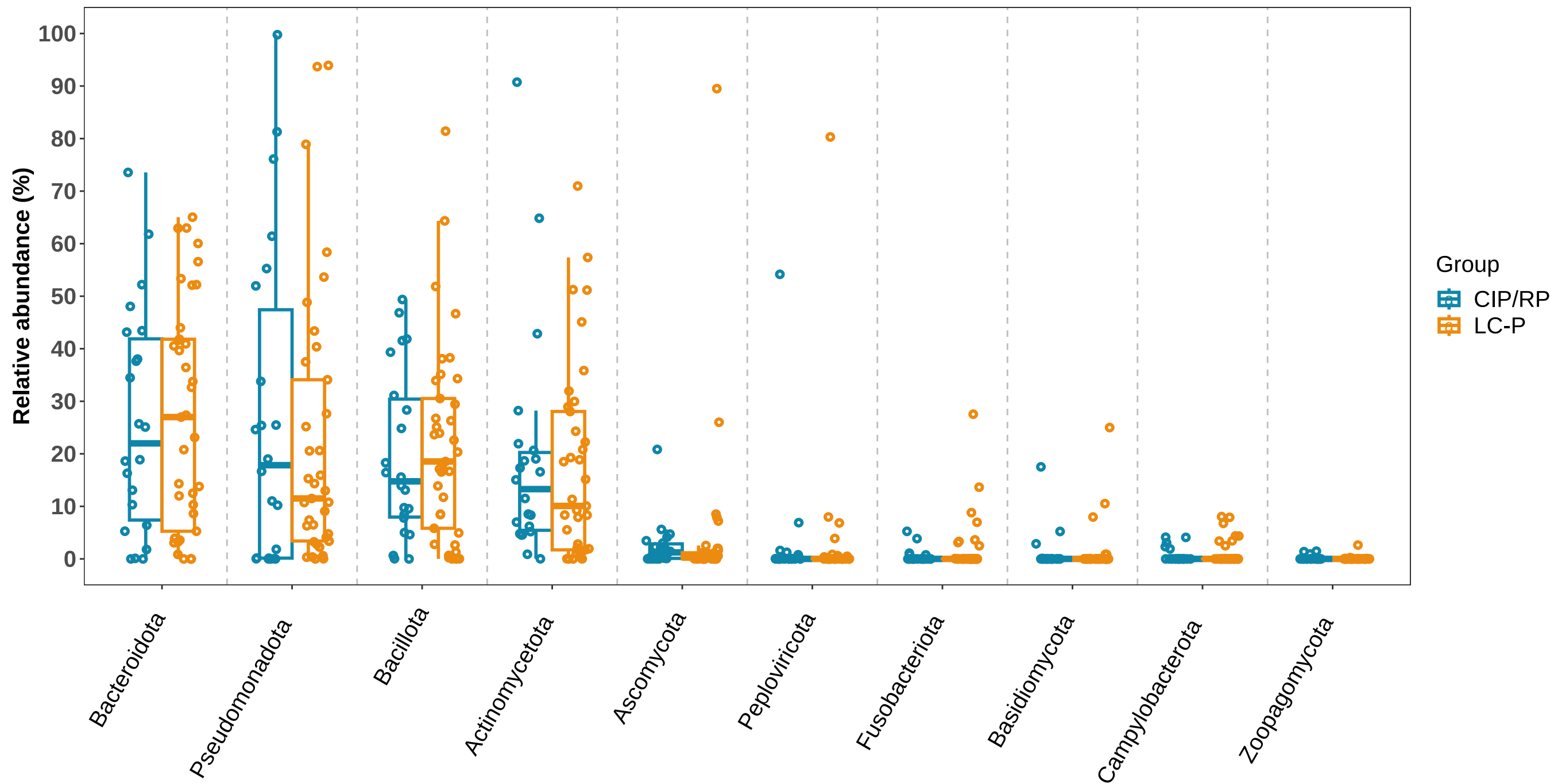

Supplement: Supplementary file 2 [file DataSheet1.zip › Data-all result/diff/Group_phylum_diff_boxplot.pdf]

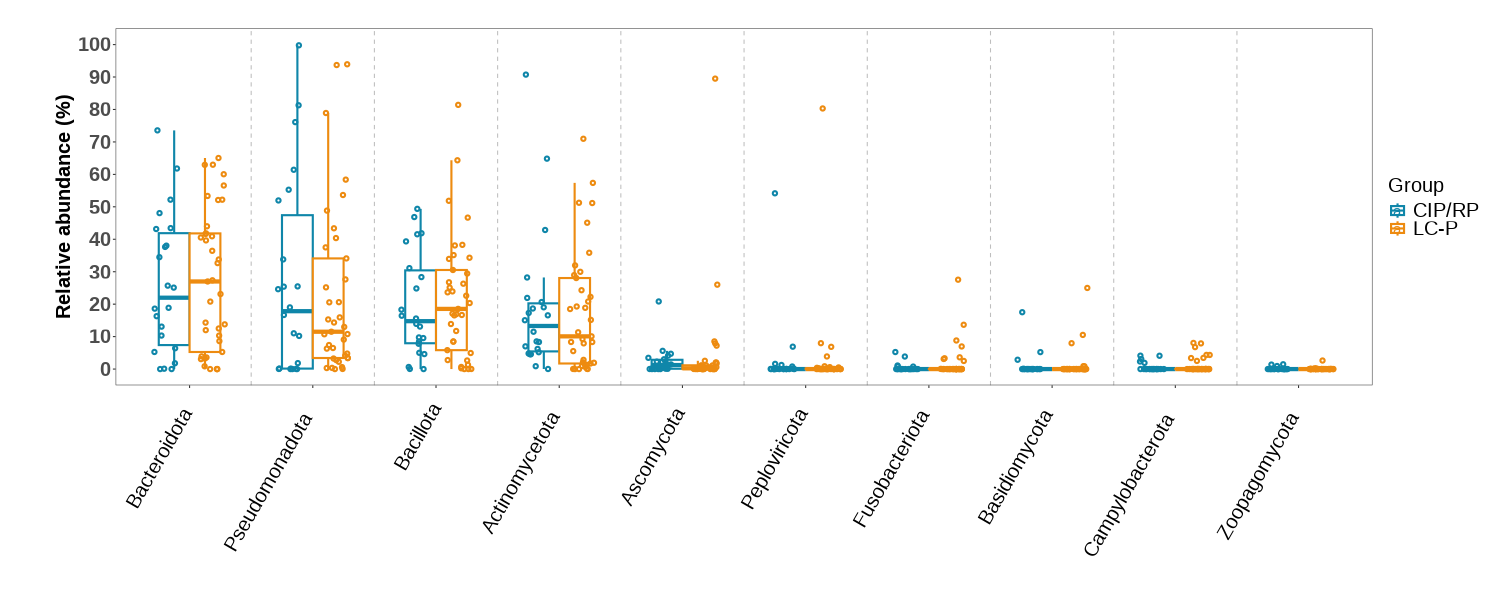

Supplement: Supplementary file 2 [file DataSheet1.zip › Data-all result/diff/Group_phylum_diff_boxplot.png]

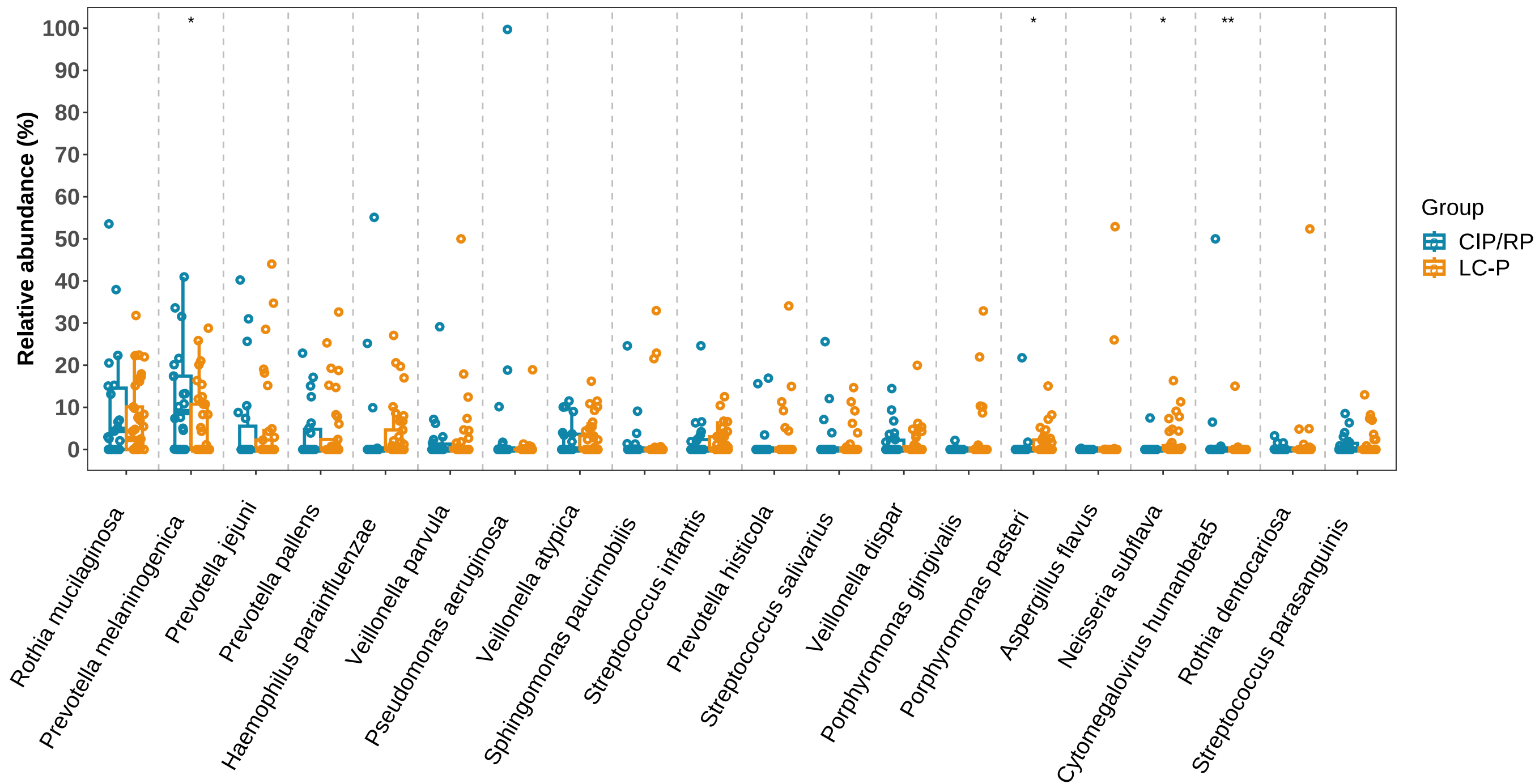

Supplement: Supplementary file 2 [file DataSheet1.zip › Data-all result/diff/Group_species_diff_boxplot.pdf]

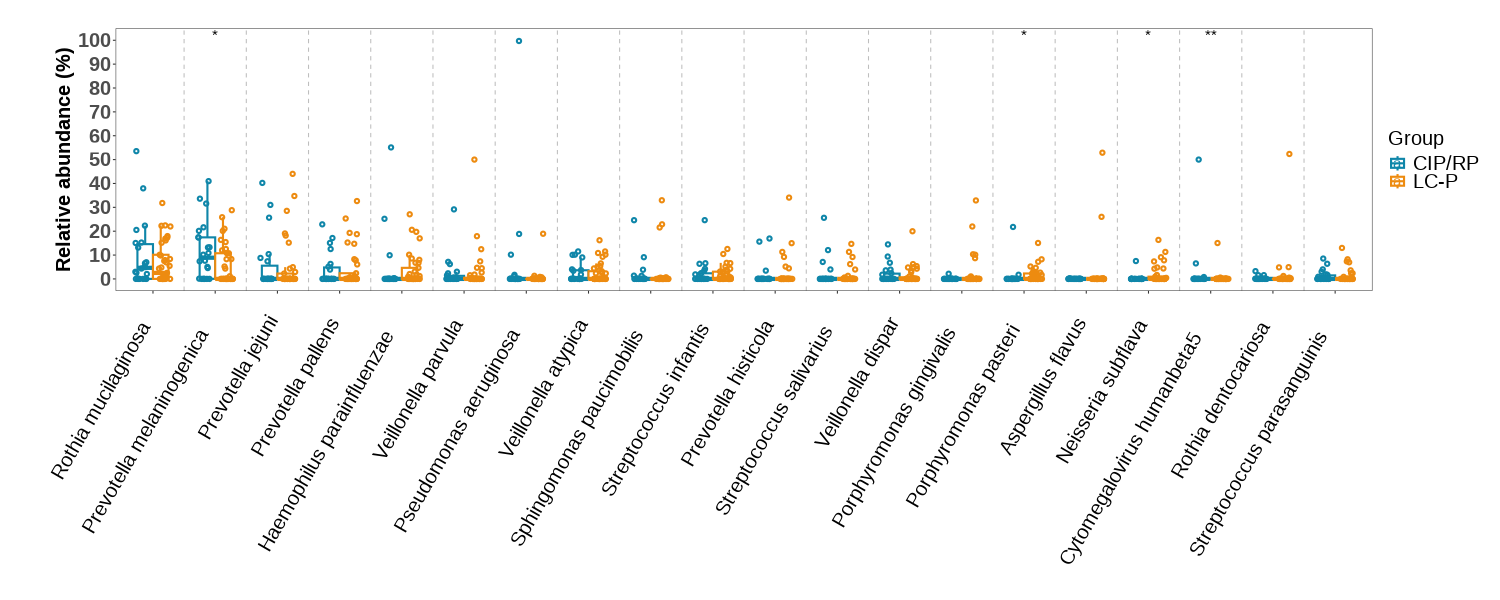

Supplement: Supplementary file 2 [file DataSheet1.zip › Data-all result/diff/Group_species_diff_boxplot.png]

■ CIP/RP    ■ LC-P

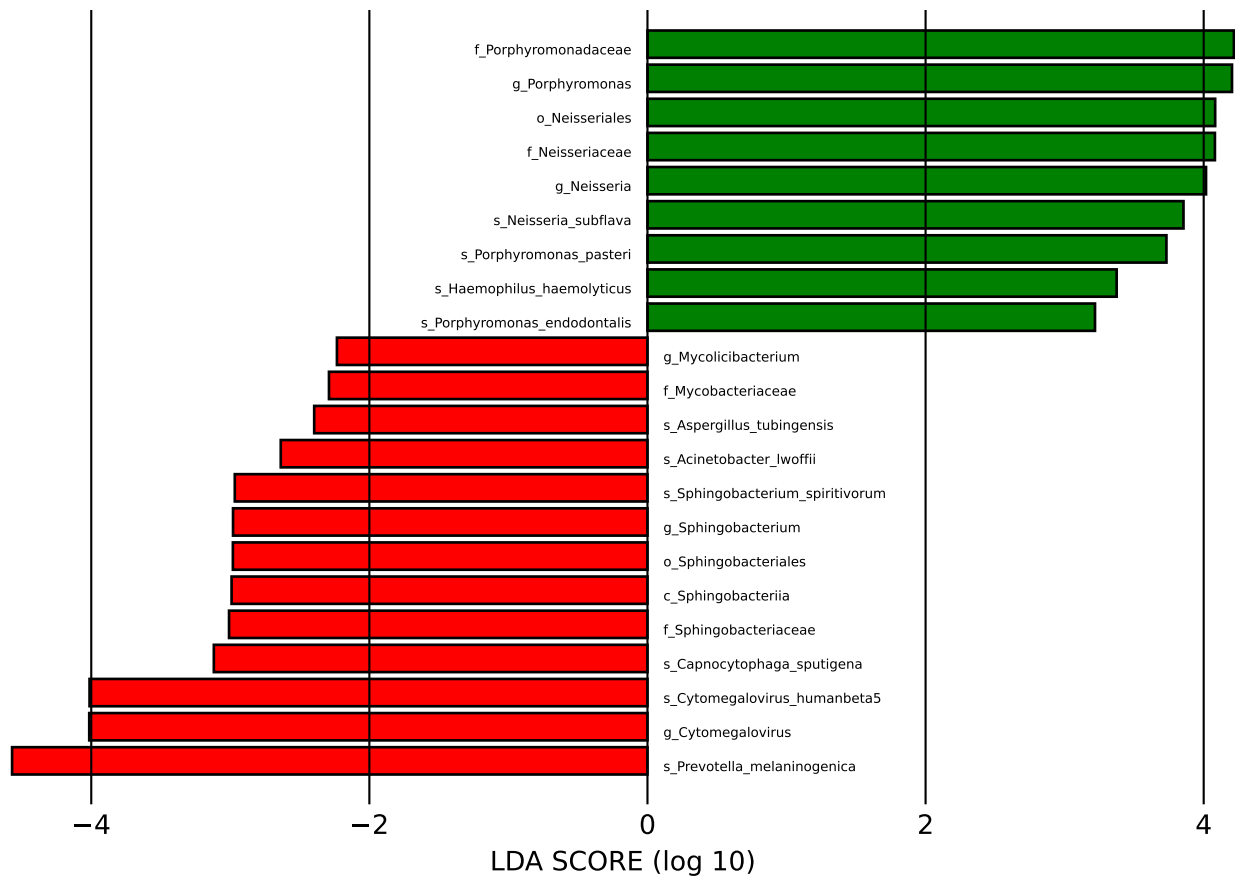

Supplement: Supplementary file 2 [file DataSheet1.zip › Data-all result/lefse/sample_lefse.Plot.pdf]

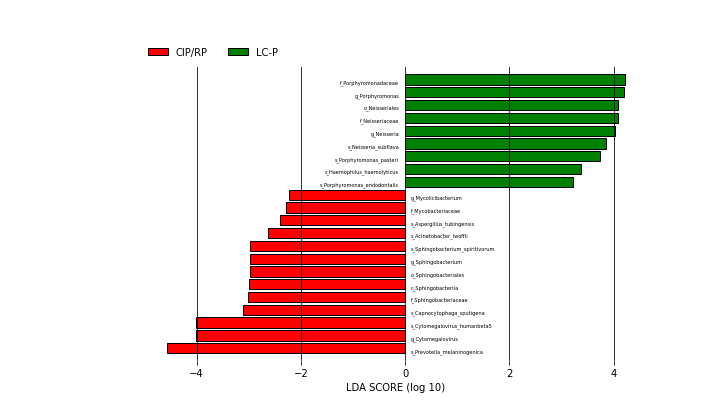

Supplement: Supplementary file 2 [file DataSheet1.zip › Data-all result/lefse/sample_lefse.Plot.png]

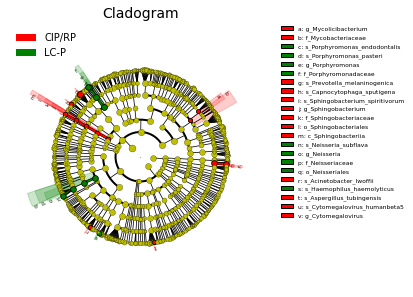

Supplement: Supplementary file 2 [file DataSheet1.zip › Data-all result/lefse/sample_lefse.Plot_Cladogram.png]

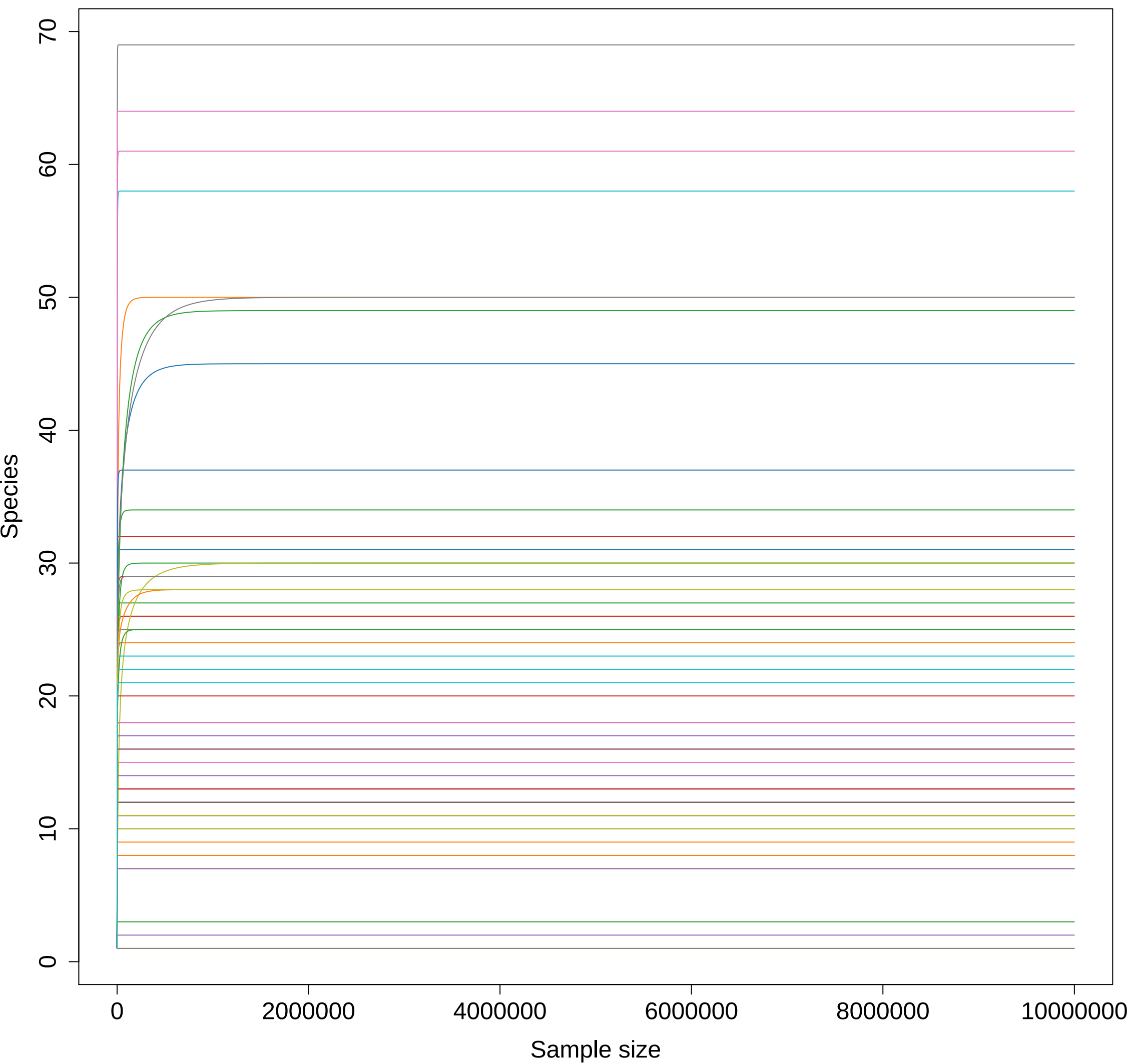

Supplement: Supplementary file 2 [file DataSheet1.zip › Data-all result/RarefactionCurve.pdf]

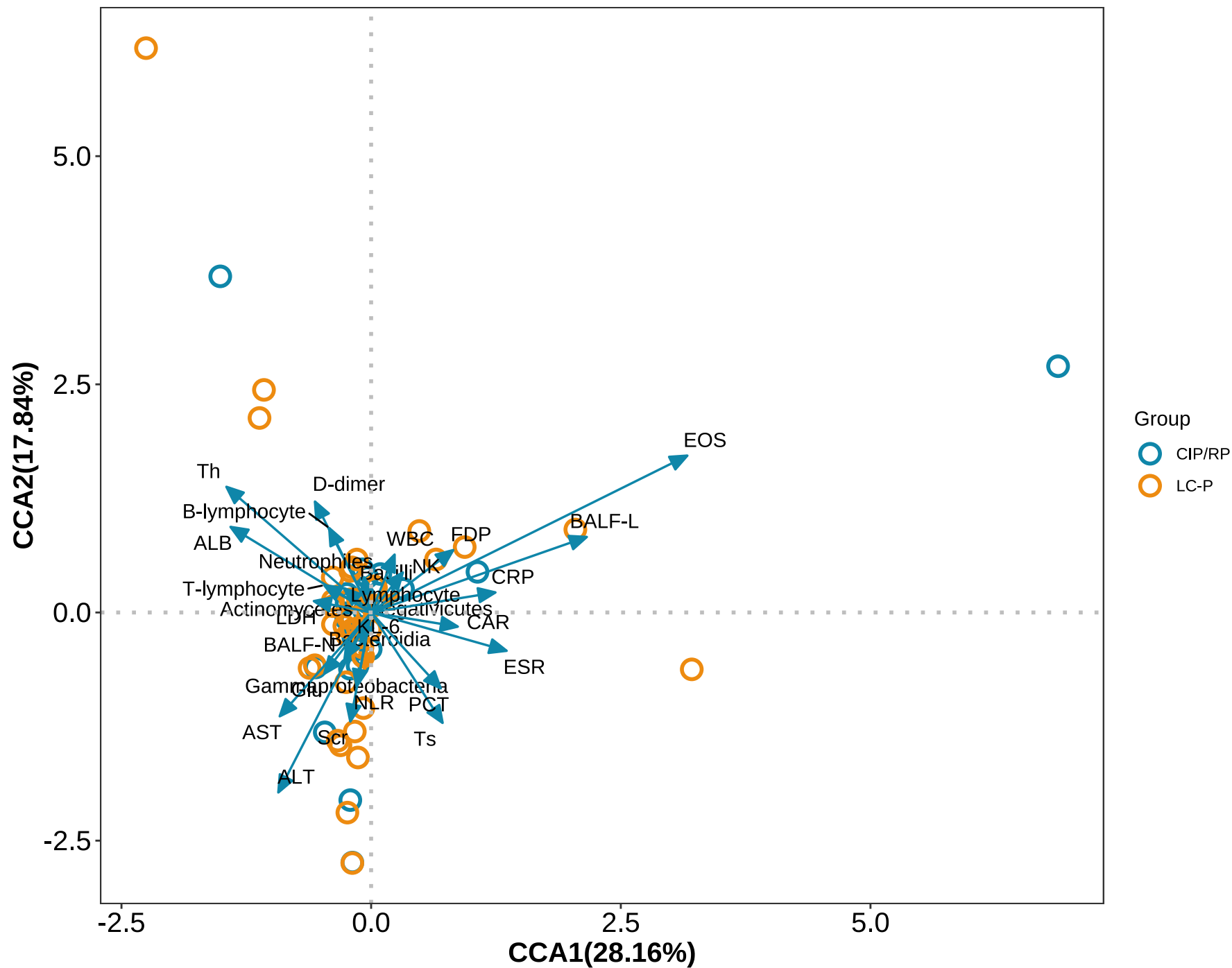

Supplement: Supplementary file 2 [file DataSheet1.zip › Data-all result/rda/Group_class_cca.pdf]

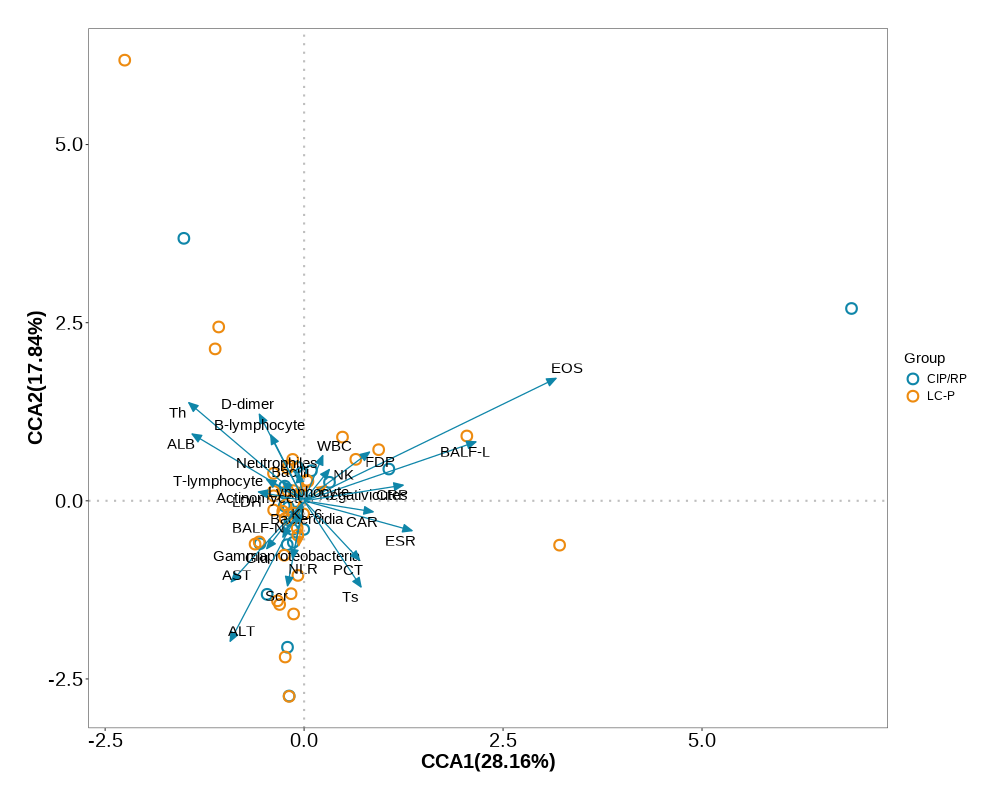

Supplement: Supplementary file 2 [file DataSheet1.zip › Data-all result/rda/Group_class_cca.png]

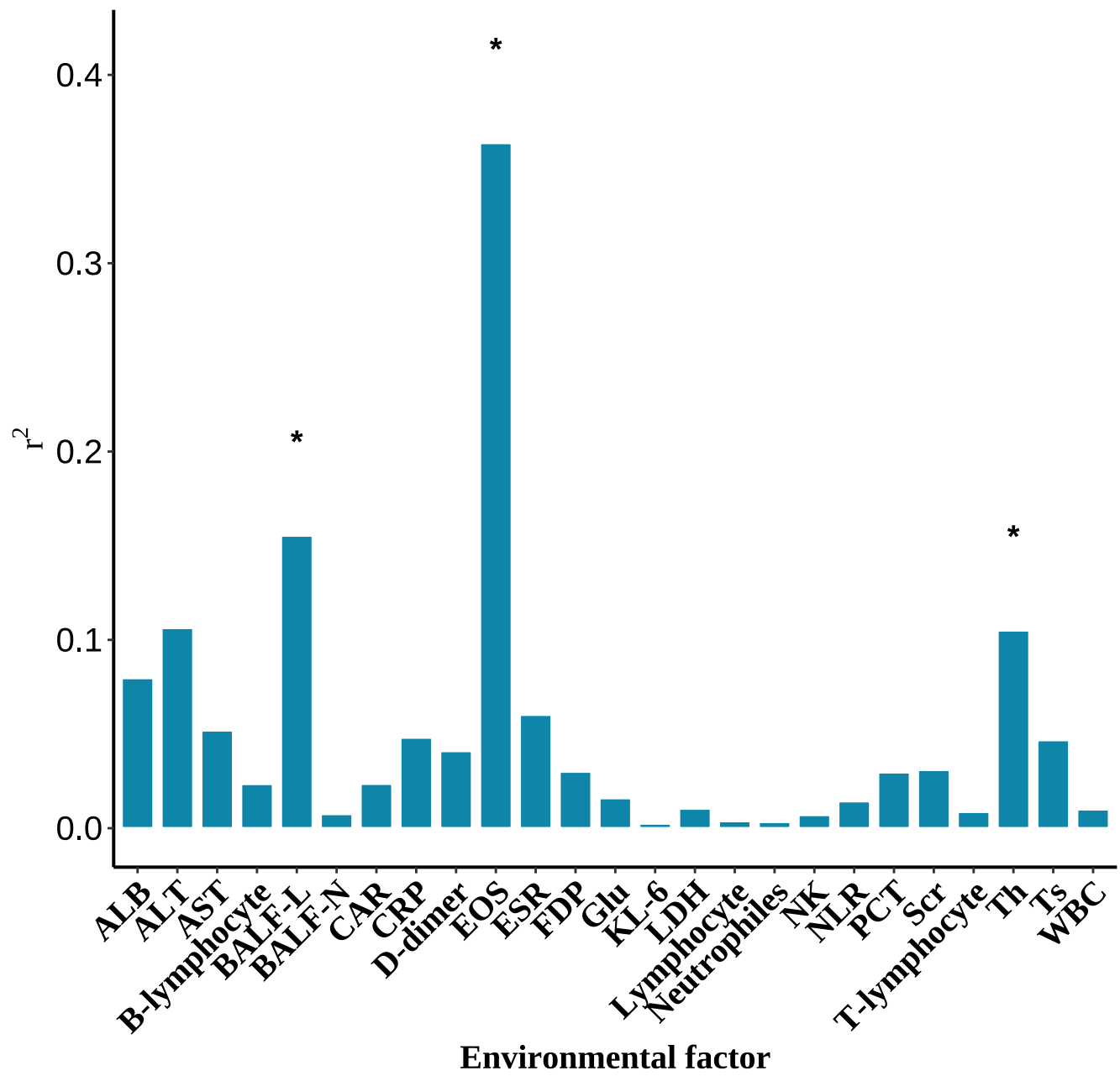

Supplement: Supplementary file 2 [file DataSheet1.zip › Data-all result/rda/Group_class_Env.pdf]

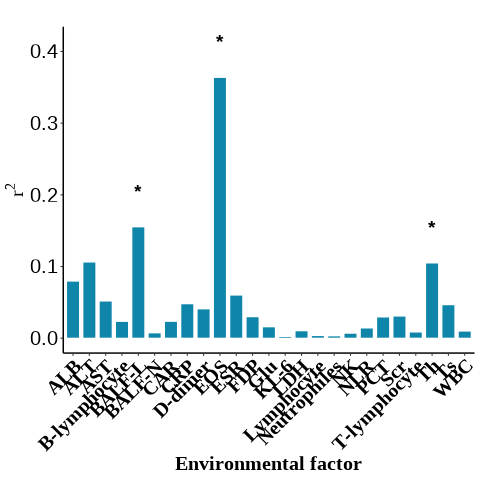

Supplement: Supplementary file 2 [file DataSheet1.zip › Data-all result/rda/Group_class_Env.png]

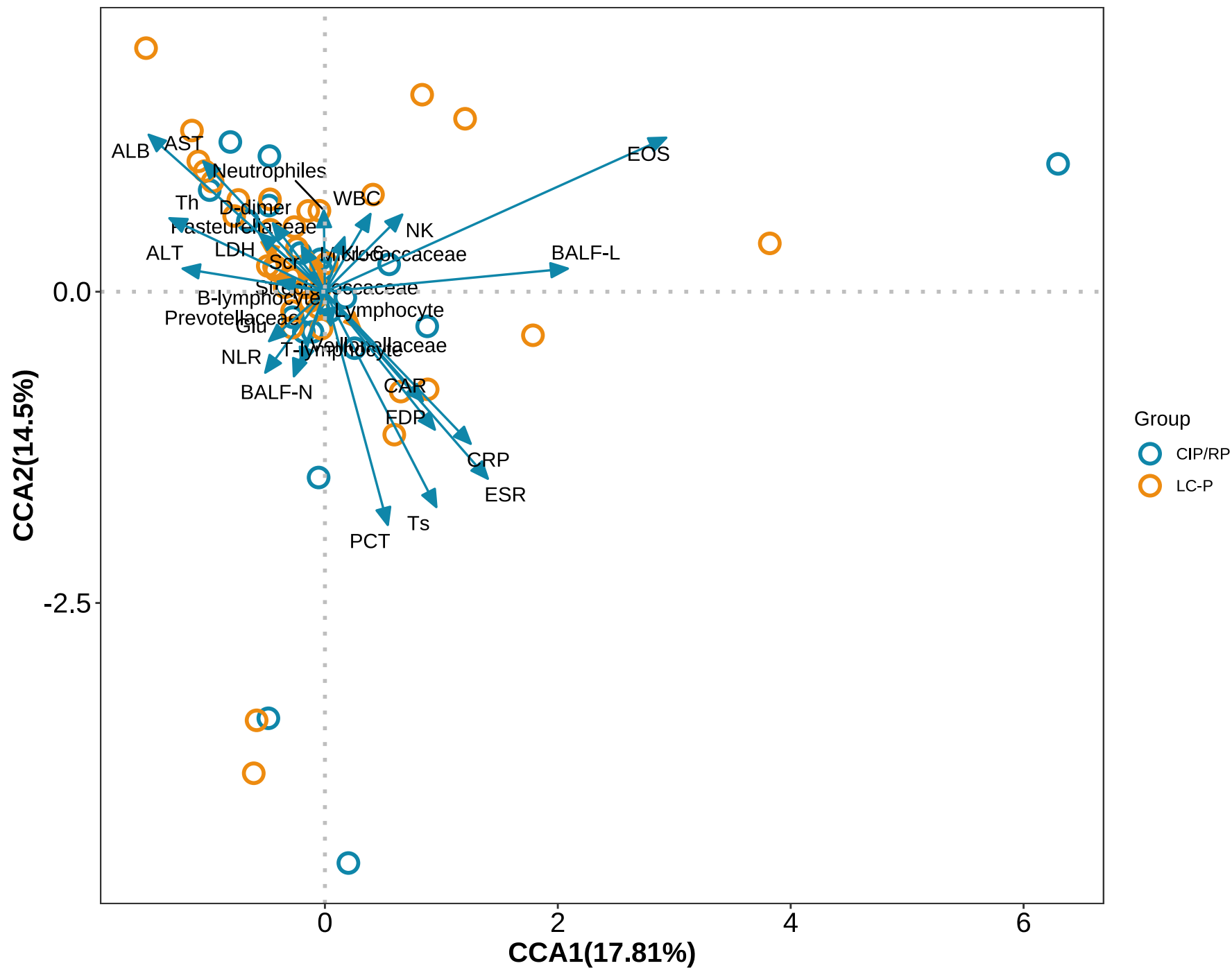

Supplement: Supplementary file 2 [file DataSheet1.zip › Data-all result/rda/Group_family_cca.pdf]

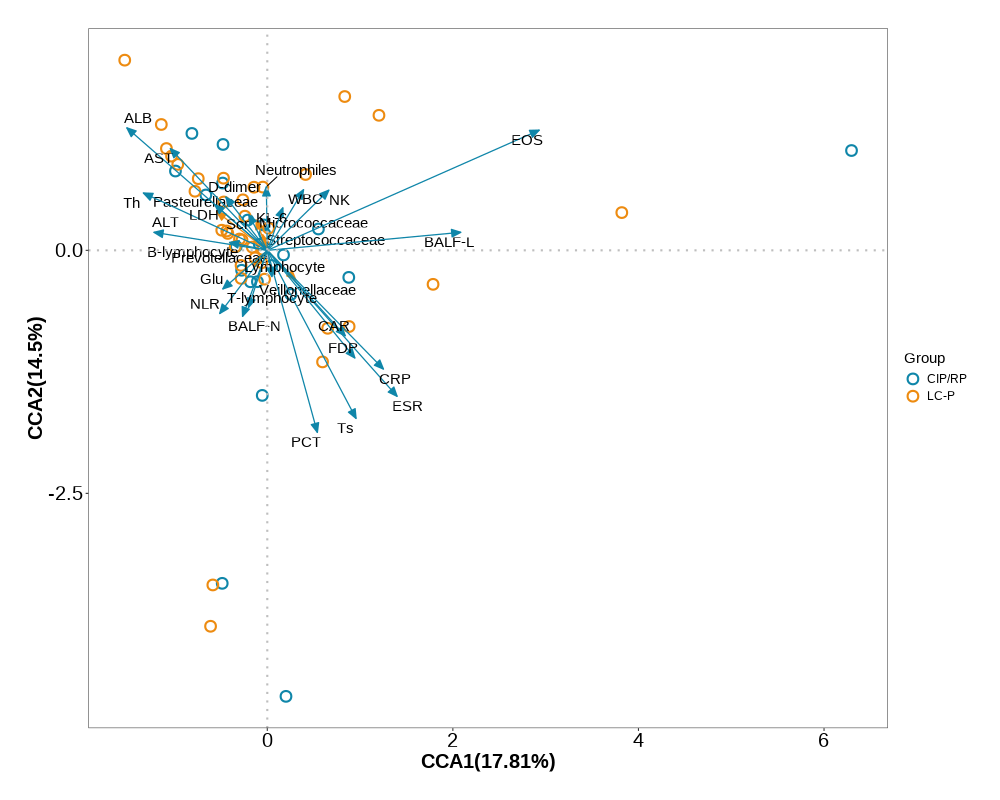

Supplement: Supplementary file 2 [file DataSheet1.zip › Data-all result/rda/Group_family_cca.png]

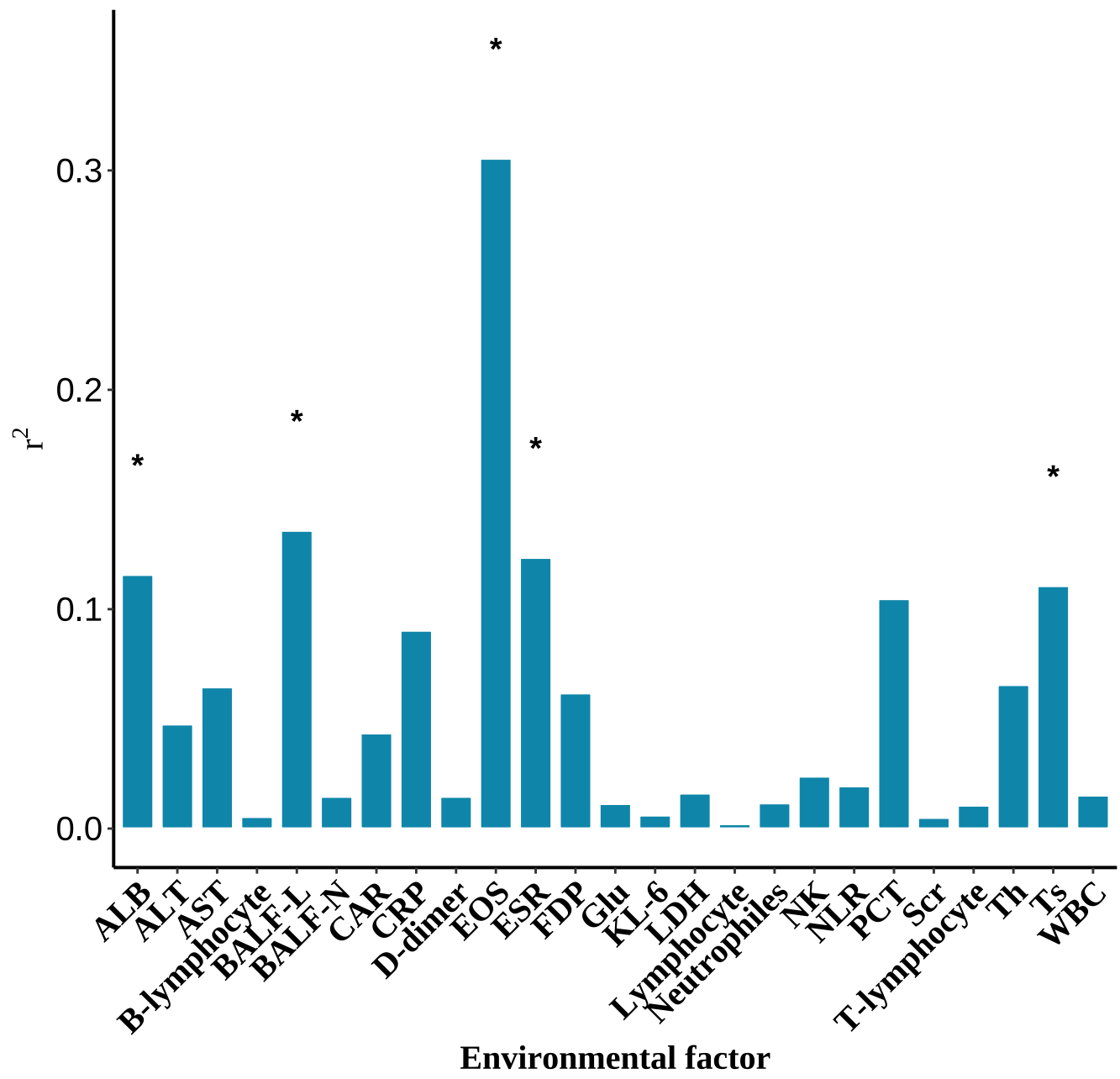

Supplement: Supplementary file 2 [file DataSheet1.zip › Data-all result/rda/Group_family_Env.pdf]

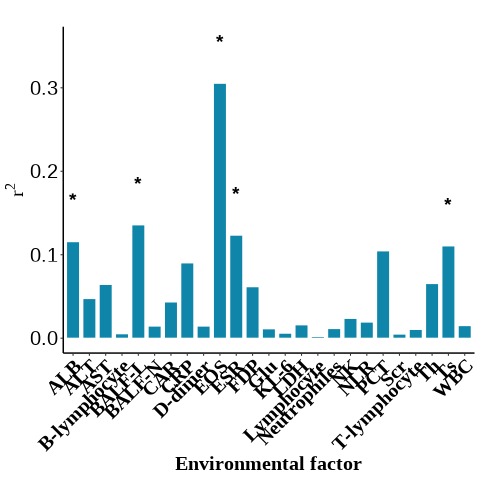

Supplement: Supplementary file 2 [file DataSheet1.zip › Data-all result/rda/Group_family_Env.png]

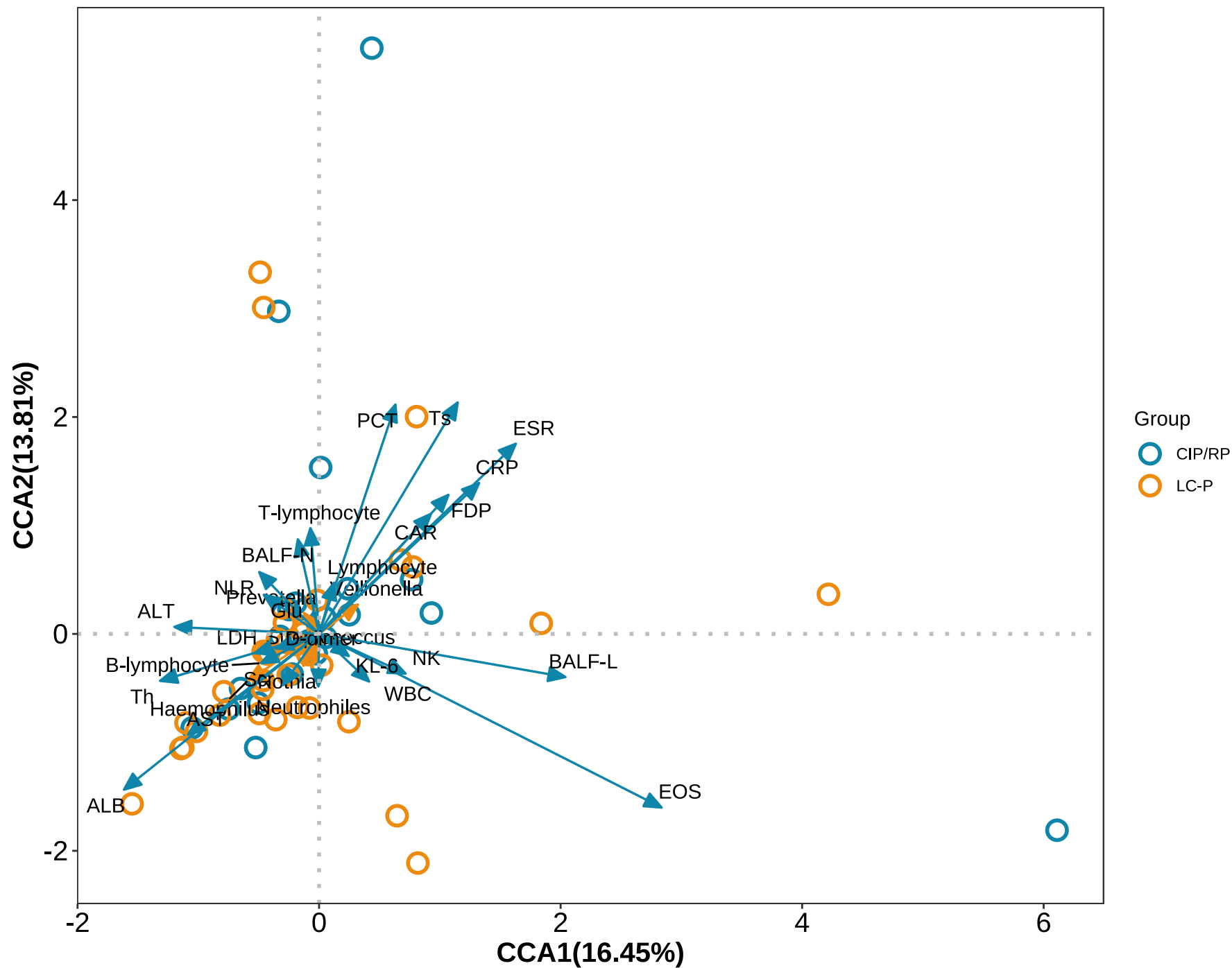

Supplement: Supplementary file 2 [file DataSheet1.zip › Data-all result/rda/Group_genus_cca.pdf]

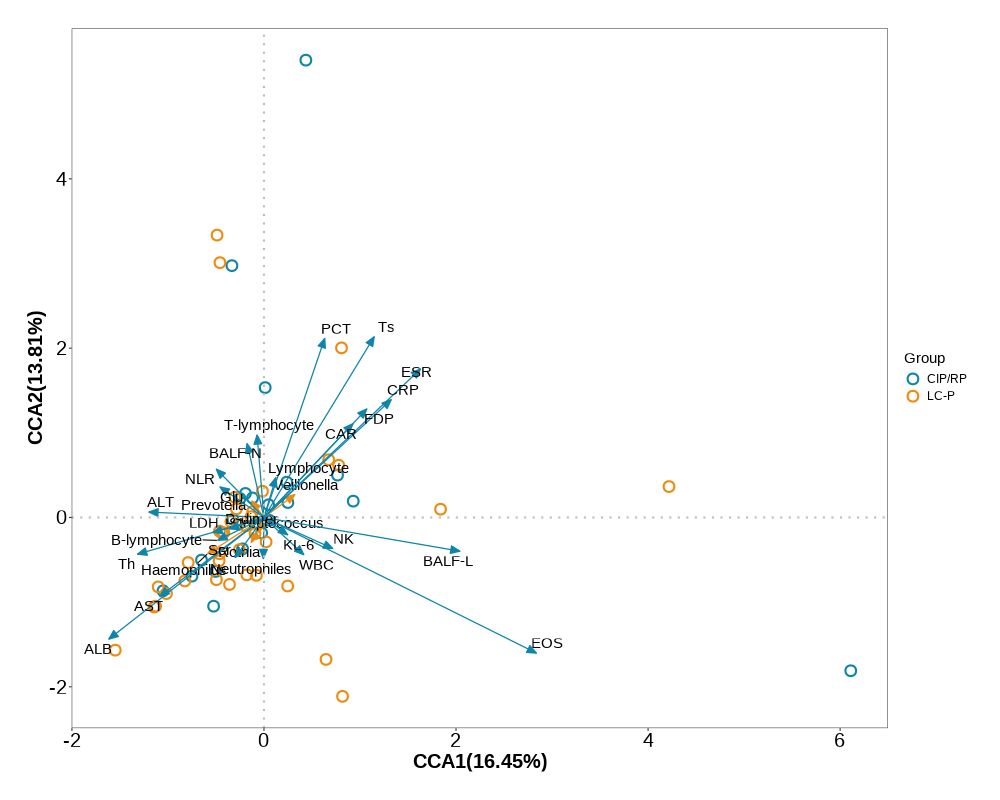

Supplement: Supplementary file 2 [file DataSheet1.zip › Data-all result/rda/Group_genus_cca.png]

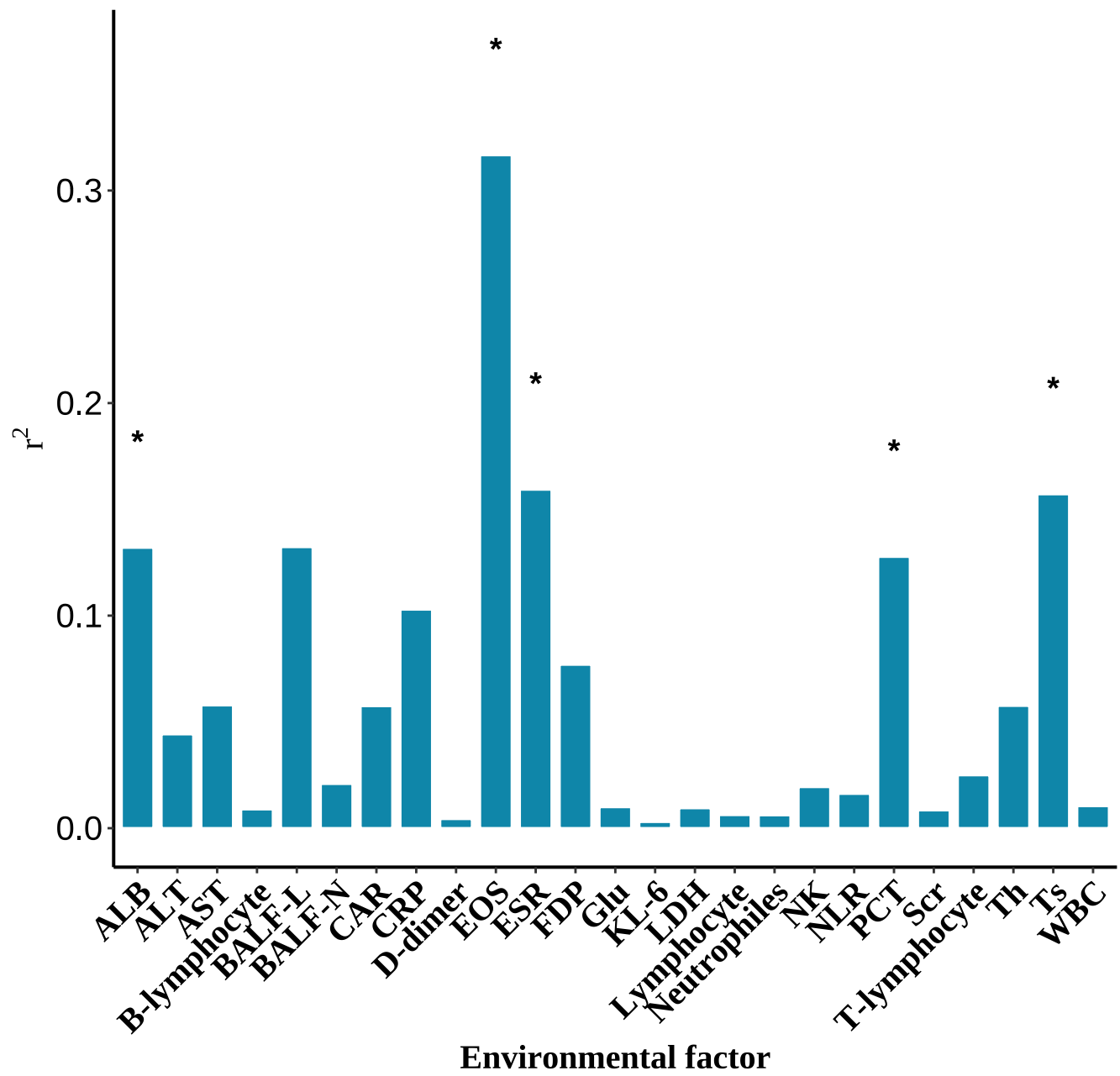

Supplement: Supplementary file 2 [file DataSheet1.zip › Data-all result/rda/Group_genus_Env.pdf]

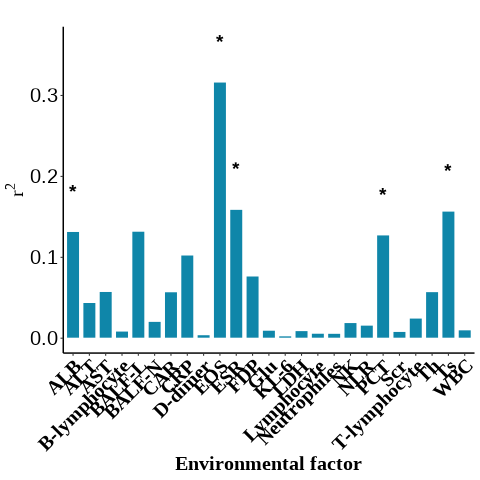

Supplement: Supplementary file 2 [file DataSheet1.zip › Data-all result/rda/Group_genus_Env.png]

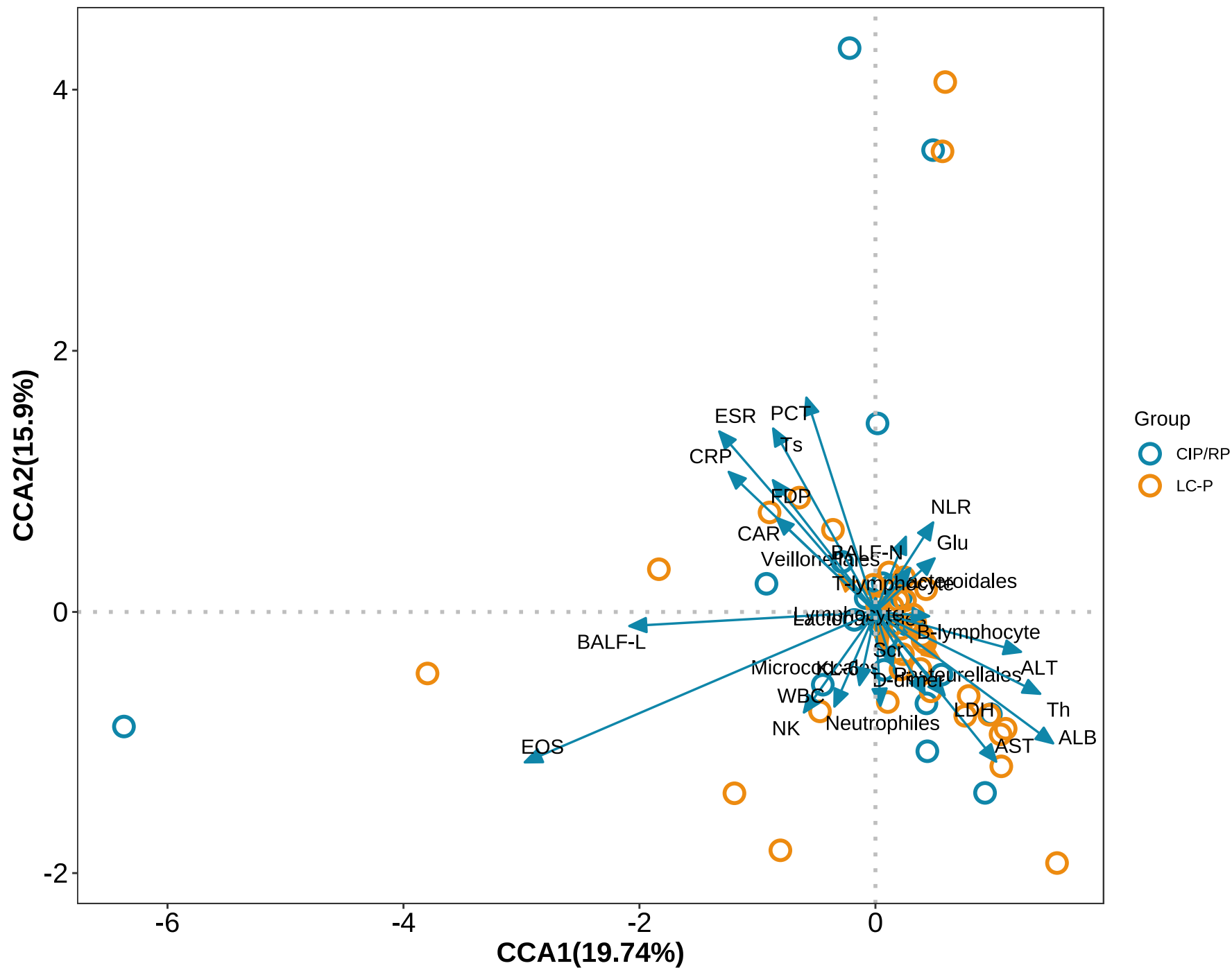

Supplement: Supplementary file 2 [file DataSheet1.zip › Data-all result/rda/Group_order_cca.pdf]

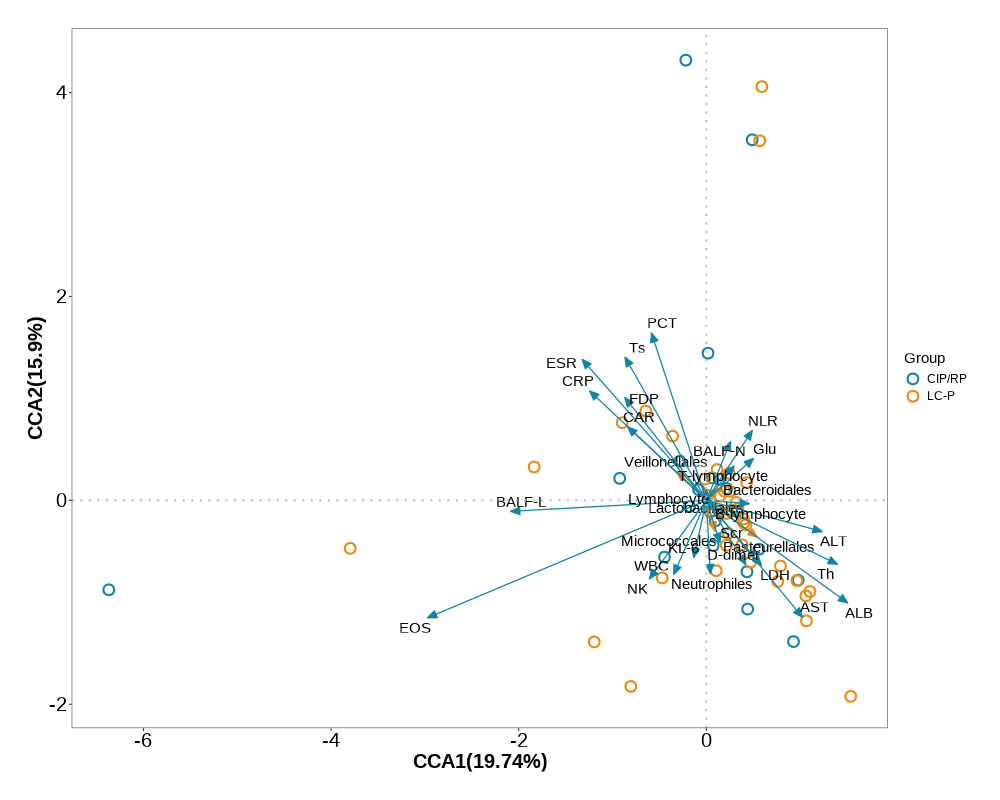

Supplement: Supplementary file 2 [file DataSheet1.zip › Data-all result/rda/Group_order_cca.png]

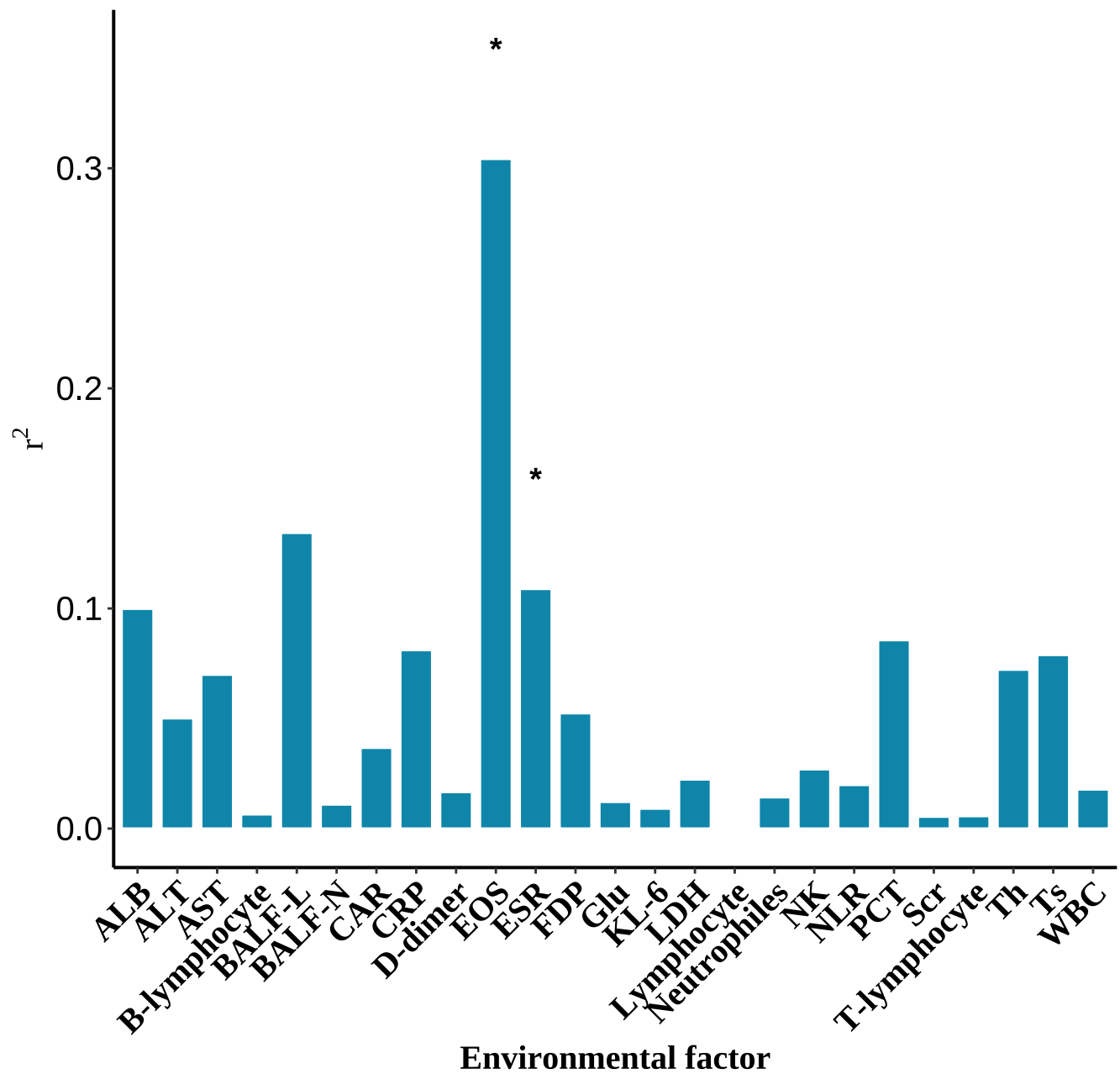

Supplement: Supplementary file 2 [file DataSheet1.zip › Data-all result/rda/Group_order_Env.pdf]

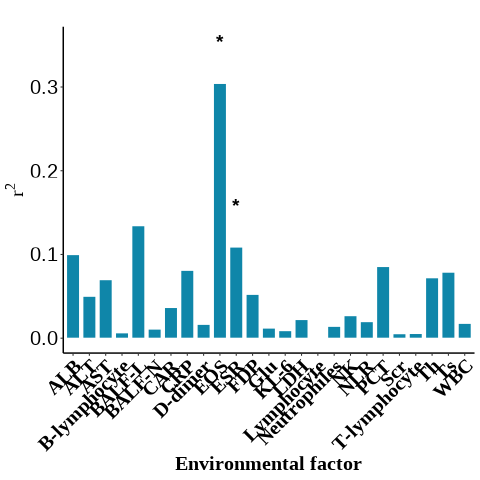

Supplement: Supplementary file 2 [file DataSheet1.zip › Data-all result/rda/Group_order_Env.png]

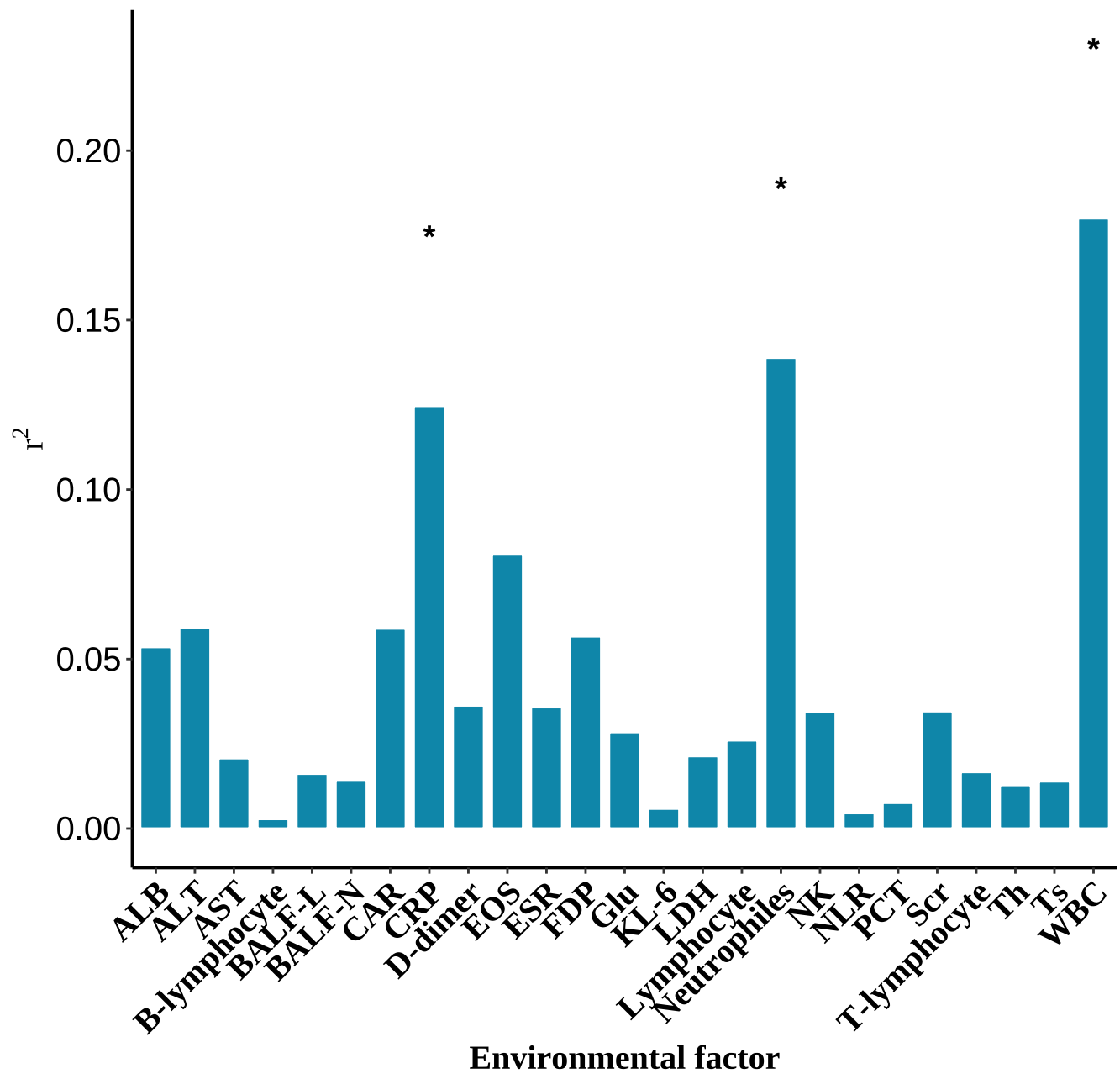

Supplement: Supplementary file 2 [file DataSheet1.zip › Data-all result/rda/Group_phylum_Env.pdf]

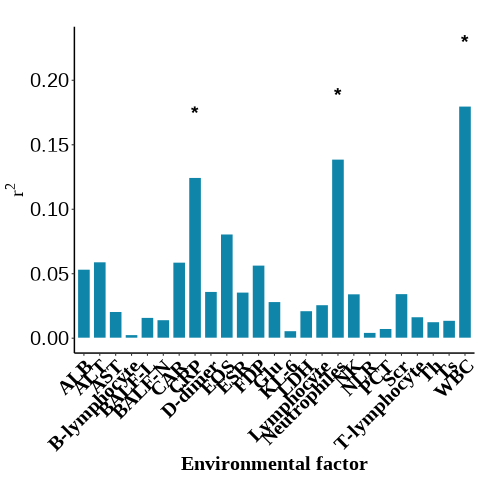

Supplement: Supplementary file 2 [file DataSheet1.zip › Data-all result/rda/Group_phylum_Env.png]

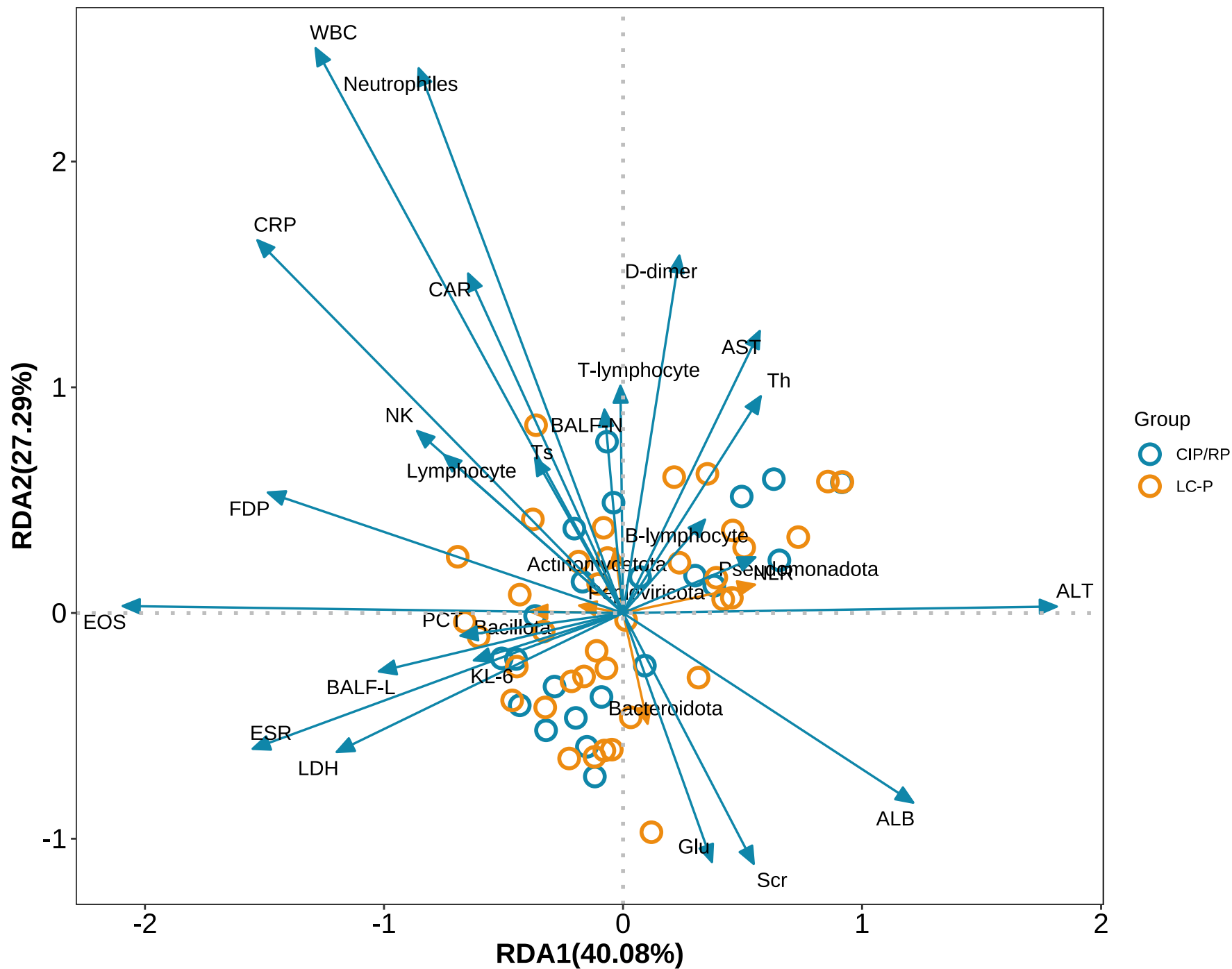

Supplement: Supplementary file 2 [file DataSheet1.zip › Data-all result/rda/Group_phylum_rda.pdf]

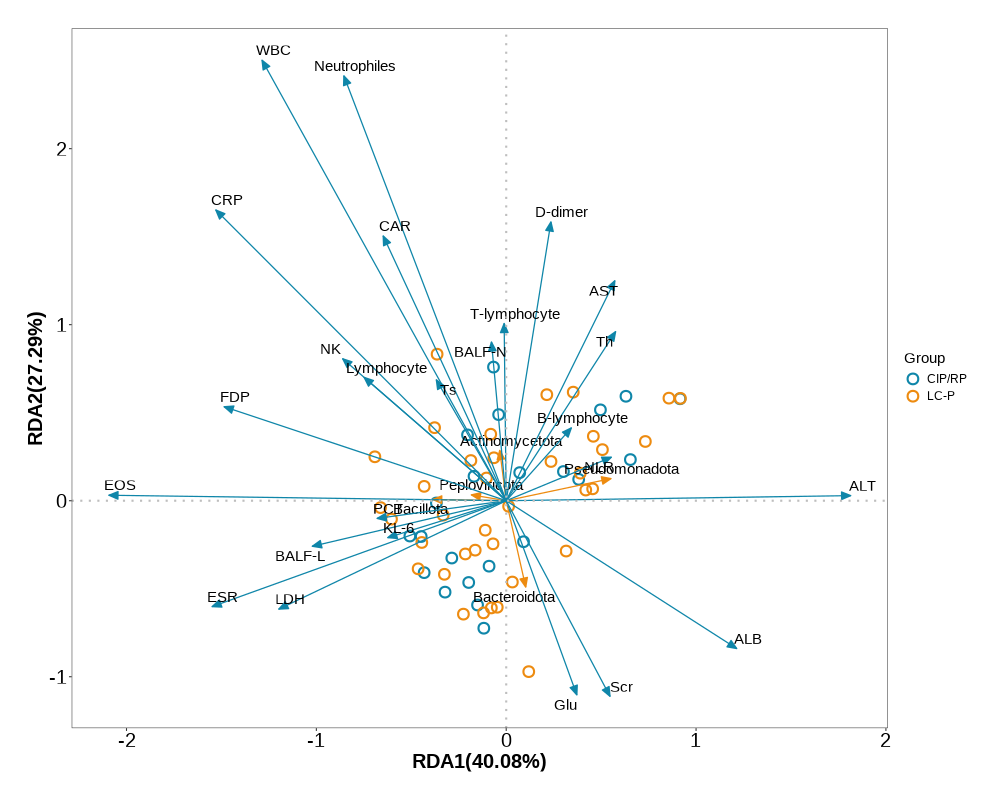

Supplement: Supplementary file 2 [file DataSheet1.zip › Data-all result/rda/Group_phylum_rda.png]

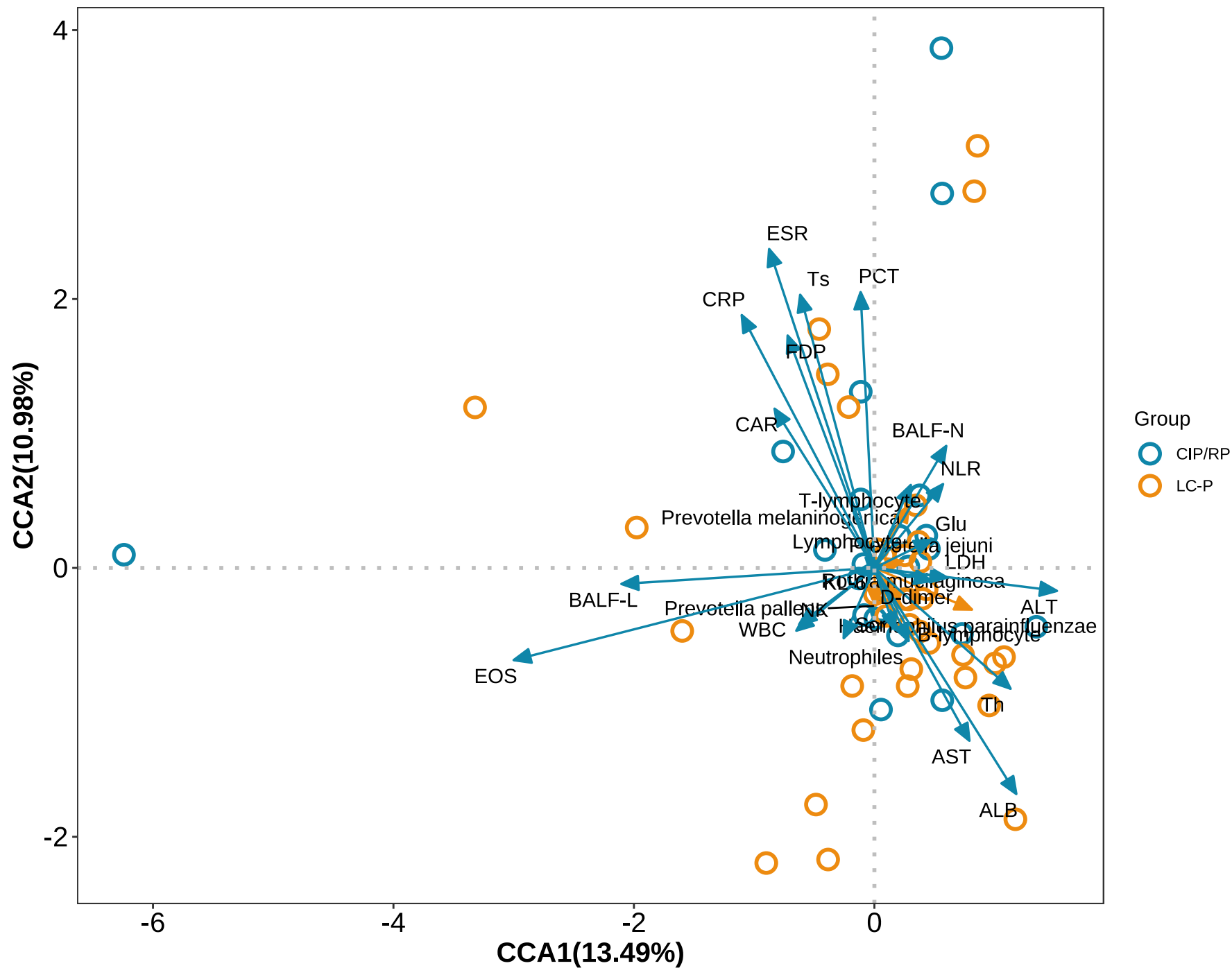

Supplement: Supplementary file 2 [file DataSheet1.zip › Data-all result/rda/Group_species_cca.pdf]

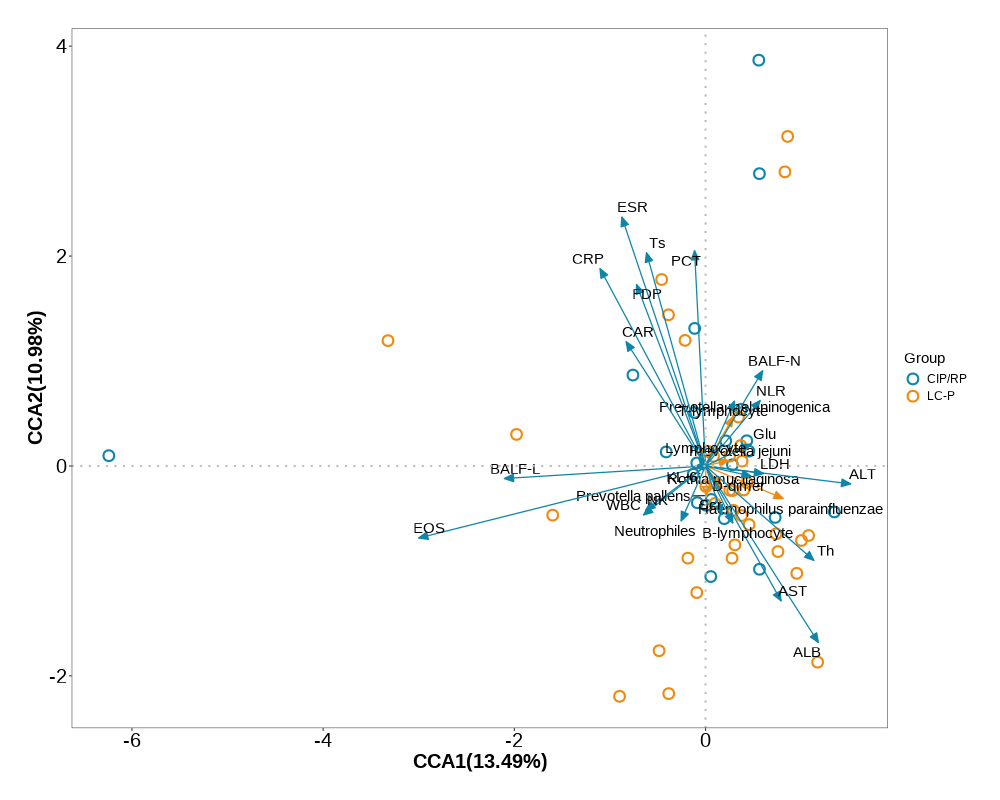

Supplement: Supplementary file 2 [file DataSheet1.zip › Data-all result/rda/Group_species_cca.png]

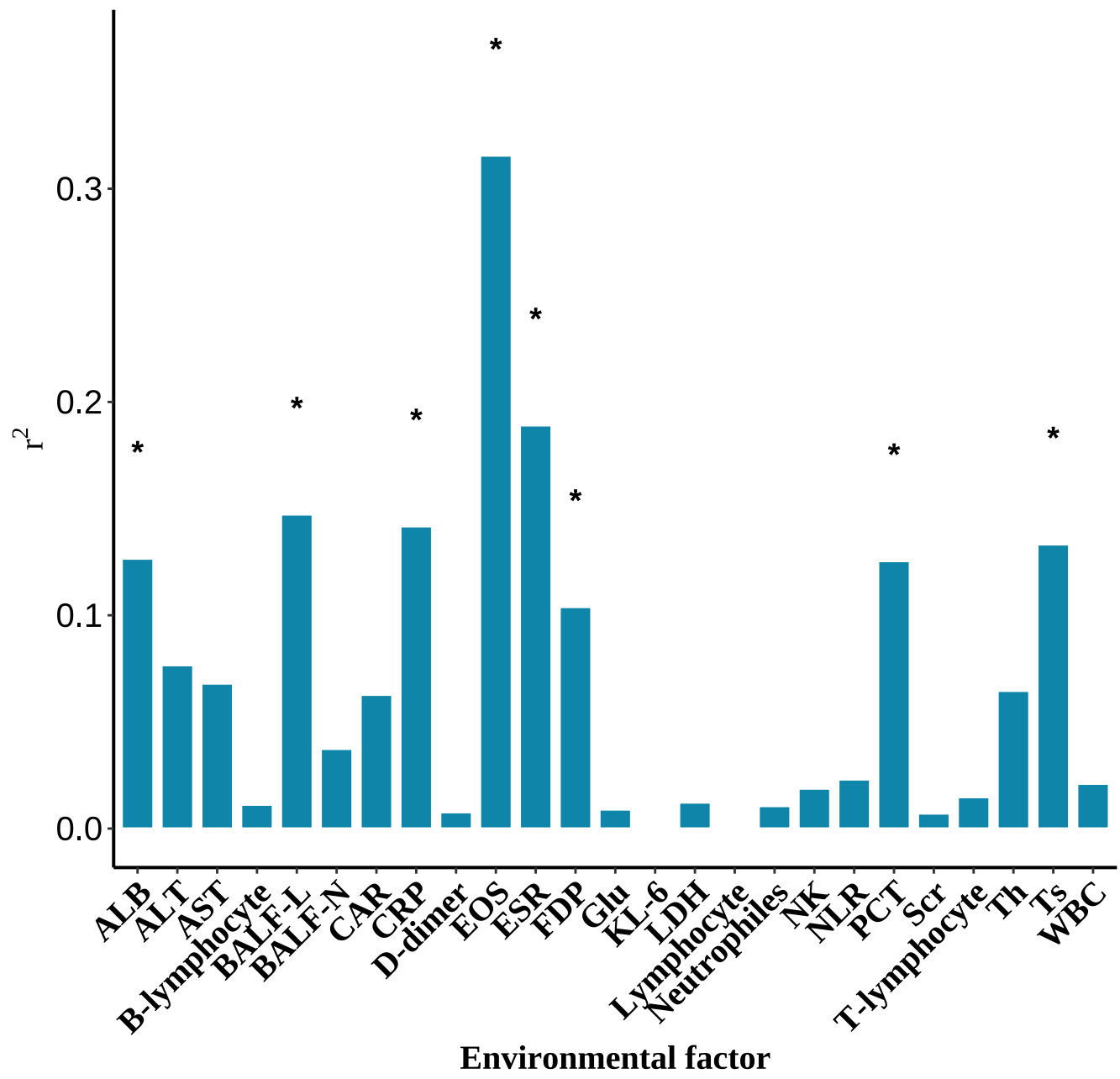

Supplement: Supplementary file 2 [file DataSheet1.zip › Data-all result/rda/Group_species_Env.pdf]

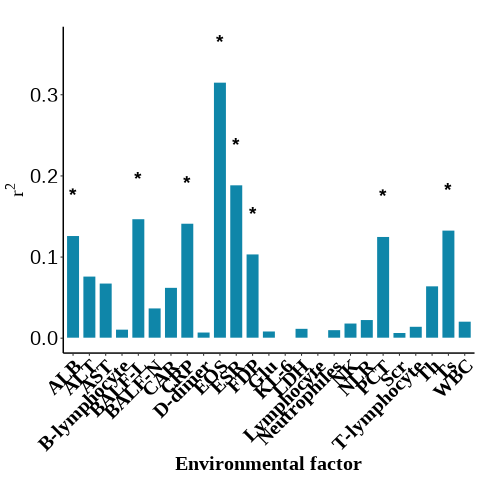

Supplement: Supplementary file 2 [file DataSheet1.zip › Data-all result/rda/Group_species_Env.png]

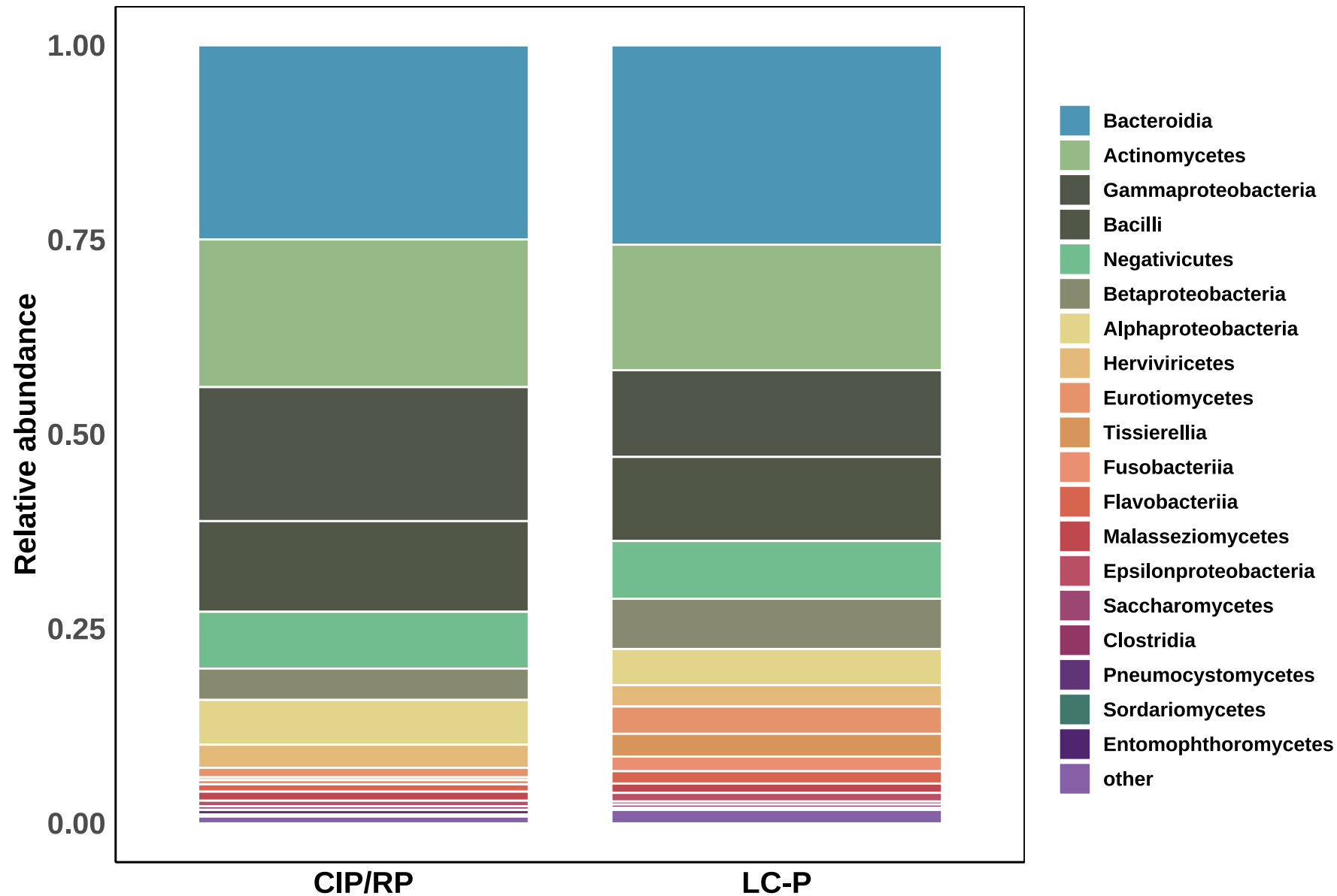

Supplement: Supplementary file 2 [file DataSheet1.zip › Data-all result/TaxonomicProfiling/Group_class_taxonomy_stacked_bar.pdf]

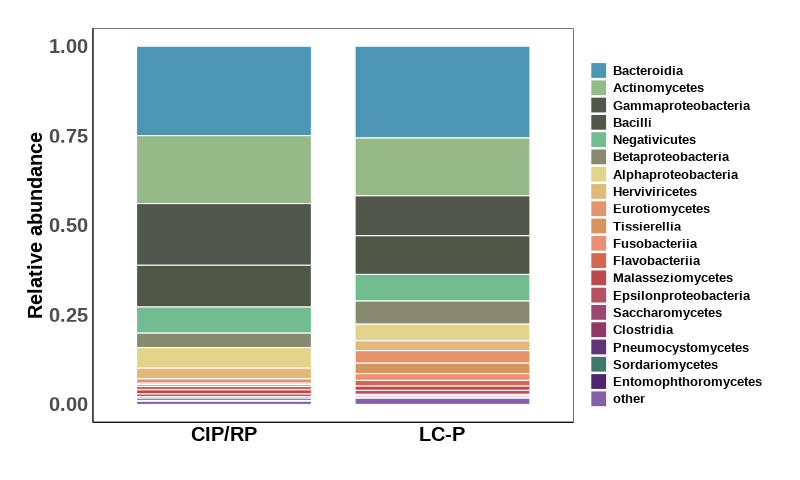

Supplement: Supplementary file 2 [file DataSheet1.zip › Data-all result/TaxonomicProfiling/Group_class_taxonomy_stacked_bar.png]

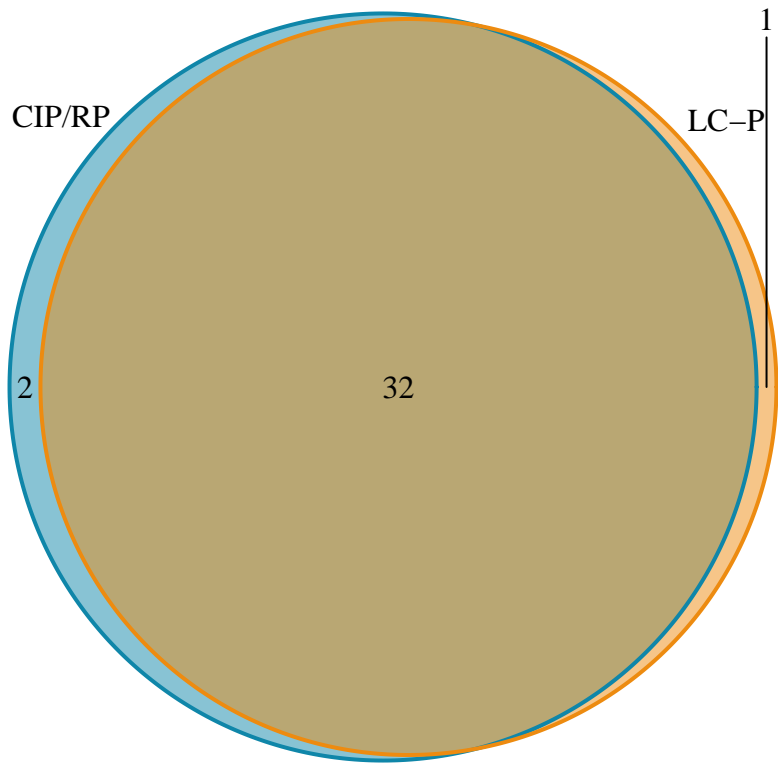

Supplement: Supplementary file 2 [file DataSheet1.zip › Data-all result/TaxonomicProfiling/Group_class_venn.pdf]

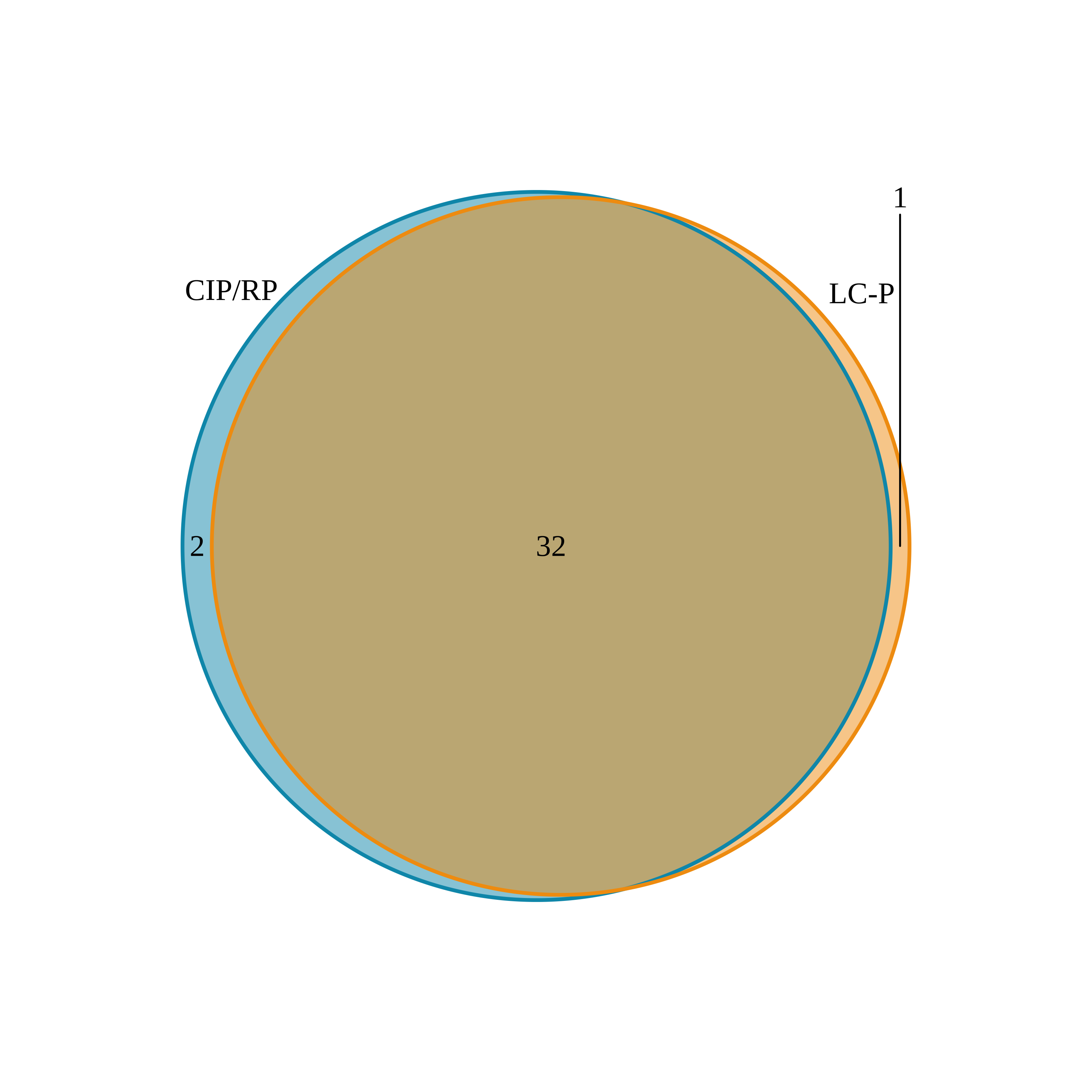

Supplement: Supplementary file 2 [file DataSheet1.zip › Data-all result/TaxonomicProfiling/Group_class_venn.png]

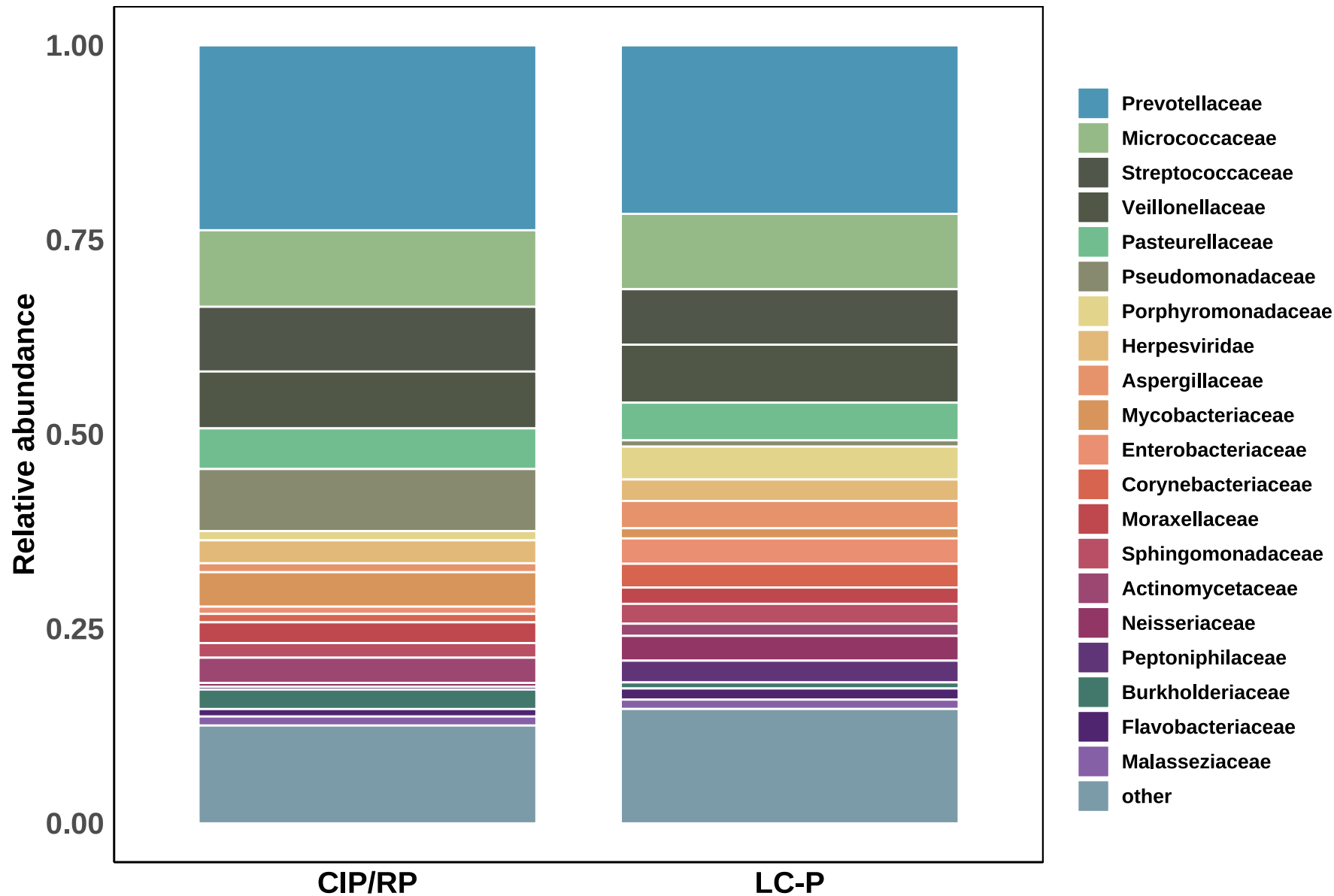

Supplement: Supplementary file 2 [file DataSheet1.zip › Data-all result/TaxonomicProfiling/Group_family_taxonomy_stacked_bar.pdf]

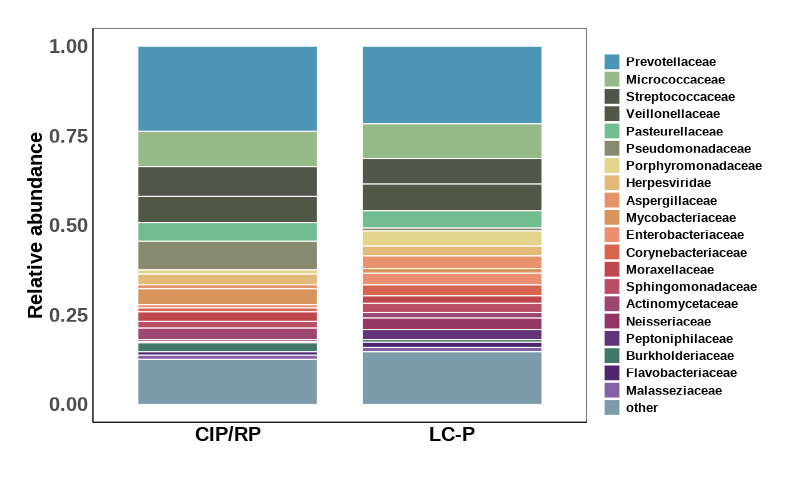

Supplement: Supplementary file 2 [file DataSheet1.zip › Data-all result/TaxonomicProfiling/Group_family_taxonomy_stacked_bar.png]

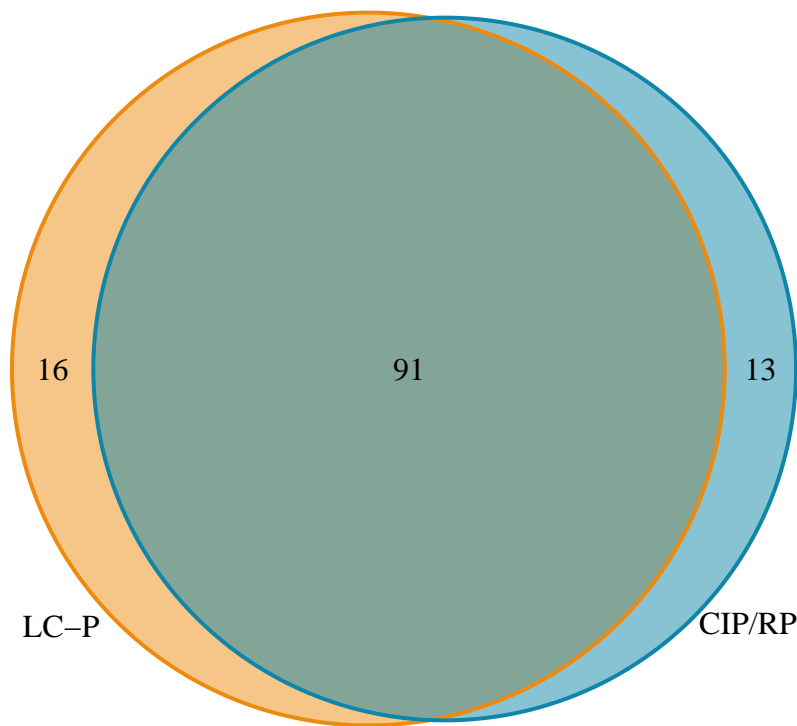

Supplement: Supplementary file 2 [file DataSheet1.zip › Data-all result/TaxonomicProfiling/Group_family_venn.pdf]

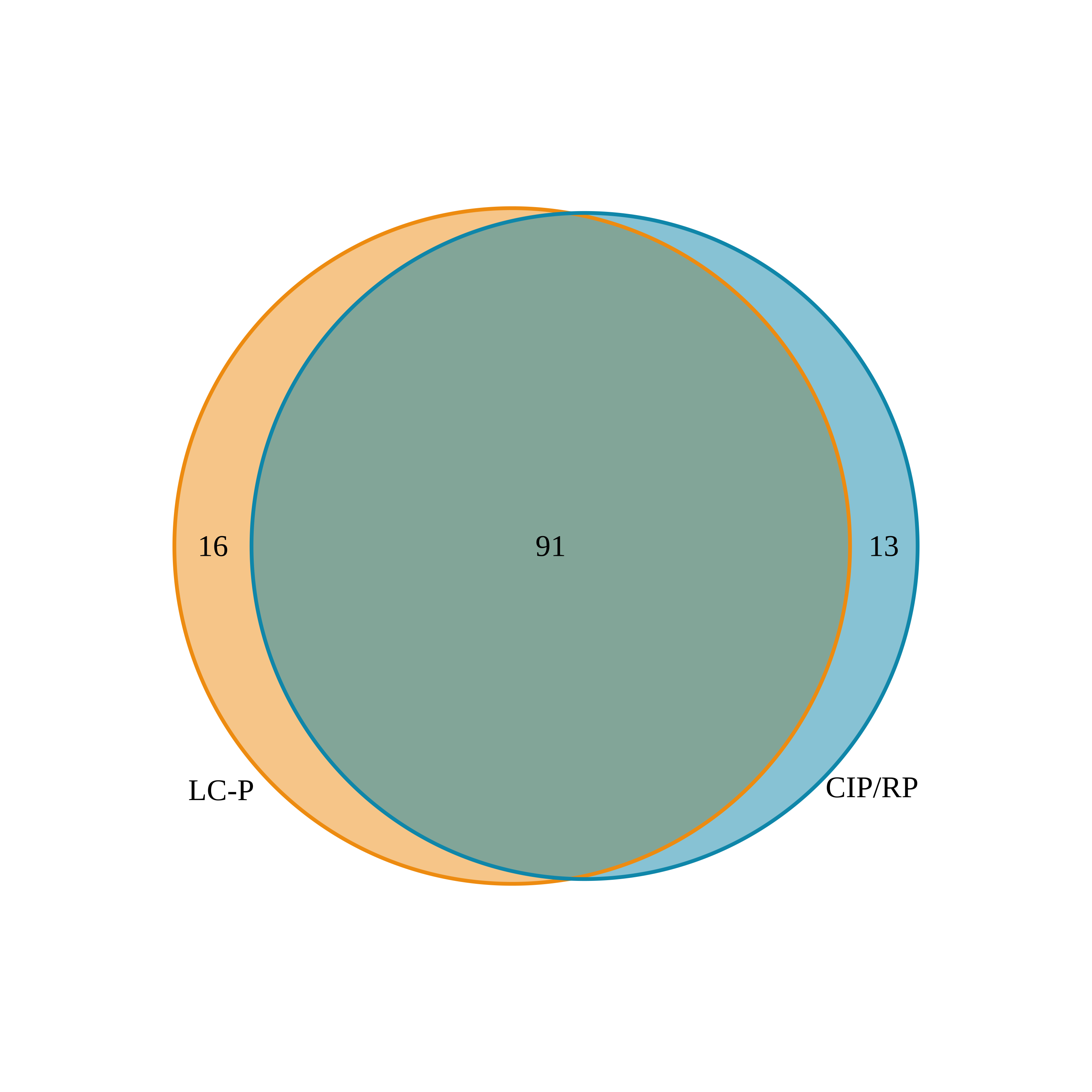

Supplement: Supplementary file 2 [file DataSheet1.zip › Data-all result/TaxonomicProfiling/Group_family_venn.png]

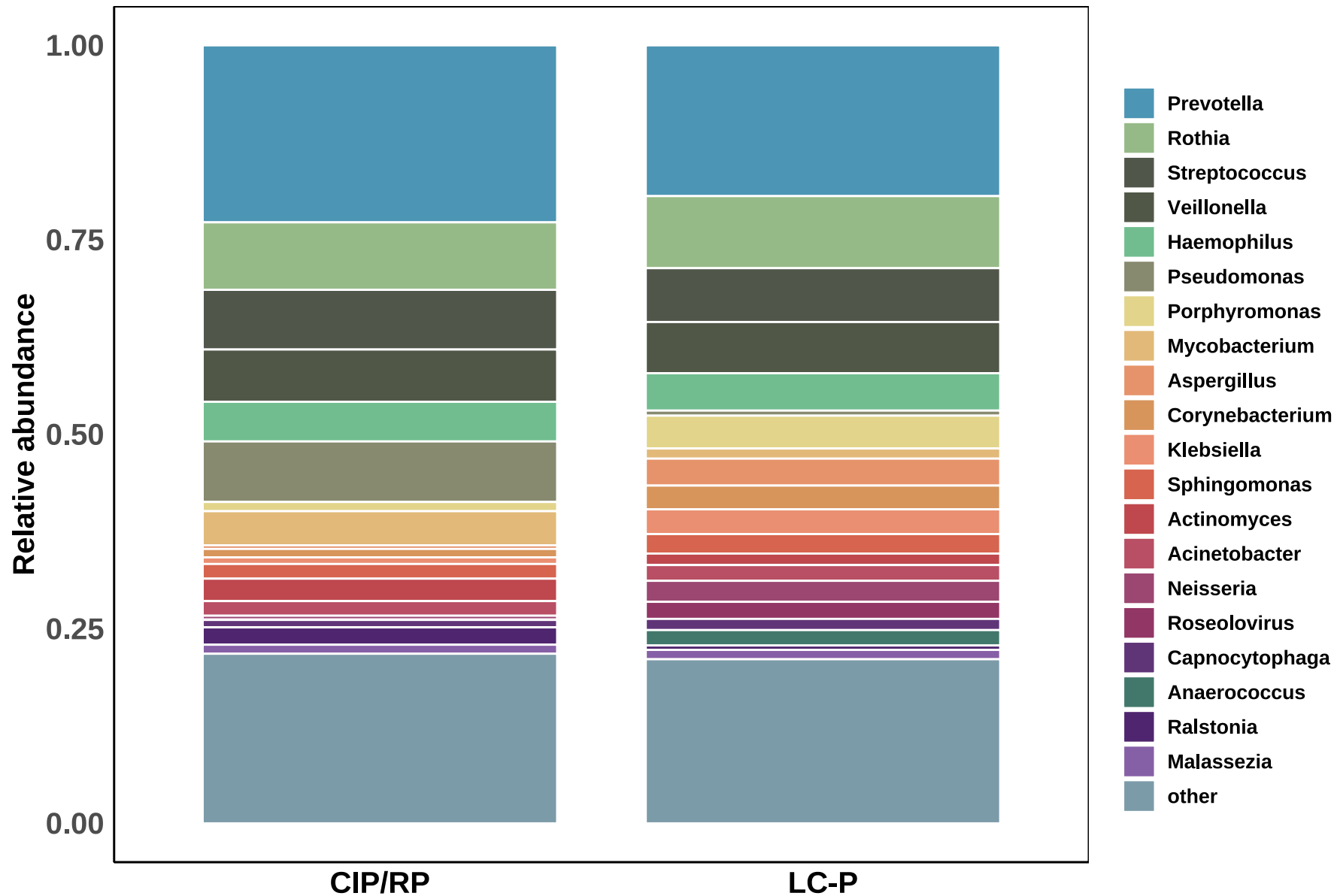

Supplement: Supplementary file 2 [file DataSheet1.zip › Data-all result/TaxonomicProfiling/Group_genus_taxonomy_stacked_bar.pdf]

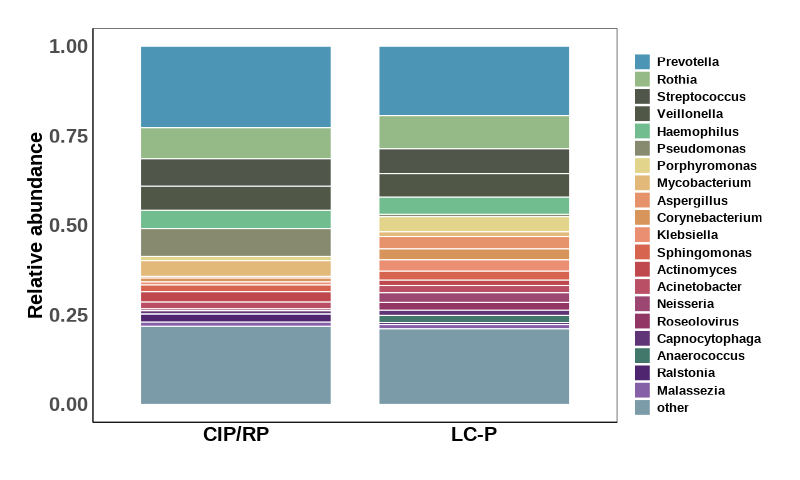

Supplement: Supplementary file 2 [file DataSheet1.zip › Data-all result/TaxonomicProfiling/Group_genus_taxonomy_stacked_bar.png]

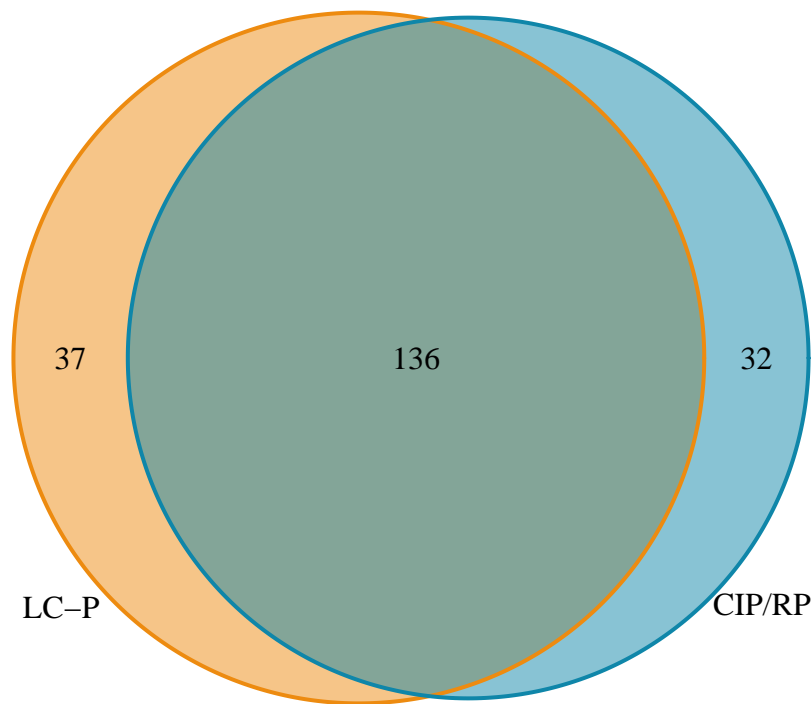

Supplement: Supplementary file 2 [file DataSheet1.zip › Data-all result/TaxonomicProfiling/Group_genus_venn.pdf]

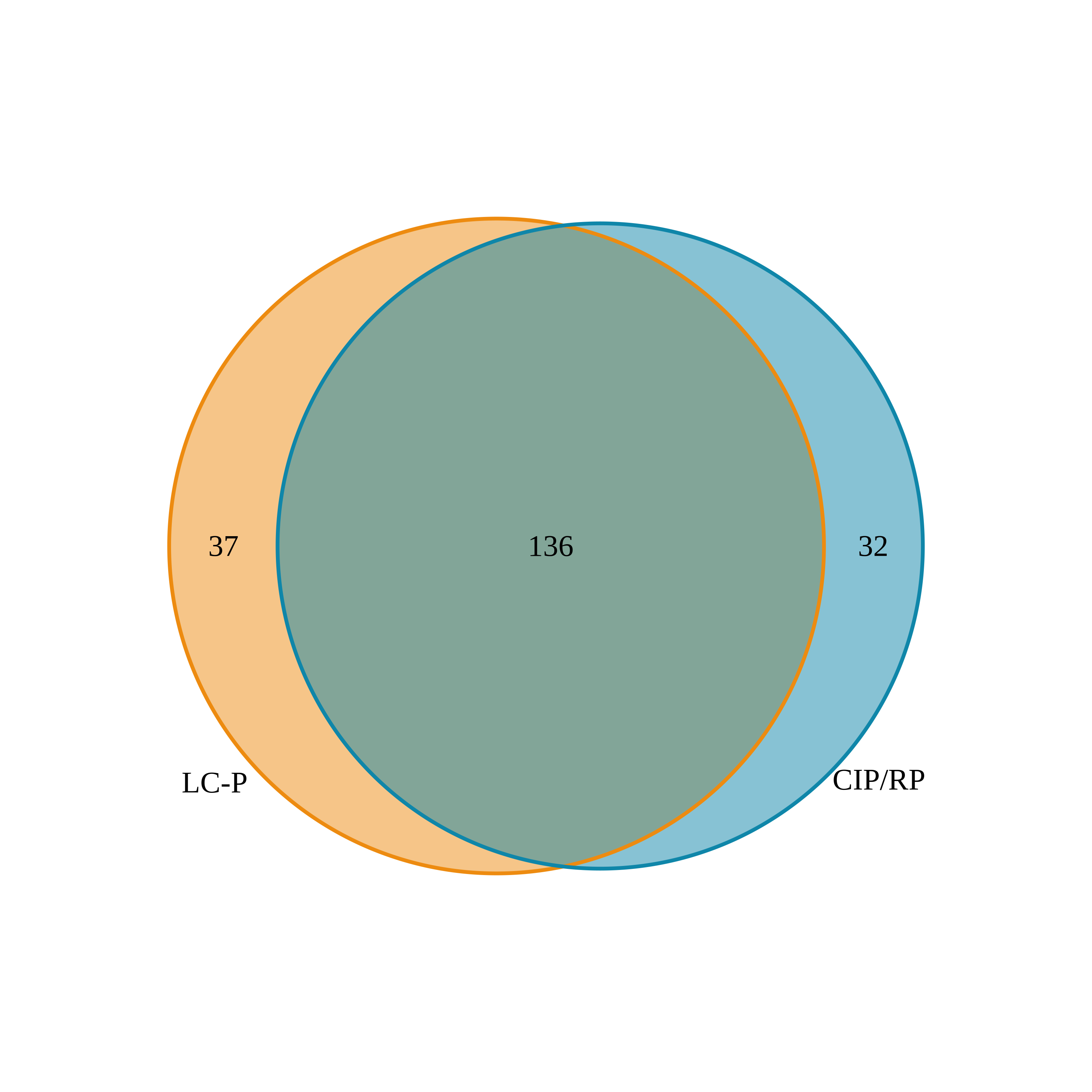

Supplement: Supplementary file 2 [file DataSheet1.zip › Data-all result/TaxonomicProfiling/Group_genus_venn.png]

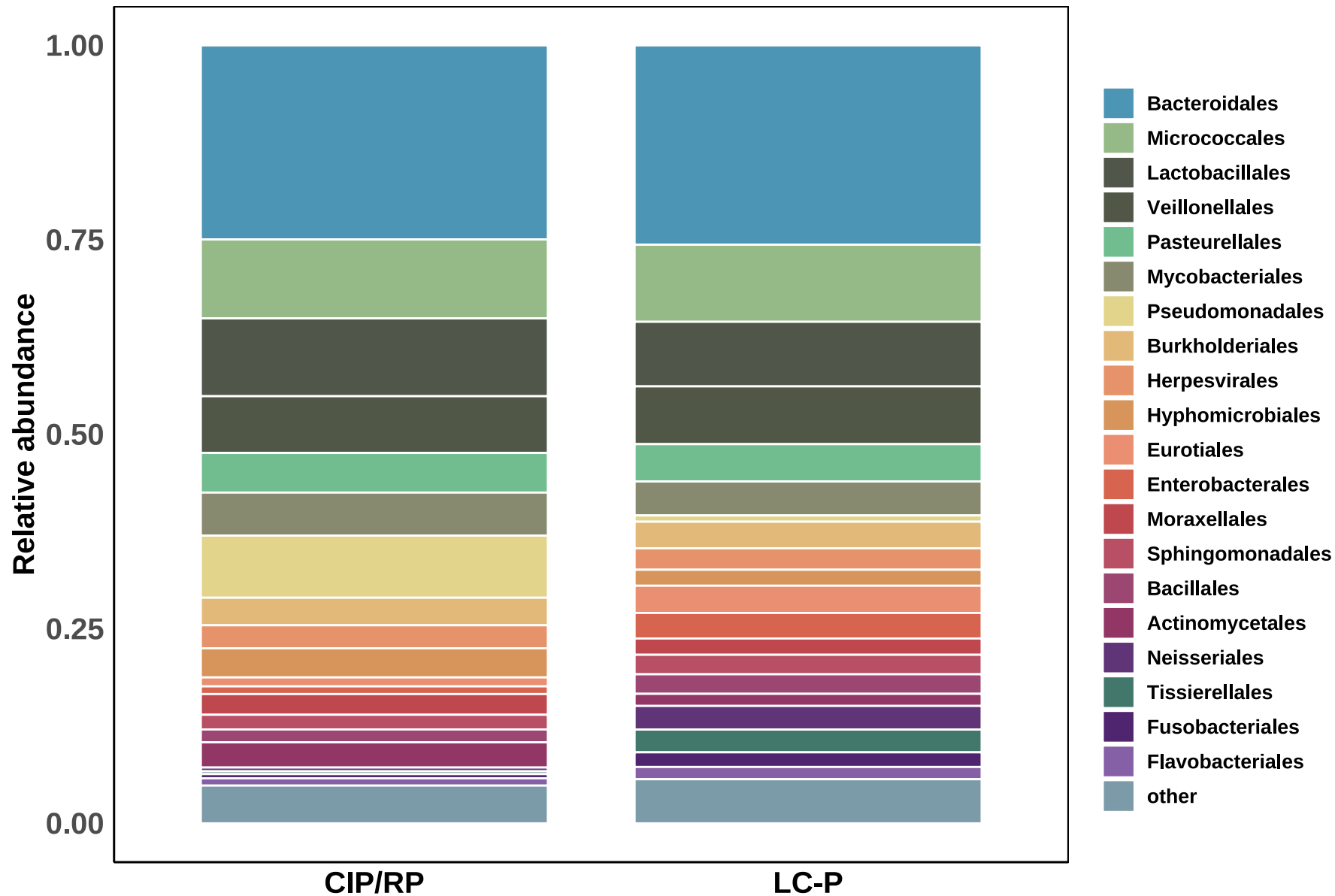

Supplement: Supplementary file 2 [file DataSheet1.zip › Data-all result/TaxonomicProfiling/Group_order_taxonomy_stacked_bar.pdf]

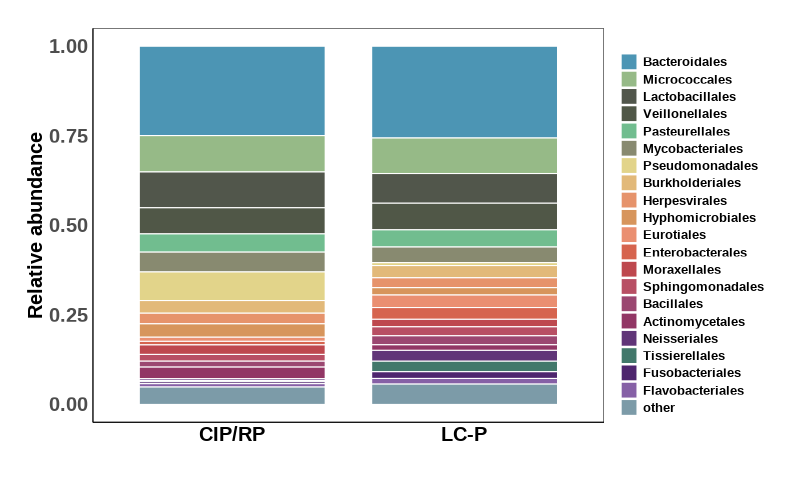

Supplement: Supplementary file 2 [file DataSheet1.zip › Data-all result/TaxonomicProfiling/Group_order_taxonomy_stacked_bar.png]

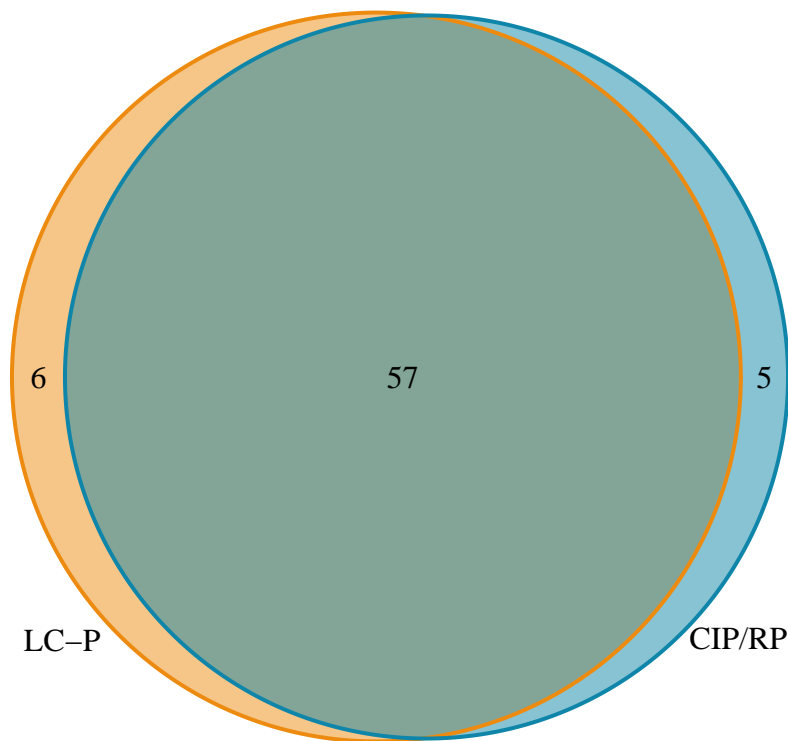

Supplement: Supplementary file 2 [file DataSheet1.zip › Data-all result/TaxonomicProfiling/Group_order_venn.pdf]

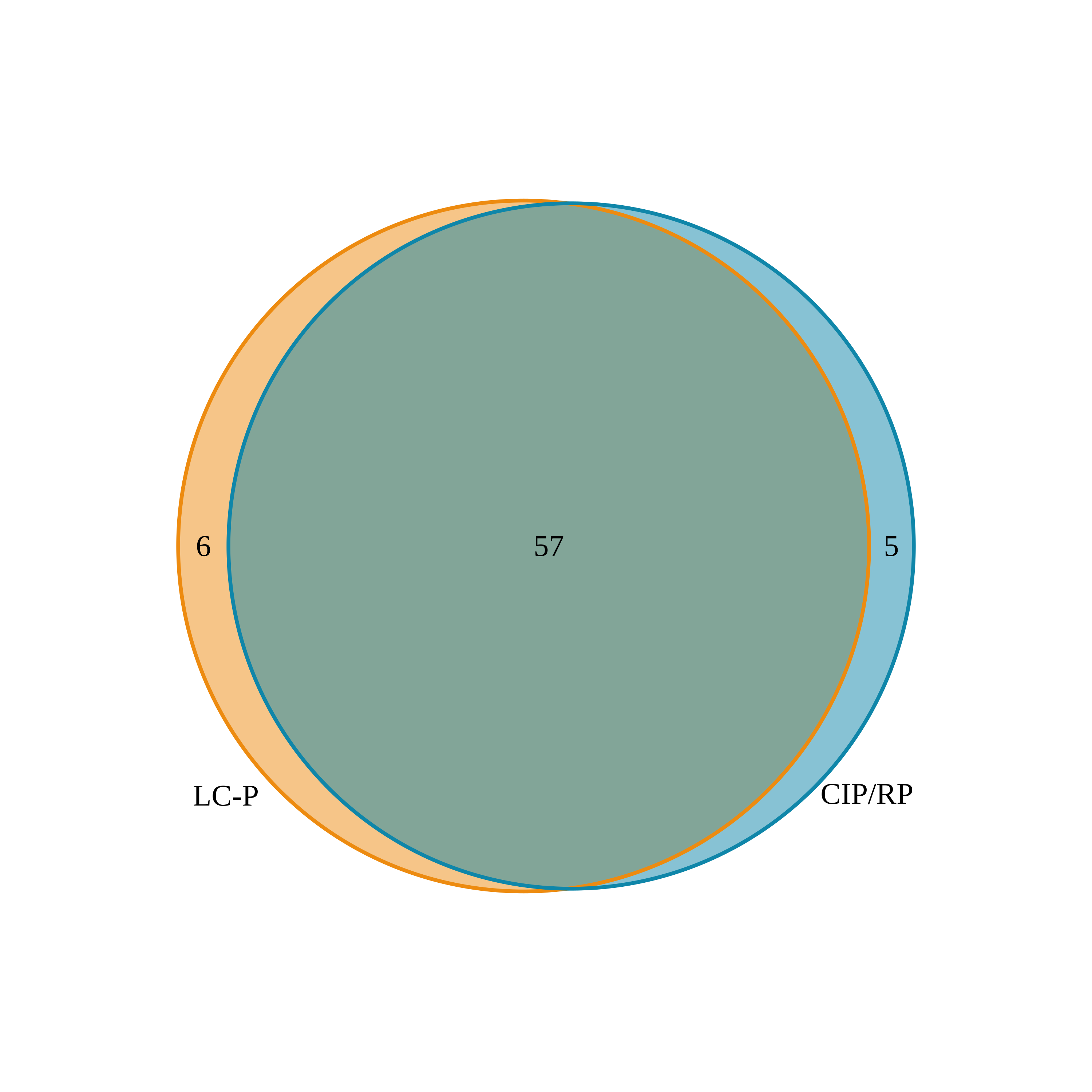

Supplement: Supplementary file 2 [file DataSheet1.zip › Data-all result/TaxonomicProfiling/Group_order_venn.png]

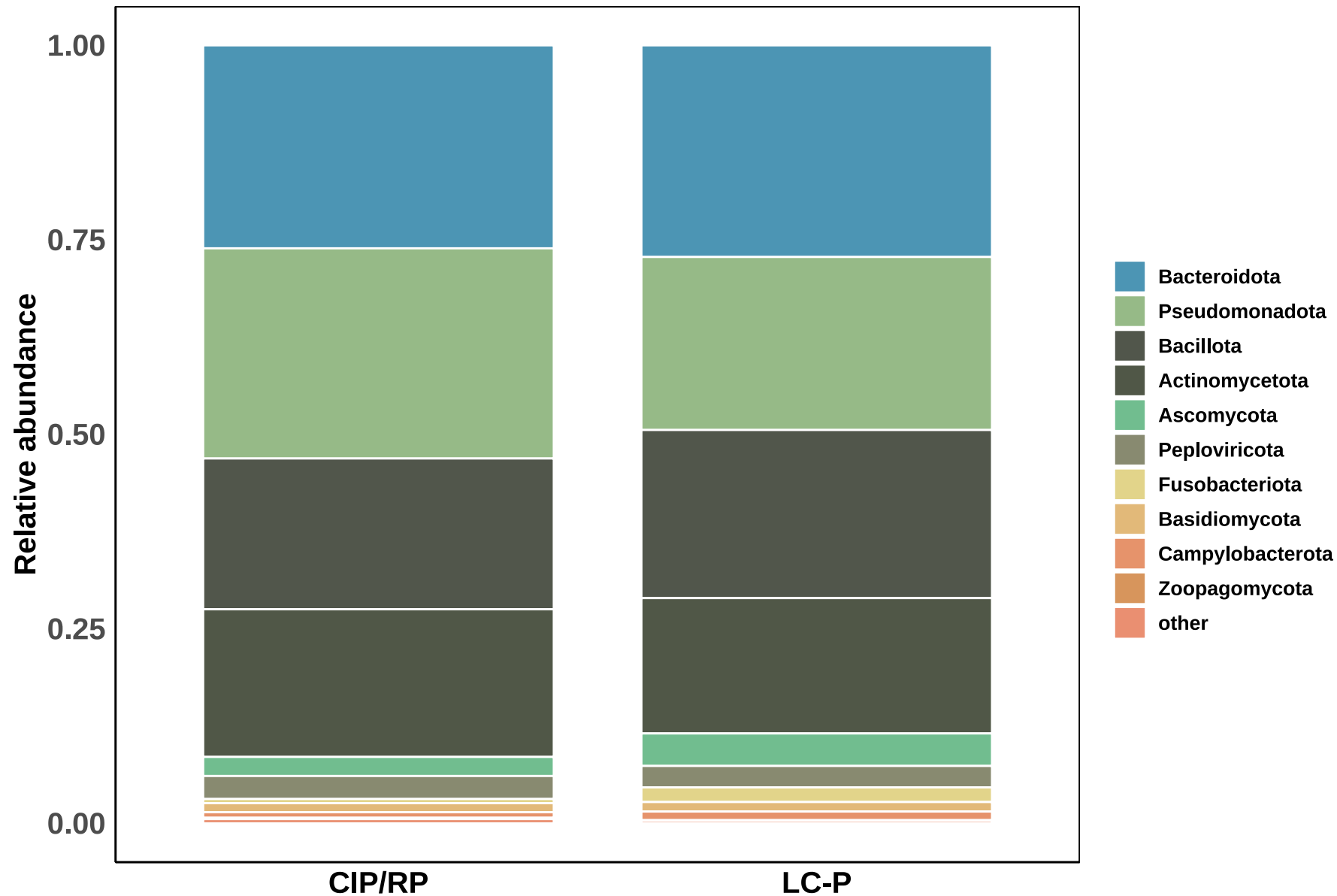

Supplement: Supplementary file 2 [file DataSheet1.zip › Data-all result/TaxonomicProfiling/Group_phylum_taxonomy_stacked_bar.pdf]

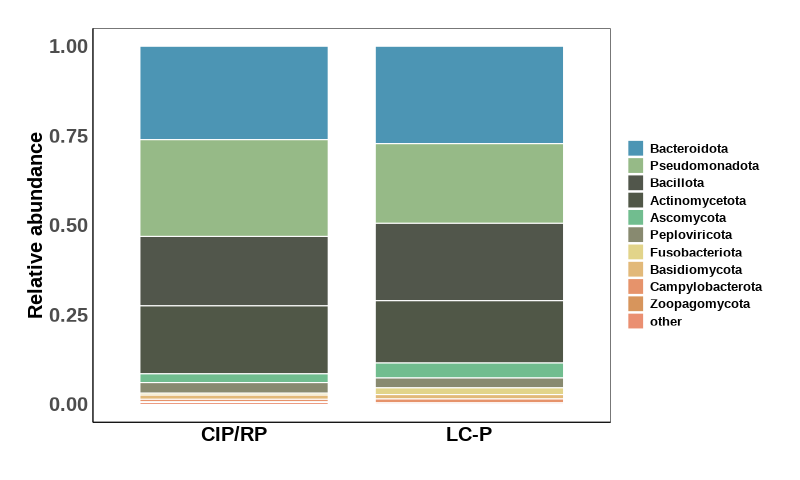

Supplement: Supplementary file 2 [file DataSheet1.zip › Data-all result/TaxonomicProfiling/Group_phylum_taxonomy_stacked_bar.png]

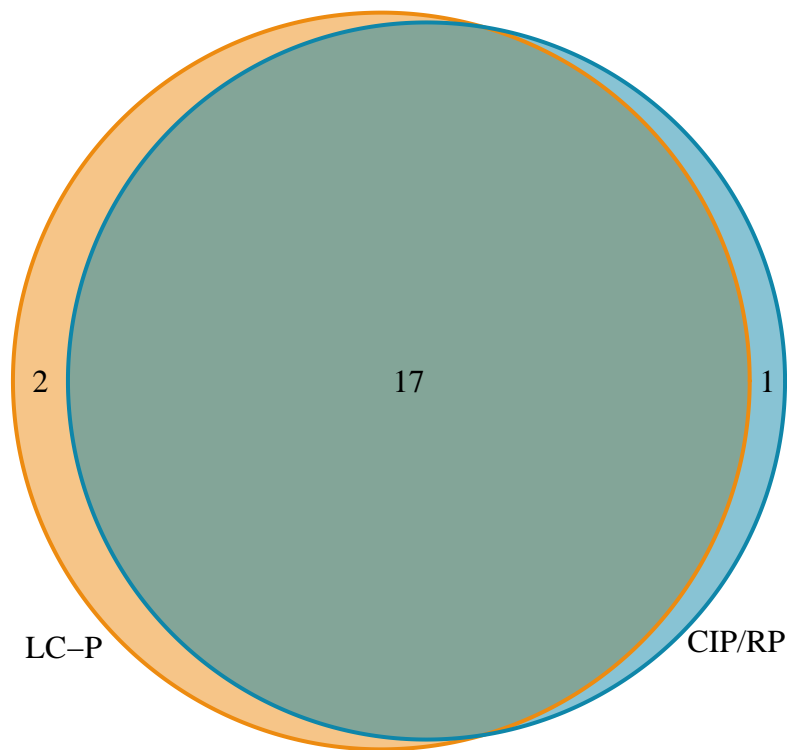

Supplement: Supplementary file 2 [file DataSheet1.zip › Data-all result/TaxonomicProfiling/Group_phylum_venn.pdf]

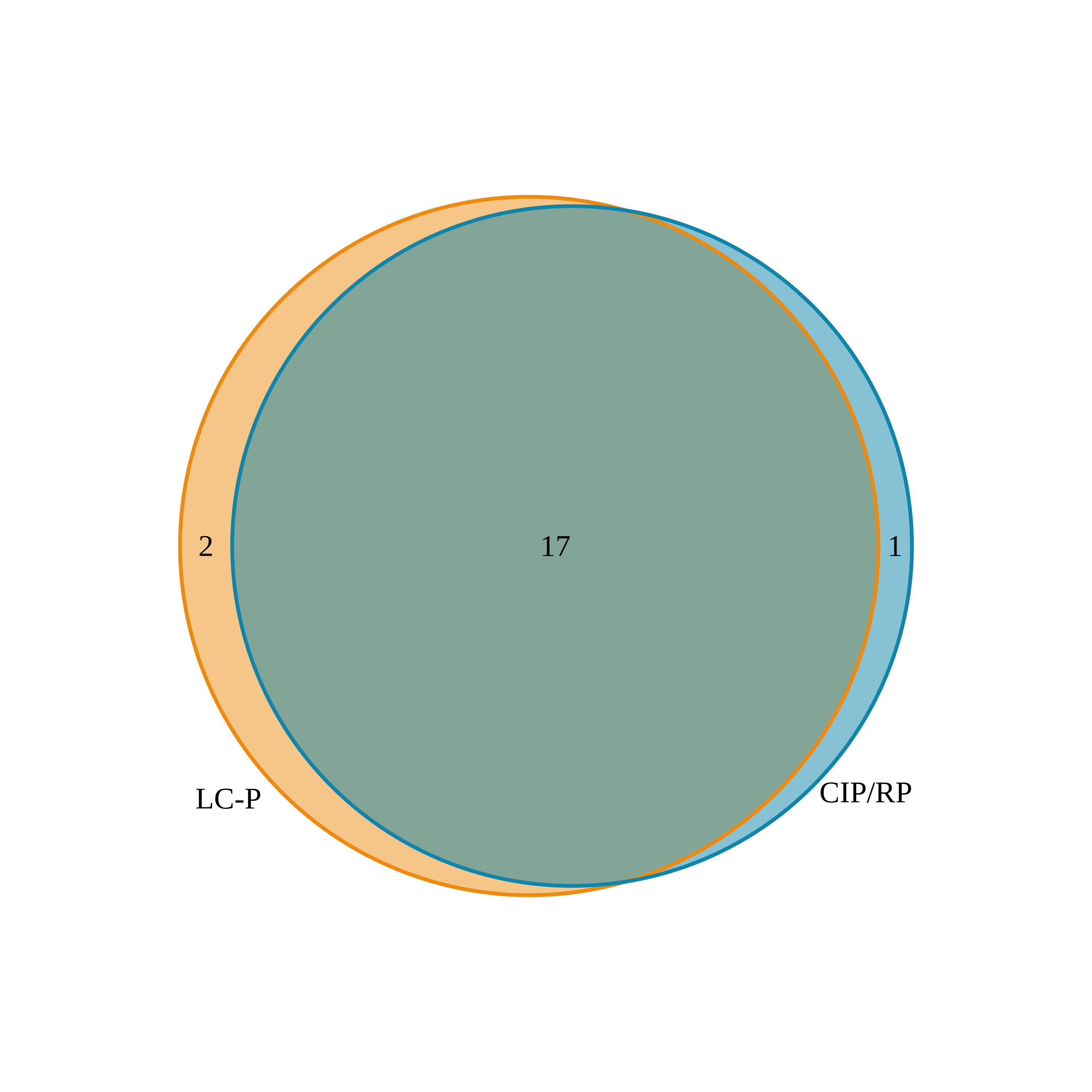

Supplement: Supplementary file 2 [file DataSheet1.zip › Data-all result/TaxonomicProfiling/Group_phylum_venn.png]

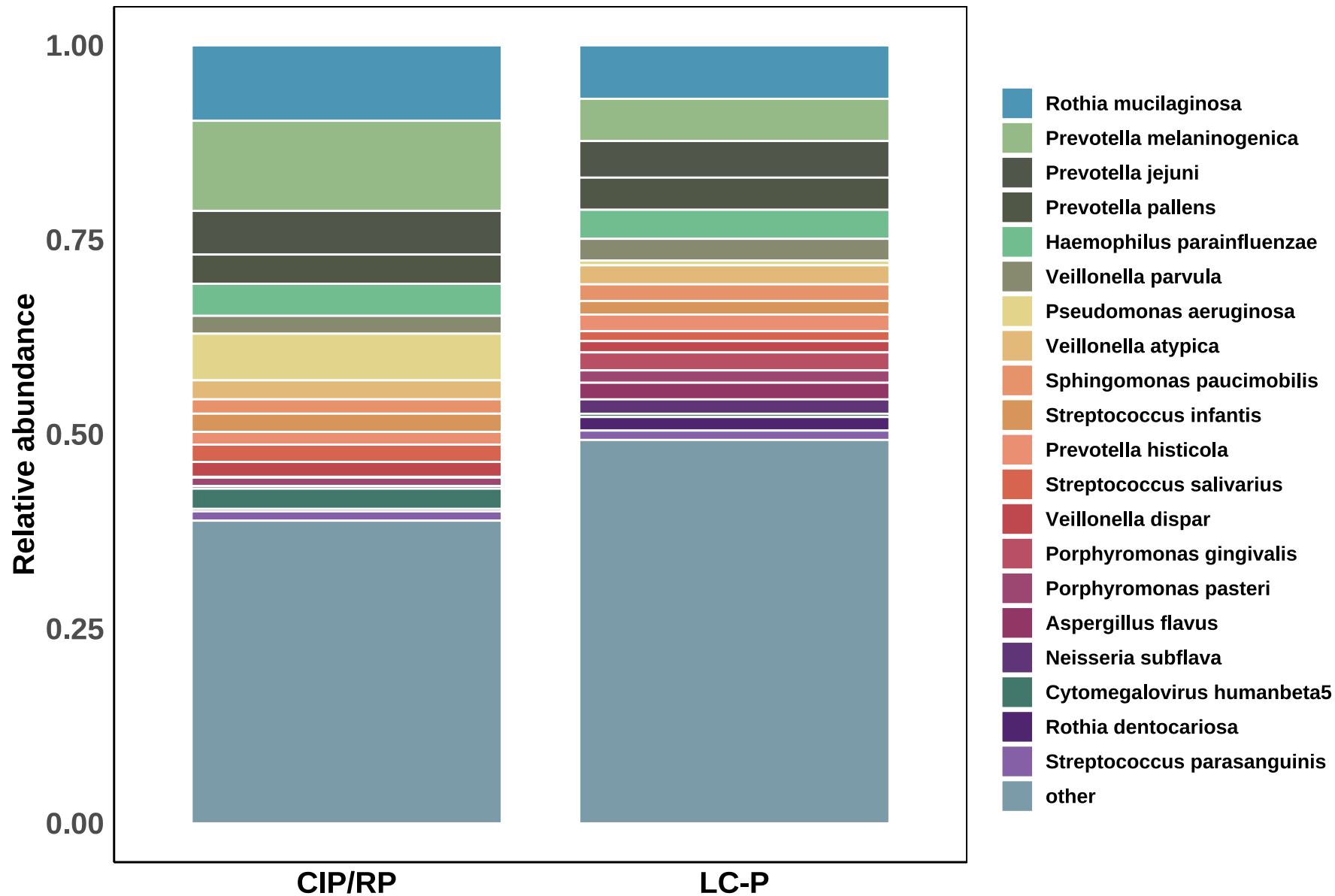

Supplement: Supplementary file 2 [file DataSheet1.zip › Data-all result/TaxonomicProfiling/Group_species_taxonomy_stacked_bar.pdf]

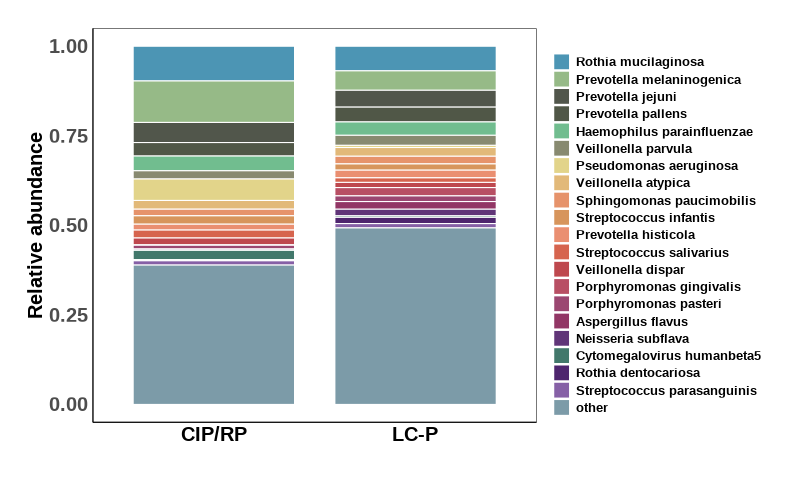

Supplement: Supplementary file 2 [file DataSheet1.zip › Data-all result/TaxonomicProfiling/Group_species_taxonomy_stacked_bar.png]

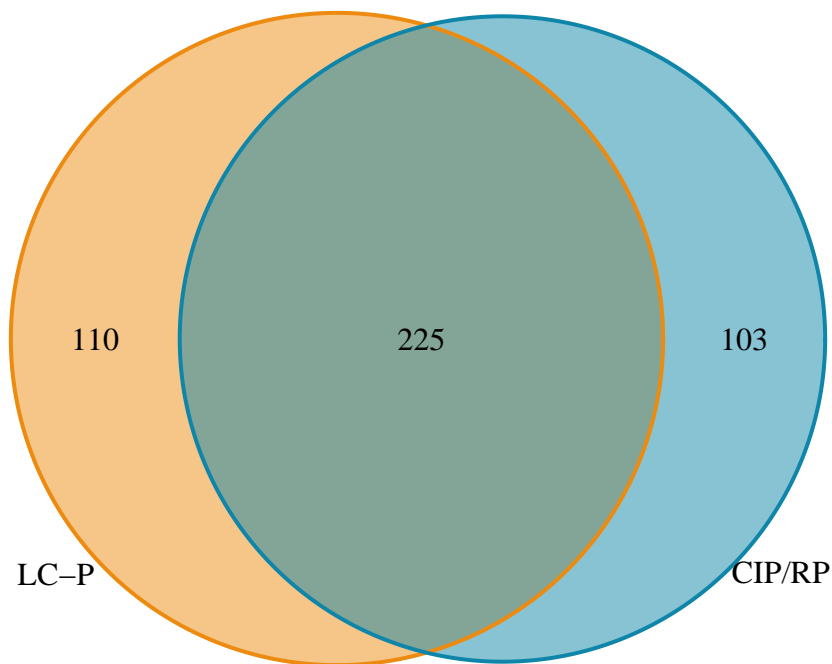

Supplement: Supplementary file 2 [file DataSheet1.zip › Data-all result/TaxonomicProfiling/Group_species_venn.pdf]

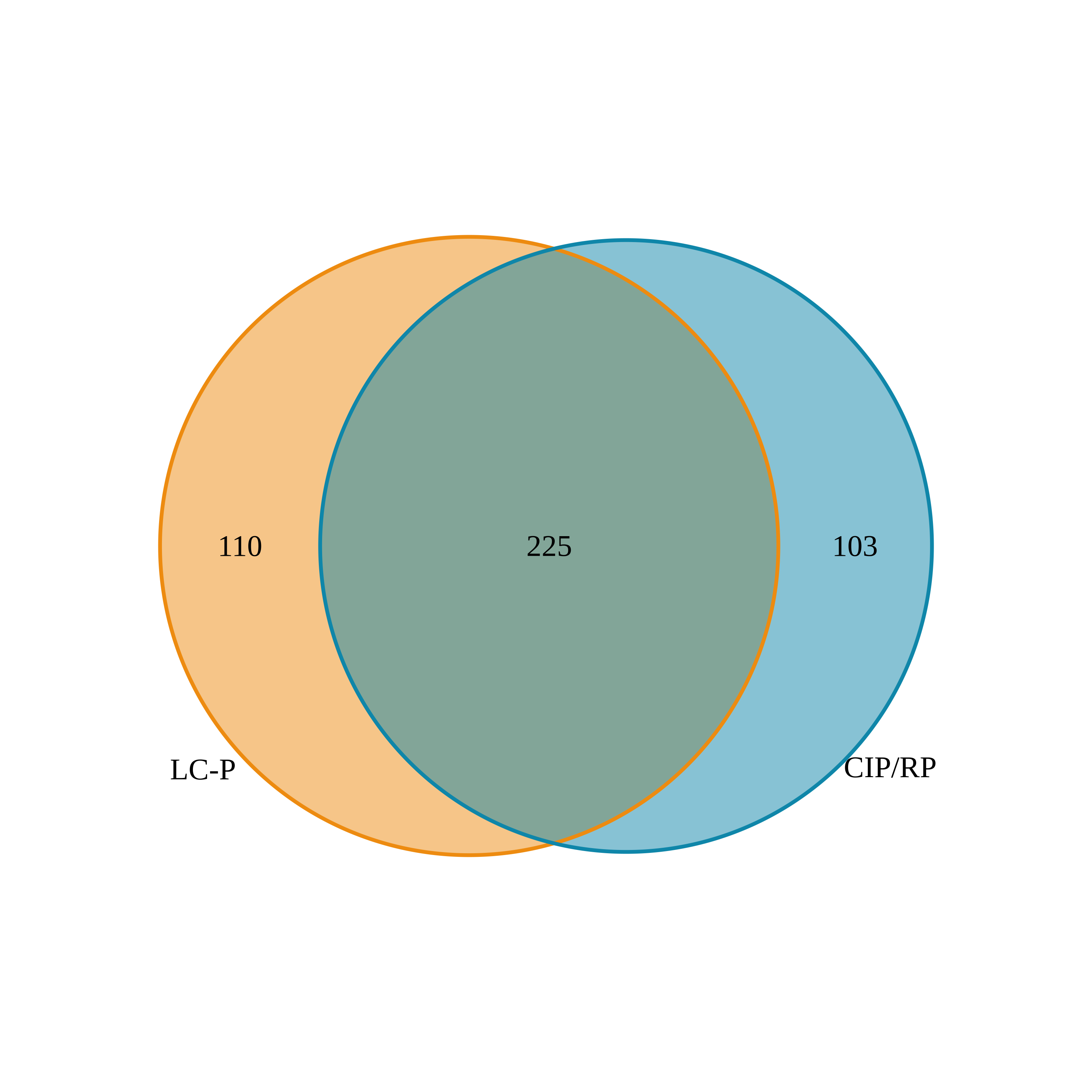

Supplement: Supplementary file 2 [file DataSheet1.zip › Data-all result/TaxonomicProfiling/Group_species_venn.png]

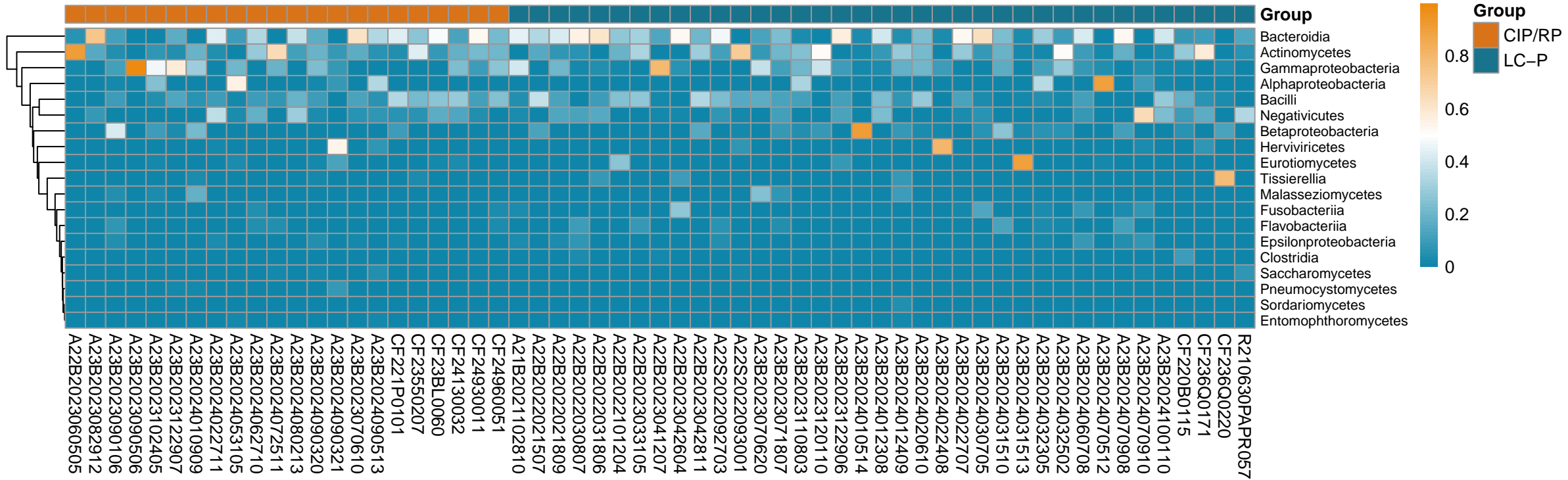

Supplement: Supplementary file 2 [file DataSheet1.zip › Data-all result/TaxonomicProfiling/Sample_class_taxonomy_heatmap.pdf]

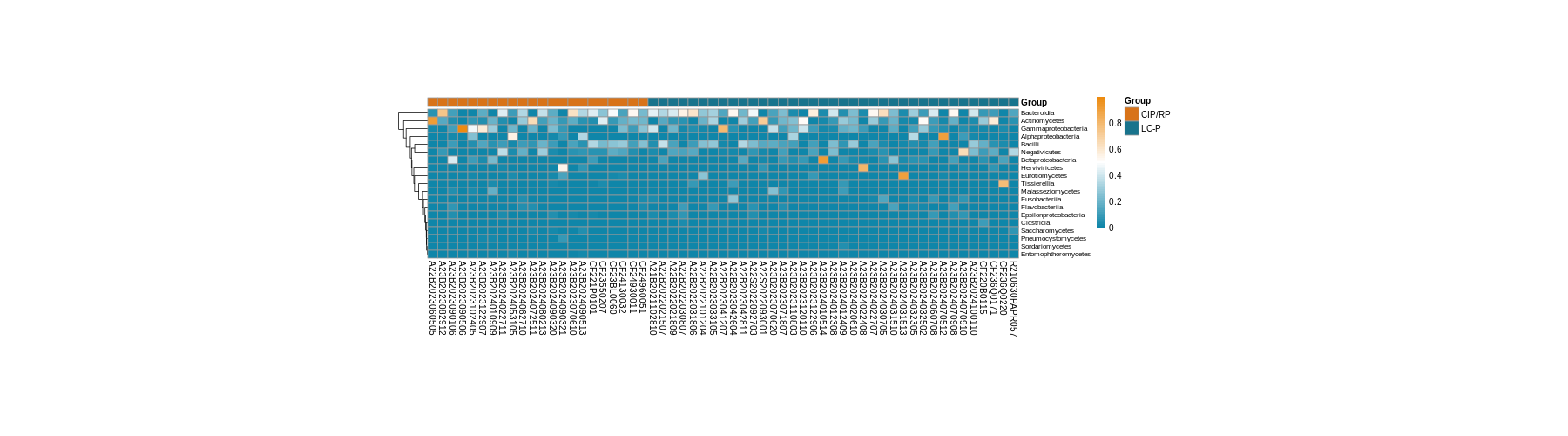

Supplement: Supplementary file 2 [file DataSheet1.zip › Data-all result/TaxonomicProfiling/Sample_class_taxonomy_heatmap.png]

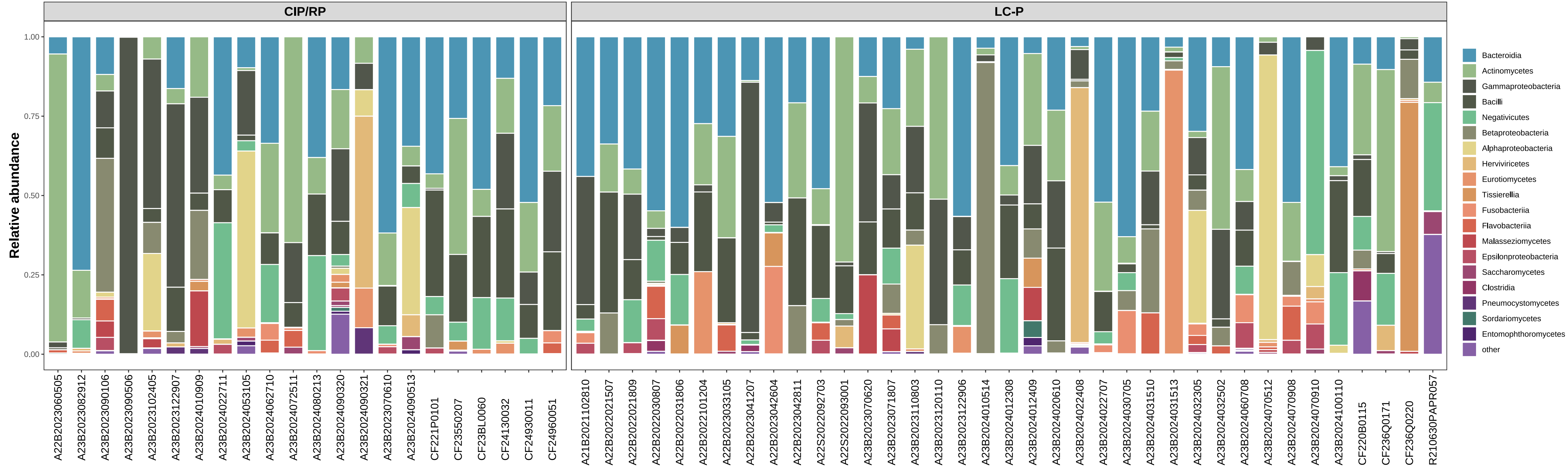

Supplement: Supplementary file 2 [file DataSheet1.zip › Data-all result/TaxonomicProfiling/Sample_class_taxonomy_stacked_bar.pdf]

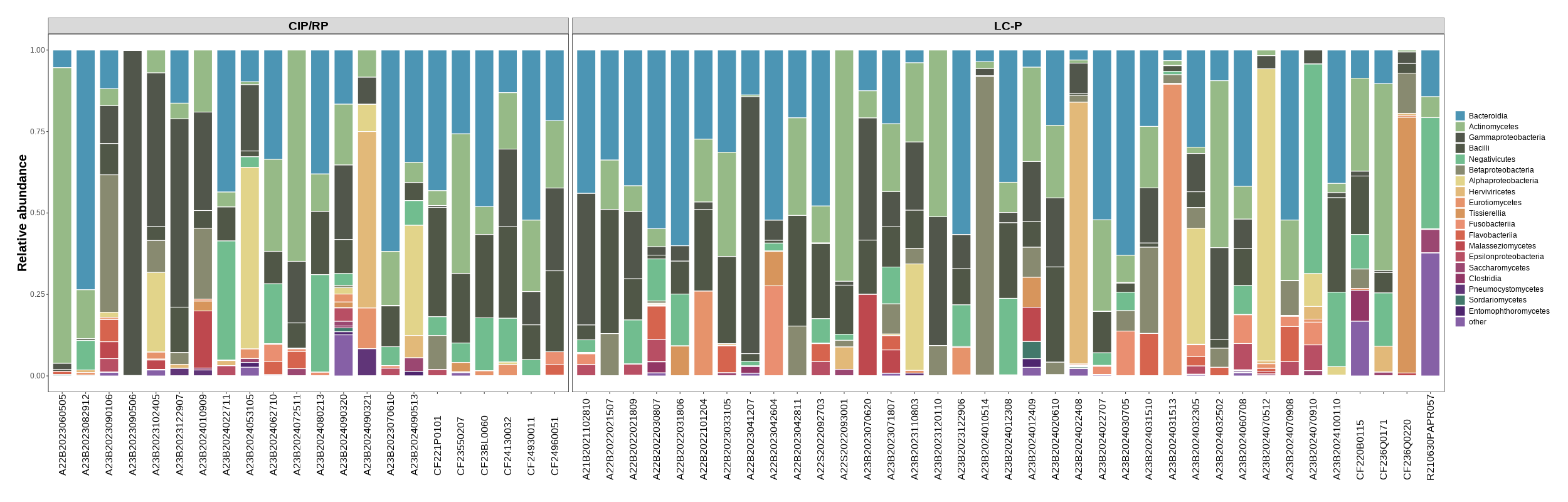

Supplement: Supplementary file 2 [file DataSheet1.zip › Data-all result/TaxonomicProfiling/Sample_class_taxonomy_stacked_bar.png]

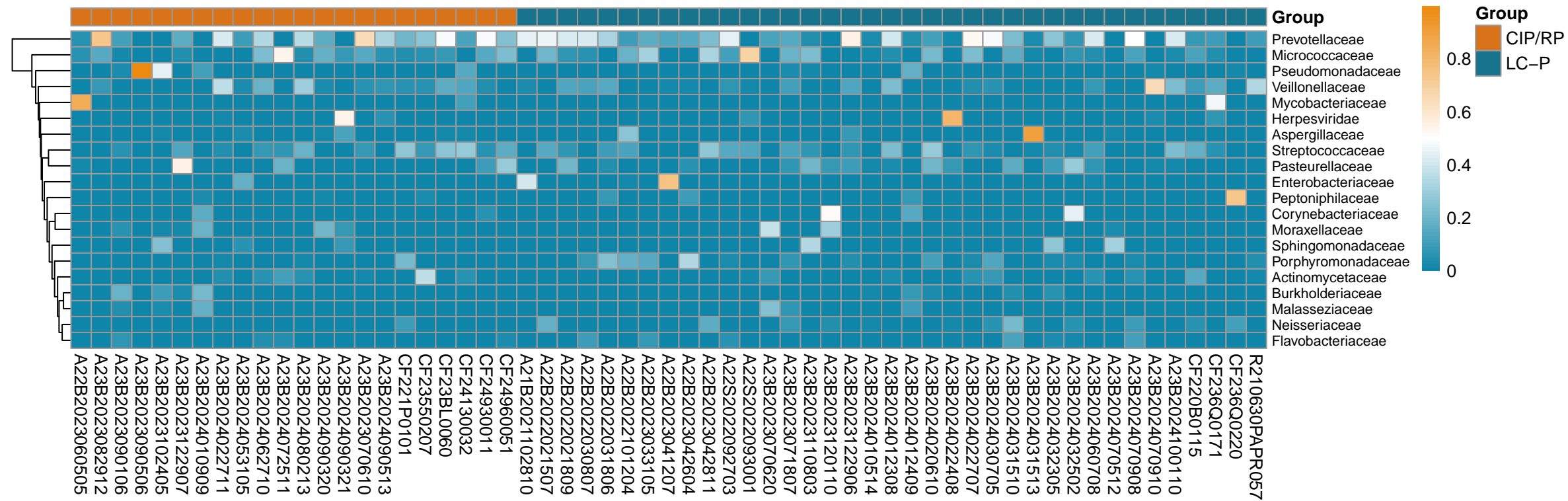

Supplement: Supplementary file 2 [file DataSheet1.zip › Data-all result/TaxonomicProfiling/Sample_family_taxonomy_heatmap.pdf]

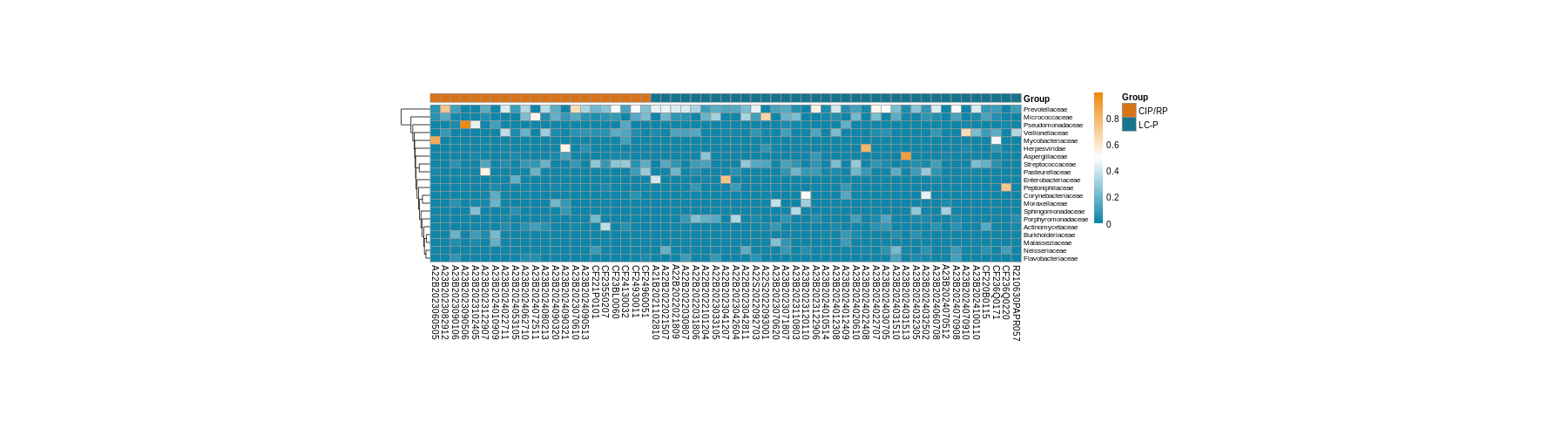

Supplement: Supplementary file 2 [file DataSheet1.zip › Data-all result/TaxonomicProfiling/Sample_family_taxonomy_heatmap.png]

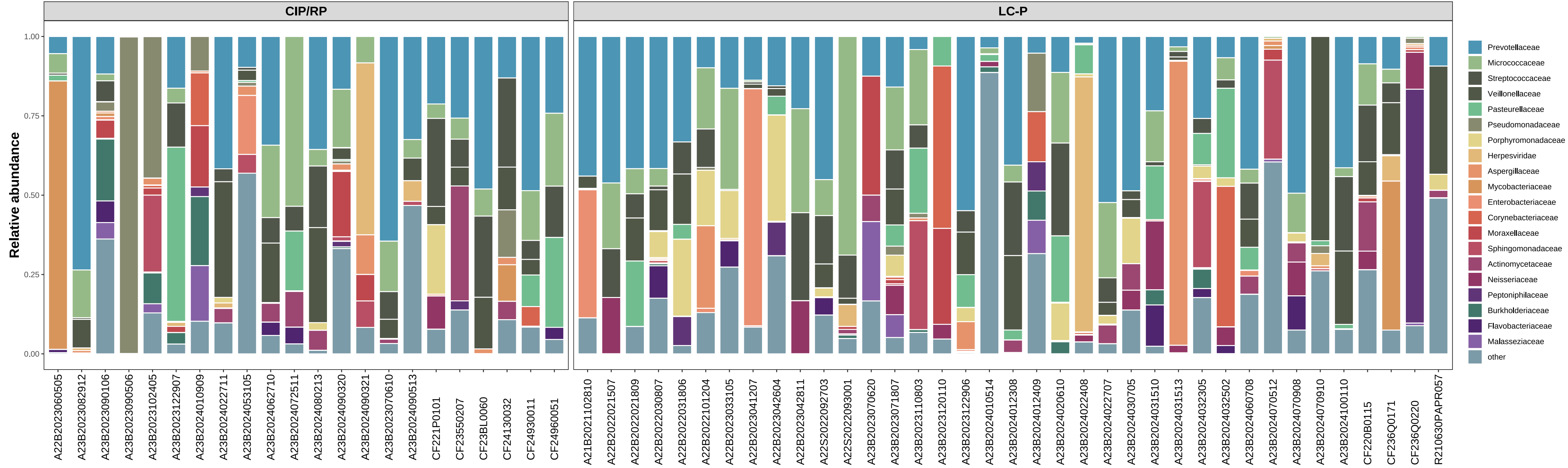

Supplement: Supplementary file 2 [file DataSheet1.zip › Data-all result/TaxonomicProfiling/Sample_family_taxonomy_stacked_bar.pdf]

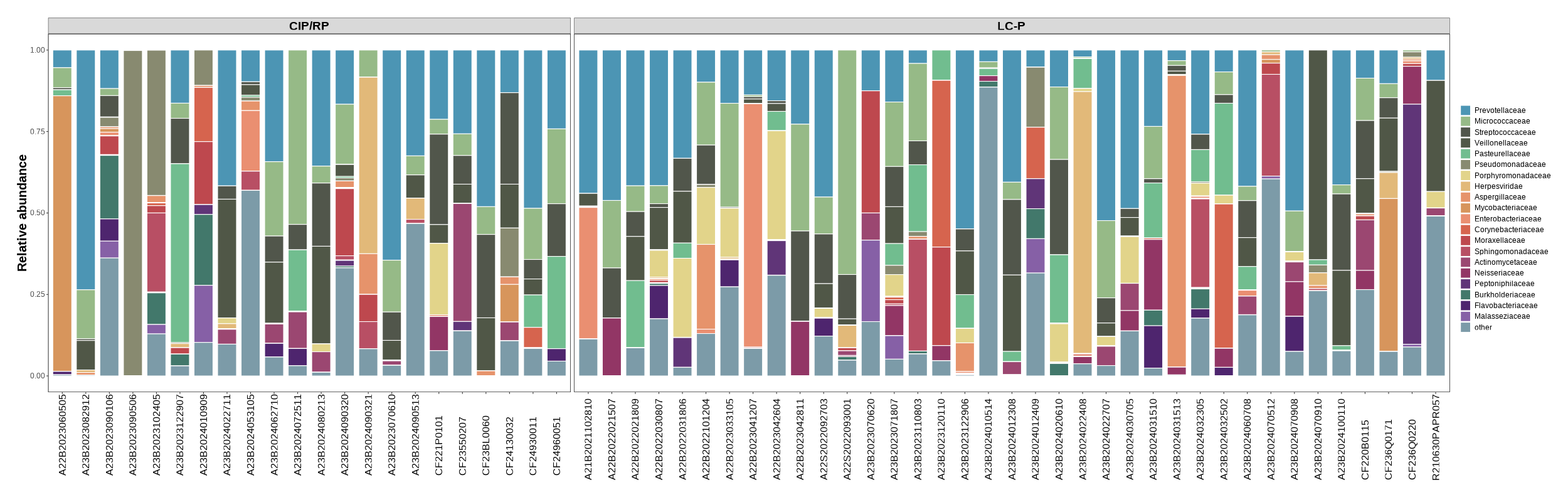

Supplement: Supplementary file 2 [file DataSheet1.zip › Data-all result/TaxonomicProfiling/Sample_family_taxonomy_stacked_bar.png]

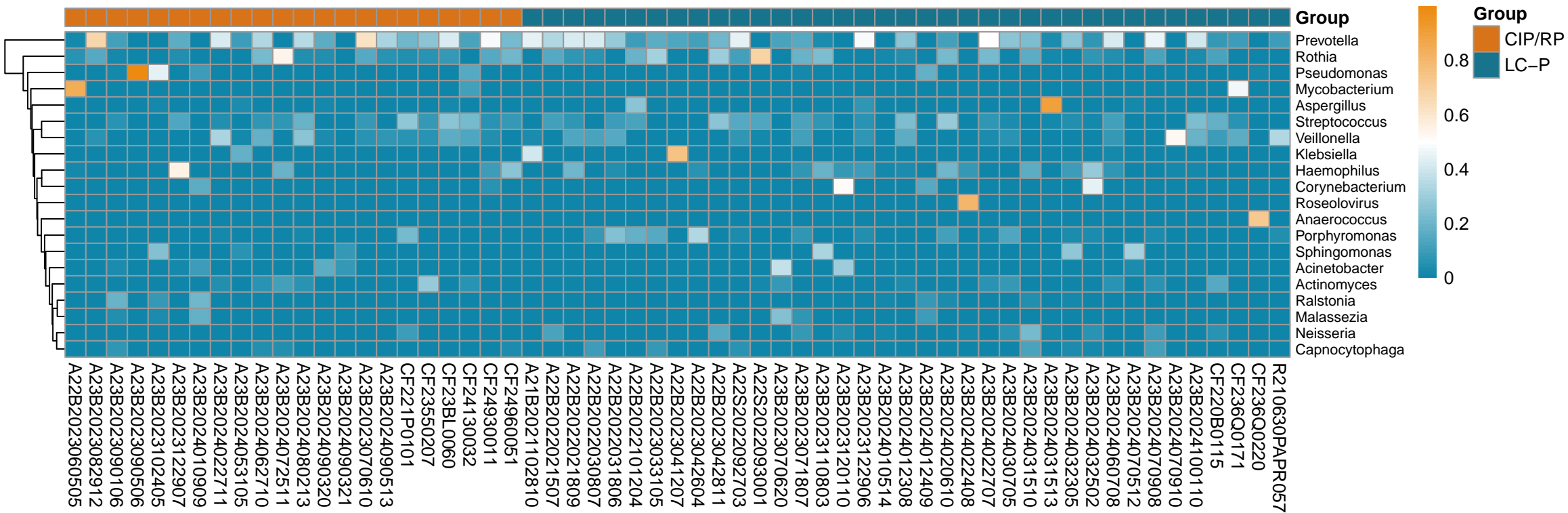

Supplement: Supplementary file 2 [file DataSheet1.zip › Data-all result/TaxonomicProfiling/Sample_genus_taxonomy_heatmap.pdf]

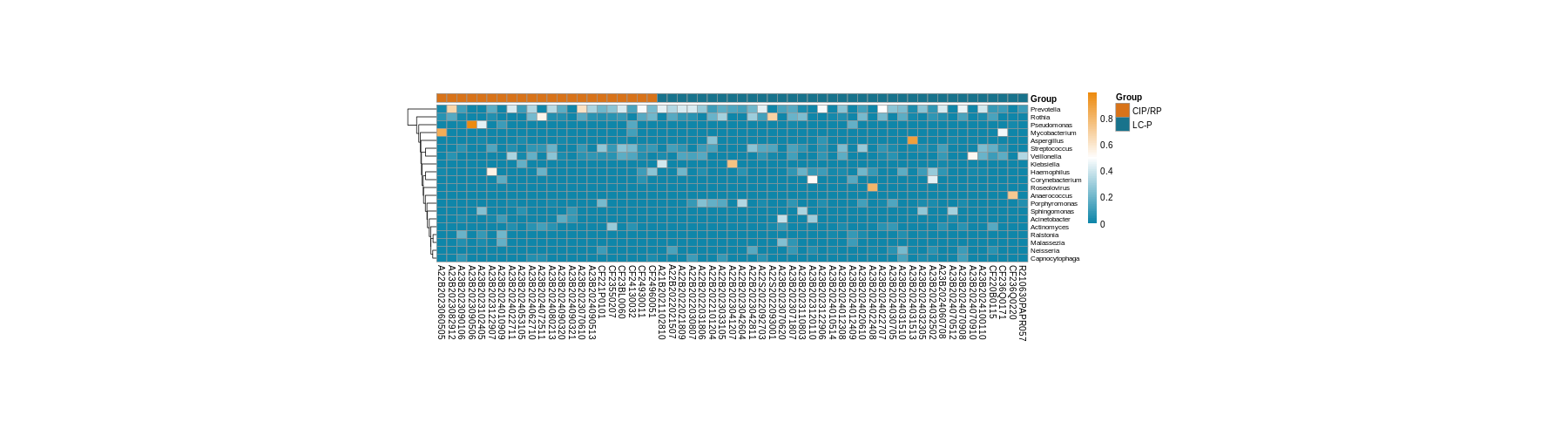

Supplement: Supplementary file 2 [file DataSheet1.zip › Data-all result/TaxonomicProfiling/Sample_genus_taxonomy_heatmap.png]

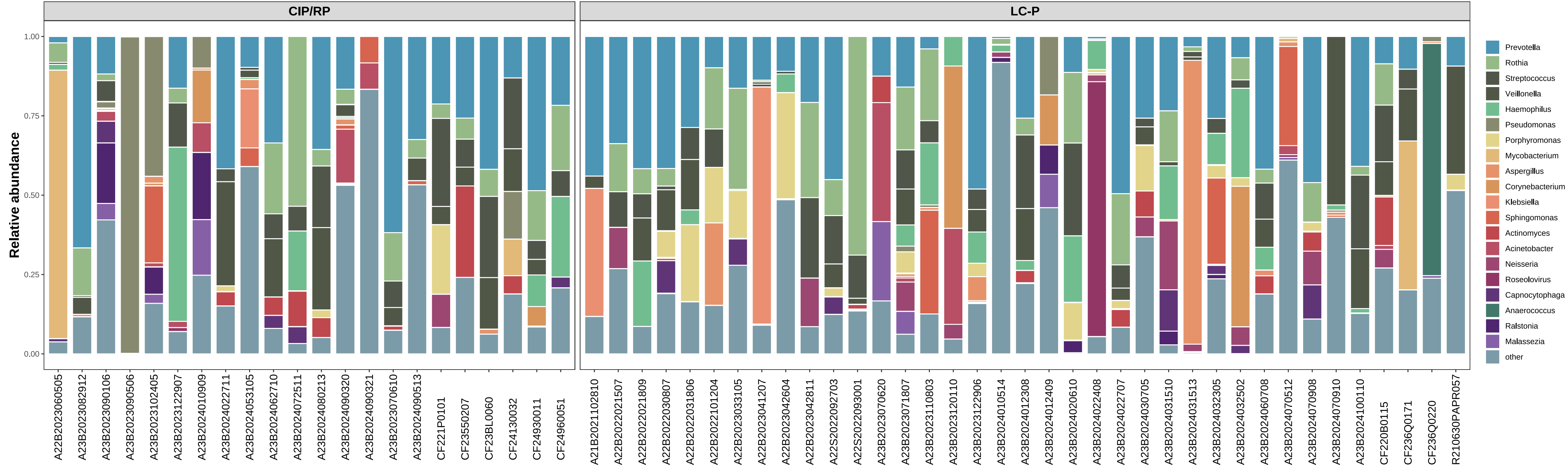

Supplement: Supplementary file 2 [file DataSheet1.zip › Data-all result/TaxonomicProfiling/Sample_genus_taxonomy_stacked_bar.pdf]

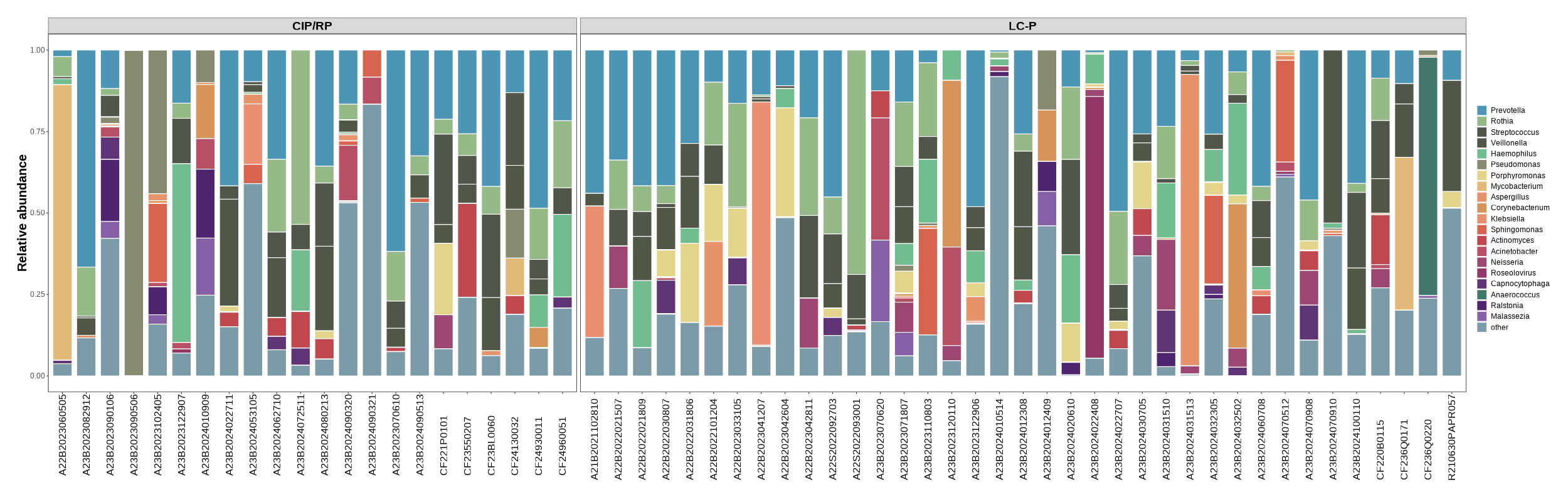

Supplement: Supplementary file 2 [file DataSheet1.zip › Data-all result/TaxonomicProfiling/Sample_genus_taxonomy_stacked_bar.png]
