# Supplementary material for: Enrichment of prevotella melaninogenica in the lower respiratory tract links to checkpoint inhibitor pneumonitis and radiation pneumonitis
Source: Front Cell Infect Microbiol. 2025 Oct 3;15:1594460. doi: 10.3389/fcimb.2025.1594460 (PMC12531214; doi:10.3389/fcimb.2025.1594460)
Supplement: Supplementary file 2 [file DataSheet1.zip › Data-all result/lefse/sample_lefse.Plot_Cladogram.pdf]

# Cladogram

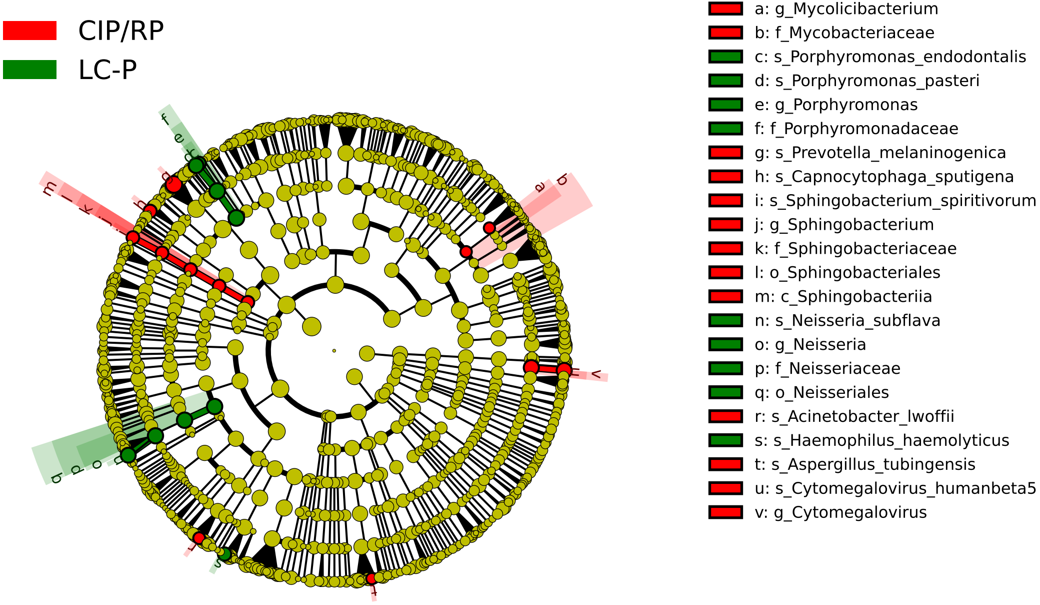
CIP/RP
LC-P
a: g_Mycolicibacterium
b: f_Mycobacteriaceae
c: s_Porphyromonas_endodontalis
d: s_Porphyromonas_pasteri
e: g_Porphyromonas
f: f_Porphyromonadaceae
g: s_Prevotella_melaninogenica
h: s_Capnocytophaga_sputigena
i: s_Sphingobacterium_spiritivorum
j: g_Sphingobacterium
k: f_Sphingobacteriaceae
l: o_Sphingobacteriales
m: c_Sphingobacteriia
n: s_Neisseria_subflava
o: g_Neisseria
p: f_Neisseriaceae
q: o_Neisseriales
r: s_Acinetobacter_lwoffii
s: s_Haemophilus_haemolyticus
t: s_Aspergillus_tubingensis
u: s_Cytomegalovirus_humanbeta5
v: g_Cytomegalovirus
