# Supplementary figures and images for: Enrichment of prevotella melaninogenica in the lower respiratory tract links to checkpoint inhibitor pneumonitis and radiation pneumonitis (part 2 of 2)
Source: Front Cell Infect Microbiol. 2025 Oct 3;15:1594460. doi: 10.3389/fcimb.2025.1594460 (PMC12531214; doi:10.3389/fcimb.2025.1594460)

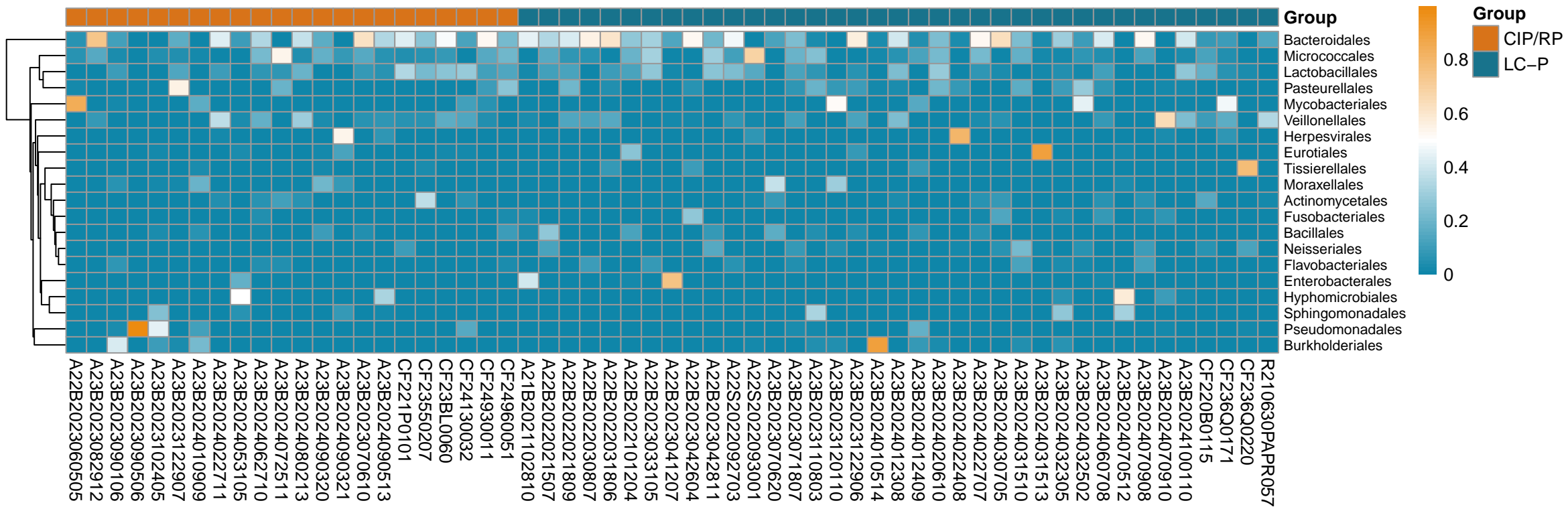

Supplement: Supplementary file 2 [file DataSheet1.zip › Data-all result/TaxonomicProfiling/Sample_order_taxonomy_heatmap.pdf]

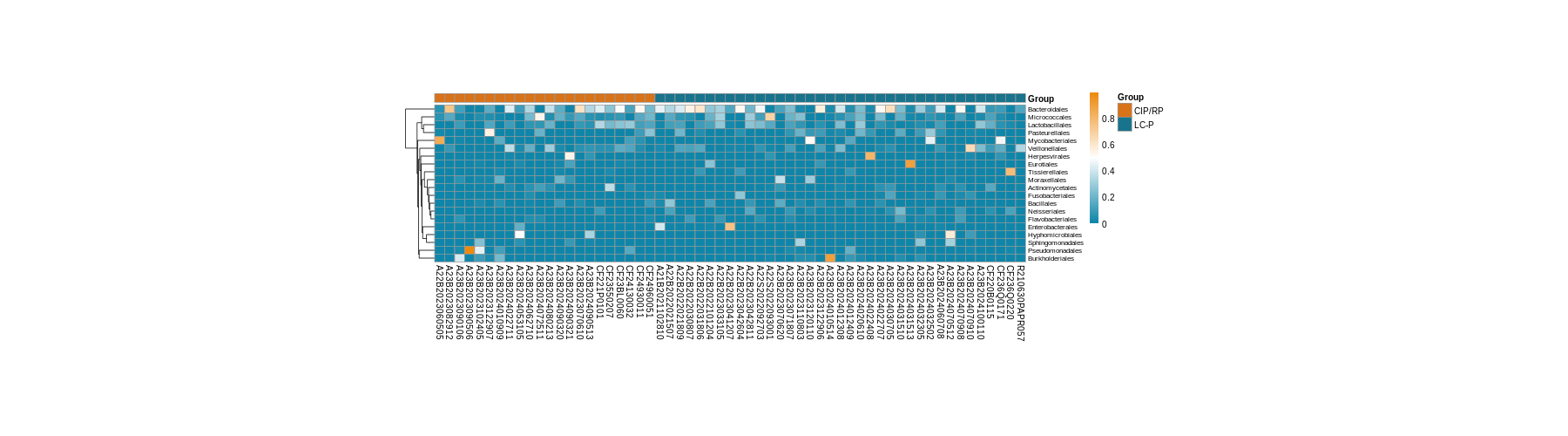

Supplement: Supplementary file 2 [file DataSheet1.zip › Data-all result/TaxonomicProfiling/Sample_order_taxonomy_heatmap.png]

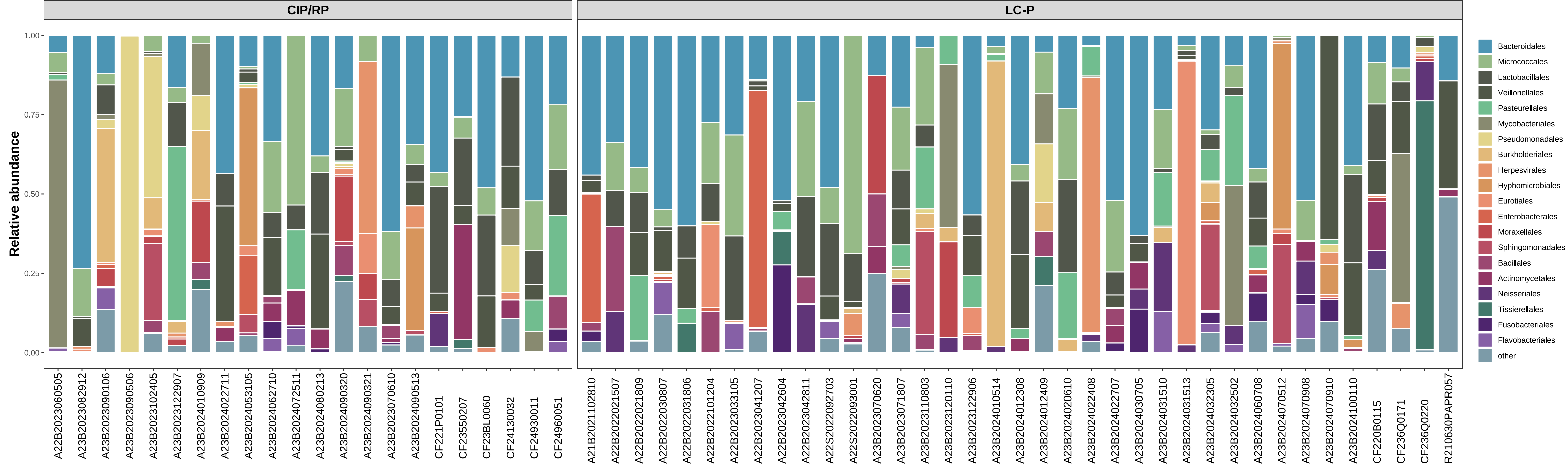

Supplement: Supplementary file 2 [file DataSheet1.zip › Data-all result/TaxonomicProfiling/Sample_order_taxonomy_stacked_bar.pdf]

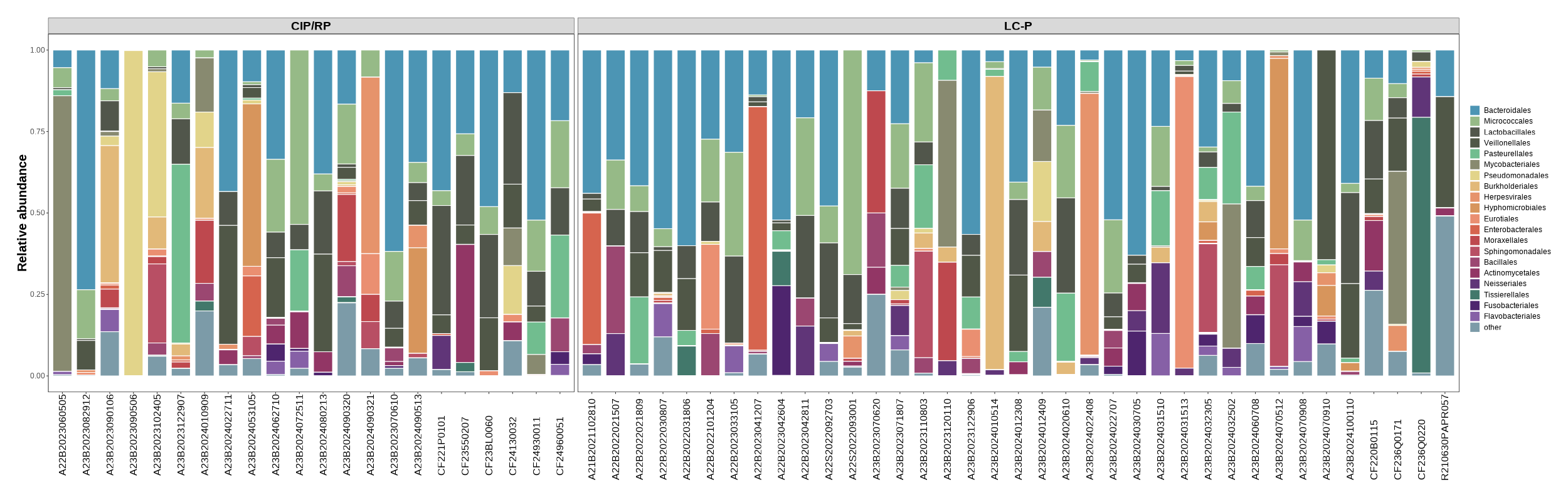

Supplement: Supplementary file 2 [file DataSheet1.zip › Data-all result/TaxonomicProfiling/Sample_order_taxonomy_stacked_bar.png]

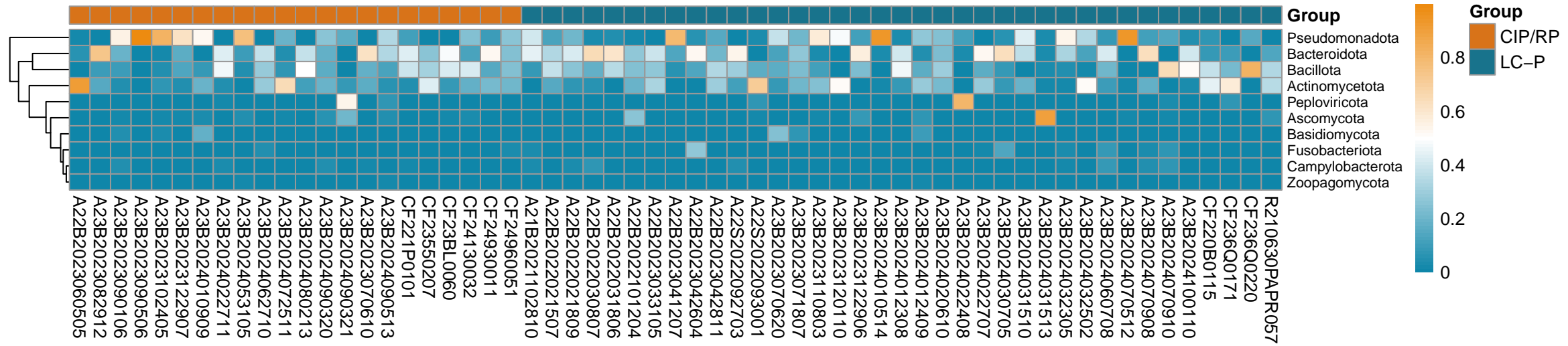

Supplement: Supplementary file 2 [file DataSheet1.zip › Data-all result/TaxonomicProfiling/Sample_phylum_taxonomy_heatmap.pdf]

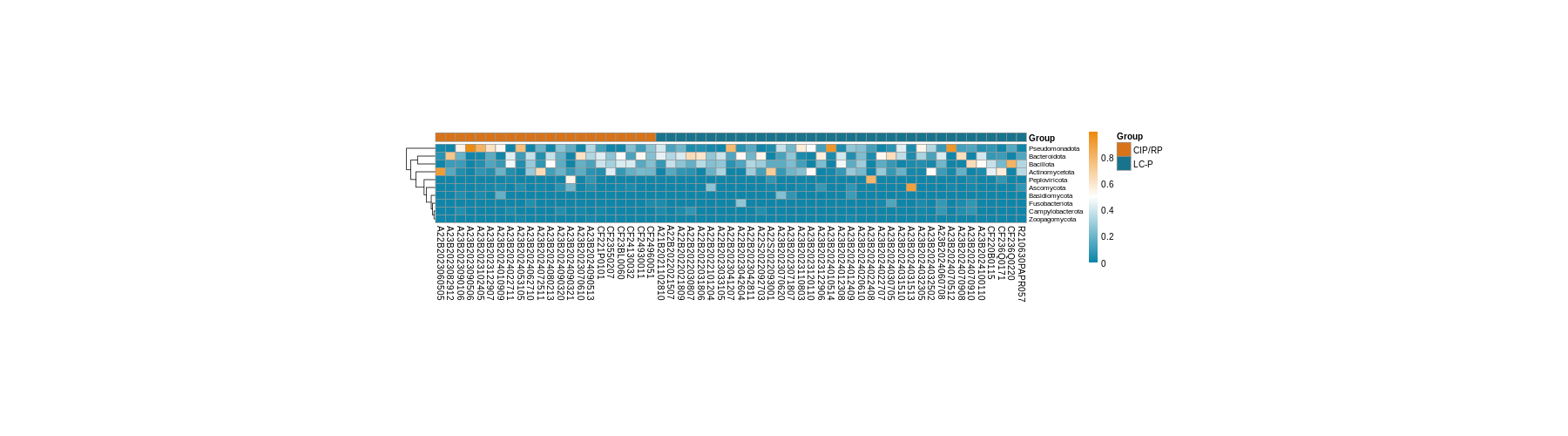

Supplement: Supplementary file 2 [file DataSheet1.zip › Data-all result/TaxonomicProfiling/Sample_phylum_taxonomy_heatmap.png]

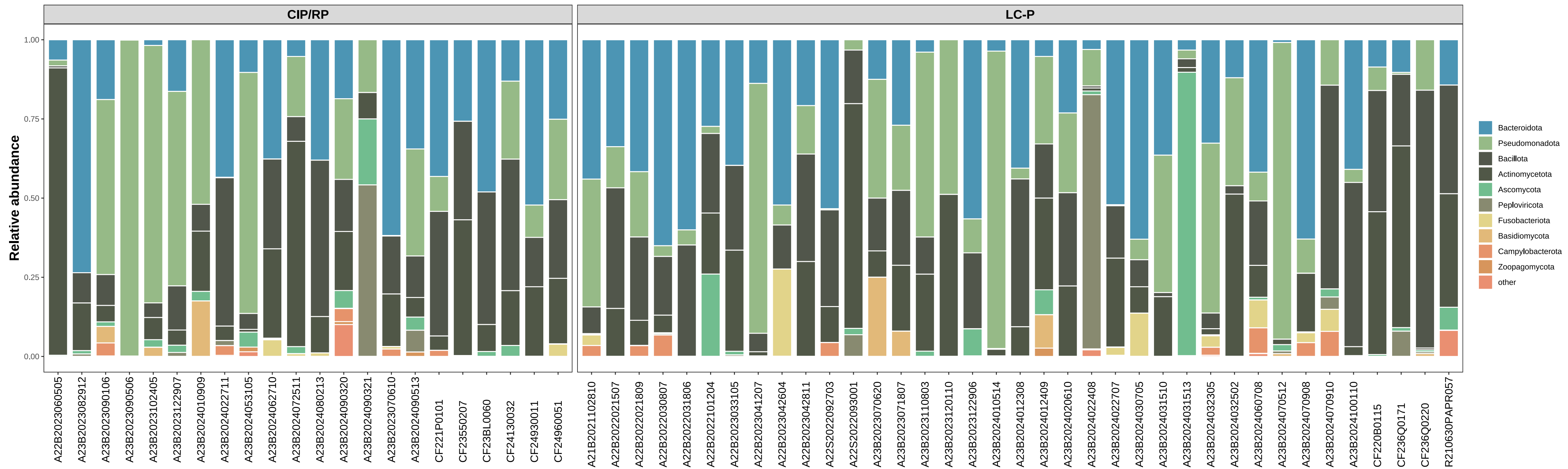

Supplement: Supplementary file 2 [file DataSheet1.zip › Data-all result/TaxonomicProfiling/Sample_phylum_taxonomy_stacked_bar.pdf]

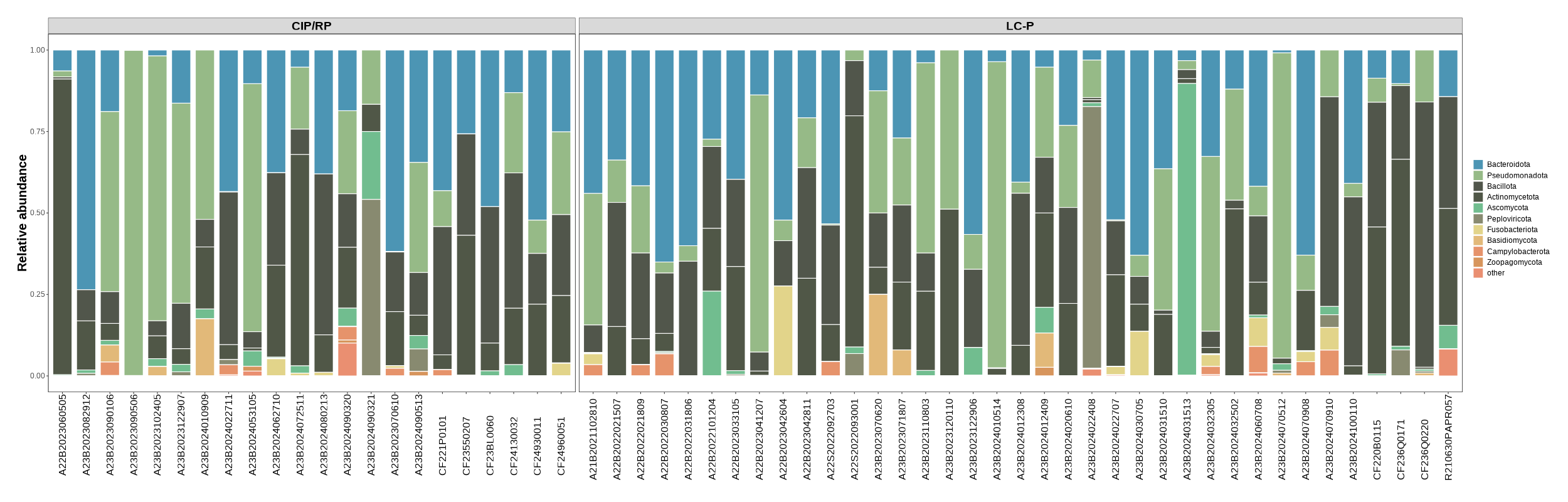

Supplement: Supplementary file 2 [file DataSheet1.zip › Data-all result/TaxonomicProfiling/Sample_phylum_taxonomy_stacked_bar.png]

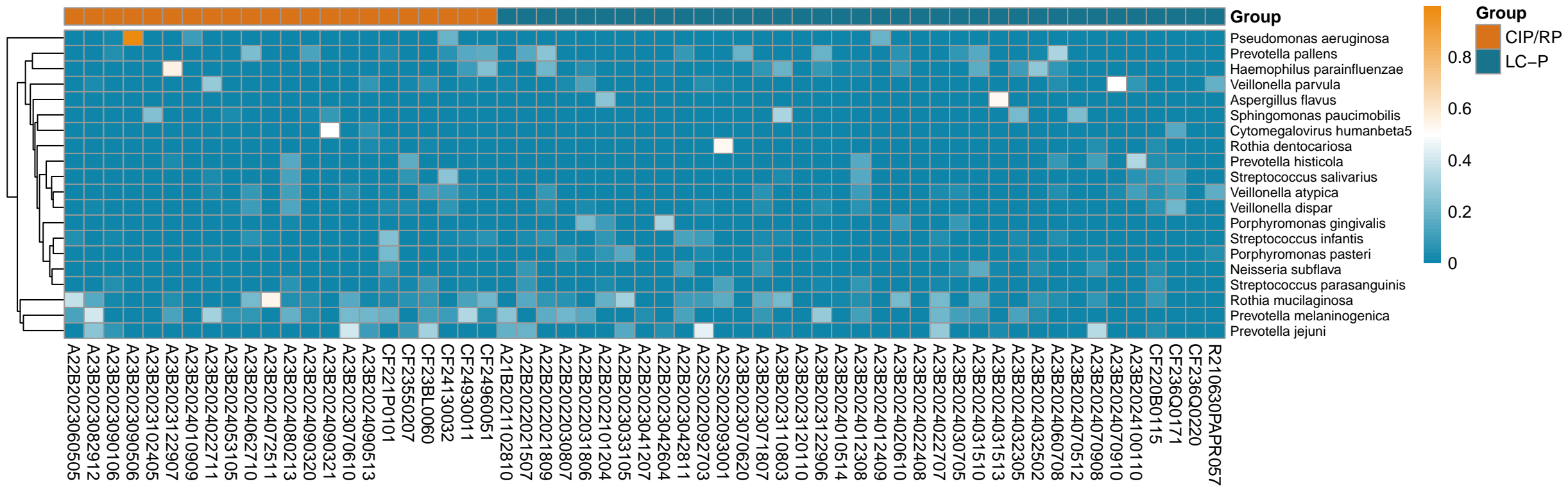

Supplement: Supplementary file 2 [file DataSheet1.zip › Data-all result/TaxonomicProfiling/Sample_species_taxonomy_heatmap.pdf]

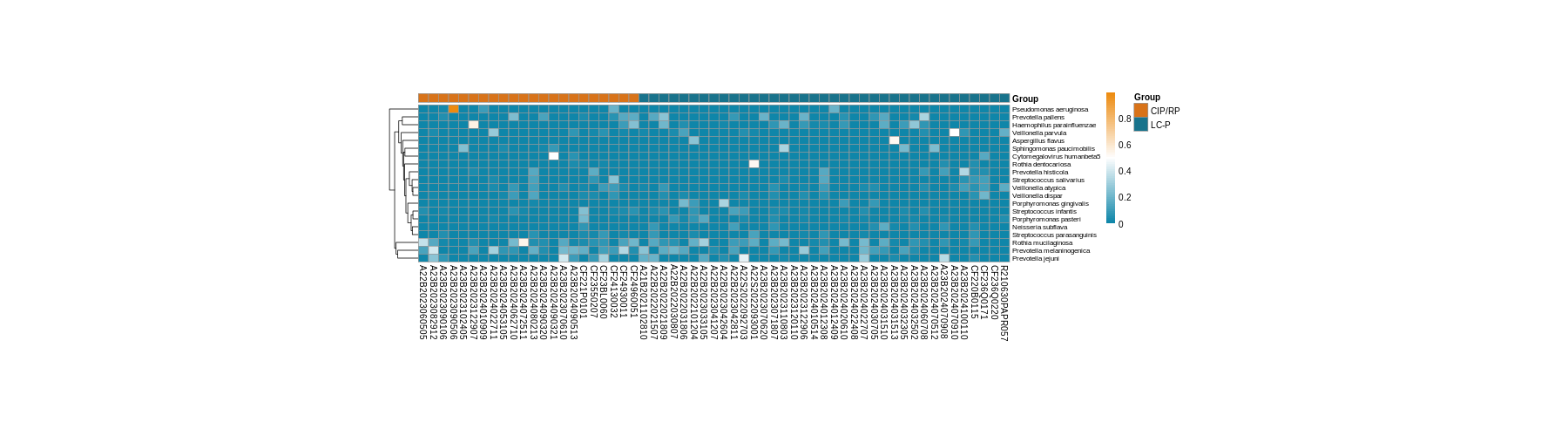

Supplement: Supplementary file 2 [file DataSheet1.zip › Data-all result/TaxonomicProfiling/Sample_species_taxonomy_heatmap.png]

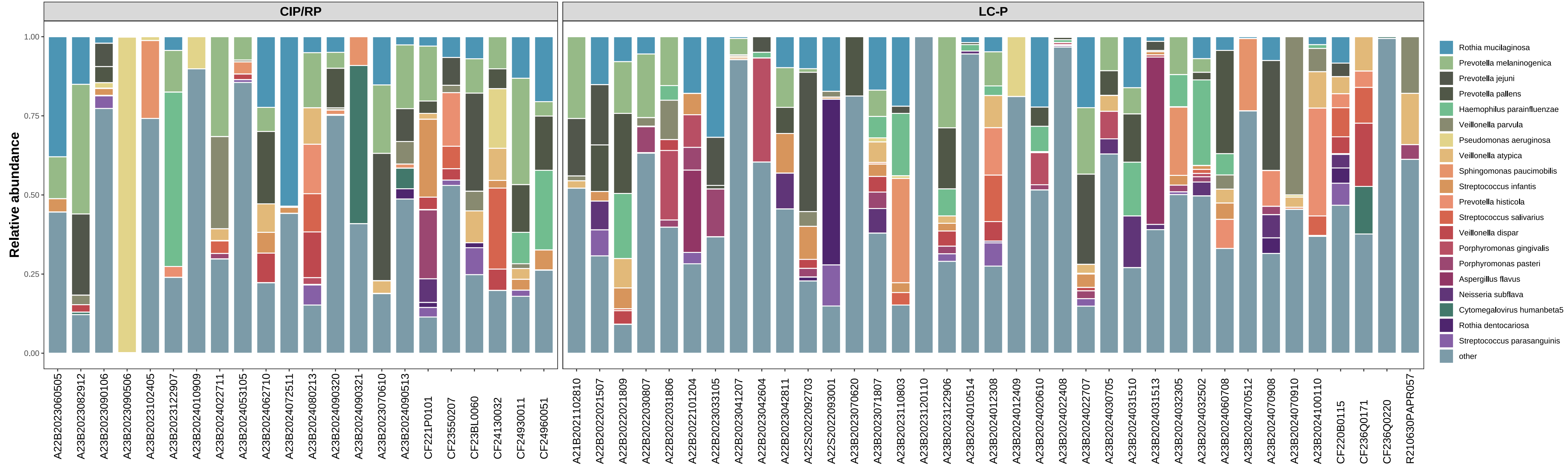

Supplement: Supplementary file 2 [file DataSheet1.zip › Data-all result/TaxonomicProfiling/Sample_species_taxonomy_stacked_bar.pdf]

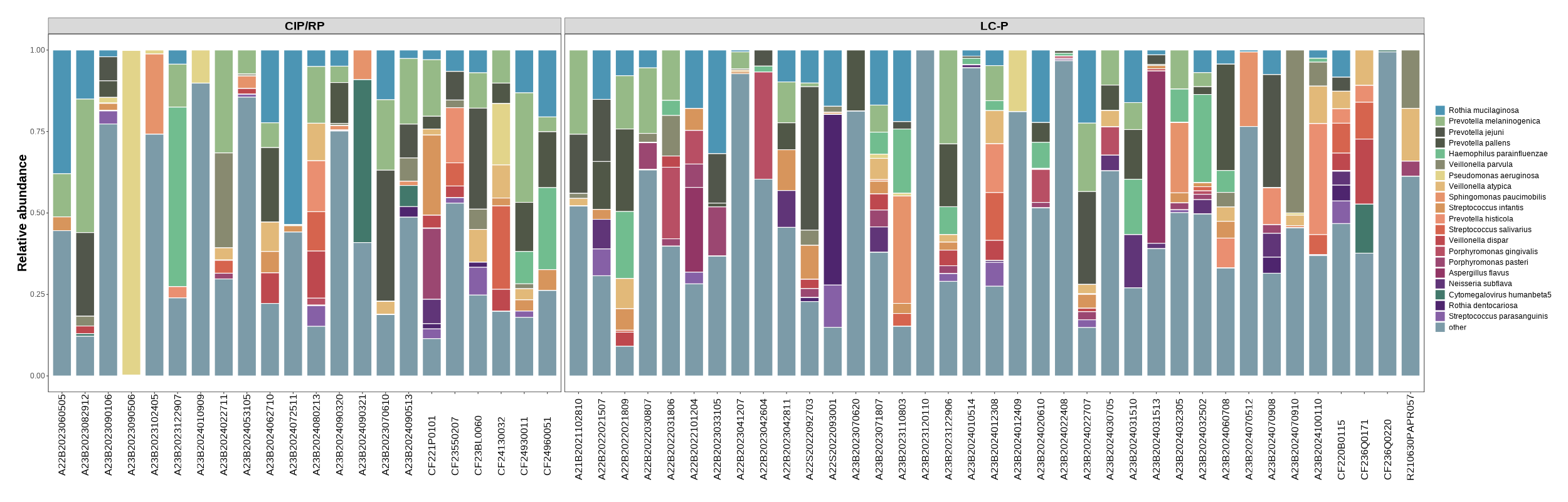

Supplement: Supplementary file 2 [file DataSheet1.zip › Data-all result/TaxonomicProfiling/Sample_species_taxonomy_stacked_bar.png]
